# Supplementary material for: Developing a behavioural intervention package to identify and amend incorrect penicillin allergy records in UK general practice and subsequently change antibiotic use
Source: BMJ Open. 2020 Oct 1;10(10):e035793. doi: 10.1136/bmjopen-2019-035793 (PMC7534681; doi:10.1136/bmjopen-2019-035793)
Supplement: Supplementary data [file bmjopen-2019-035793supp001.pdf]

SUPPLEMETARY MATERIALS

S1. Behavioural Analysis Table AlABAMA

S2. Think-aloud topic guides for clinicians and patients

S3. Table of changes: "Penicillin Allergy Testing: going for a test" booklet

S4. Full description of the AlABAMA (ALlergy AntiBiotics And Microbial resistAnce) Intervention package in accordance with the Template for Intervention Description and Replication (TIDieR) framework

S5. GUIDED – a guideline for reporting for intervention development studies.

S1. Behavioural Analysis Table

Target Behaviours:

- Clinician referral of low risk patients to undertake the Penicillin Allergy Testing (PAT)
- Clinician prescription of penicillin after negative test result
- Patient attendance at Penicillin Allergy Testing (PAT)
- Patient consumption of penicillin when prescribed after negative test result

Notes: [QI]: Qualitative Interviews; [RR]: Rapid Review; [LR]: Literature Review; PIS; Participant Information Sheet

Table S1a Behavioural Analysis Table for the AlABAMA intervention, clinician behaviours

**Barriers / *facilitators* to target behaviours**	**Intervention Component/s**	**Intervention ingredient**	**Theoretical Domains Framework (TDF)**	**Target construct (BCW)**	**Intervention function (BCW)**	**Behaviour Change Technique (using 93 BCT taxonomy v1)**
**Target behaviour: Clinician referral of low risks patients to Penicillin Allergy Testing**						
**Lack of knowledge** about incidence of **incorrect penicillin allergy labels** [QI]	"Penicillin Allergy Testing: Information for general practice" leaflet<br><br>Training	• Provide information on penicillin allergy records and its implications	Knowledge	Psychological capability	Education	4.2 information about antecedents
**Lack of knowledge** of how **accurate** the tests are [QI]	"Penicillin Allergy Testing: Information for general practice" leaflet	• Provide information that the oral challenge test is the gold standard test for penicillin allergy	Knowledge	Psychological capability	Education	4.2 information about antecedents
**Lack of knowledge** of the existence of **allergy services** or what they involve [QI]	"Penicillin Allergy Testing: Information for general practice" leaflet<br><br>Training	• Provide information what the penicillin allergy test involves and where it will take place	Knowledge	Psychological capability	Education	4.2 information about antecedents
**Poor experience** of PAT [QI, RR]	"Penicillin Allergy Testing: Information for general practice" leaflet	• Provide information about why it is important to have correct penicillin allergy labels and what the test involve	Skills<br><br>Behavioural regulation	Physical capability<br><br>Psychological Capability	Training<br><br>Education<br><br>Persuasion	2.7 feedback on outcome of behaviour
**Lack of understanding** PAT procedures and results [QI, RR]	"Penicillin Allergy Testing: Information for general practice" leaflet<br><br>Training	• Provide information about the three stages of penicillin allergy testing and what negative and positive results mean	Knowledge	Psychological capability	Education	4.2 information about antecedents

**Lack of guidelines** in learning about **benefits and risks** of allergy services [QI, RR]	"Penicillin Allergy Testing: Information for general practice" leaflet	• Provide a series of references around penicillin allergy and PAT, including NICE guidelines	Knowledge	Psychological capability	Education	9.1 credible source
**Lack of guidance on referral** [QI, RR]	"Penicillin Allergy Testing: Information for general practice" leaflet<br><br>Training and WIs	• Provide information about the NICE guidelines<br>• Provide a system to refer low risks patients to do testing	Knowledge<br><br>Skills	Psychological capability<br><br>Physical skills	Education<br><br>Training	1.4 action planning<br><br>4.1 instructions on how to perform a behaviour
**Lack of unified approach** for referral of patients to PAT [RR]	"Penicillin Allergy Testing: Information for general practice" leaflet<br><br>Training and WIs	• Provide references on NICE guidelines on referral<br>• Provide a system to refer low risks patients to do testing	Skills<br><br>Social influences	Physical skills<br><br>Social opportunity	Training<br><br>Modelling	1.4 action planning<br><br>4.1 instructions on how to perform a behaviour
**Lack of knowledge** regarding the **oral challenge test** [QI]	"Penicillin Allergy Testing: Information for general practice" leaflet<br><br>Training	• Provide information about the oral challenge test (stage 3 or PAT), why it is done, what the procedure is and how accurate it is	Knowledge	Psychological capability	Education	4.2 information about antecedents
Perception of **overburdening the allergy service** [QI]	"Penicillin Allergy Testing: Information for general practice" leaflet	• Provide information around NICE guidelines in support of PAT	Social/Professional role and identity<br><br>Knowledge	Social opportunity<br><br>Psychological capability	Modelling<br><br>Enablement<br><br>Training	9.1 credible source<br><br>6.3 information about others' approval

**Lack of follow up** after referral [QI]	Pop-up alert<br><br>“Penicillin Allergy Testing: Information for general practice” leaflet<br><br>Training	• Provide information that they will receive confirmation about the patients’ results	Environmental Context and resources	Physical opportunity	Environmental restructuring<br><br>Enablement	7.1 prompts/cues<br><br>2.7 feedback on outcome of behaviour
Expectations that **patients may not want to be tested** [RR]	Consultations with patients<br><br>“Penicillin allergy testing: going for a test” booklet	• Provide pre-test booklet to discuss with patients<br>• Provide the opportunity to discuss the benefits of the test during the consultation	Social influences	Social opportunities	Enablement	3.2 social support (practical)<br><br>21.2 restructuring of social environment
Concerns that the test might be **expensive** [QI]	PAT procedures at clinic	• Provide support to referral of patients from colleagues and practice managers<br>• Provide the PAT testing procedures	Social/Professional role and identity	Social opportunities	Enablement<br><br>Modelling	3.2 social support (practical)
Fear of **getting it wrong** and the safety patients [QI]	Training<br><br>PAT procedures in clinic	• Provide support to refer low risk patients to do PAT<br>• Provide evidence of safety of PAT	Social/Professional role and identity<br><br>Beliefs about consequences<br><br>Emotion	Social opportunities<br><br>Reflective motivation<br><br>Automatic motivation	Enablement<br><br>Modelling<br><br>Education<br><br>Persuasion	5.1 information about health consequences<br><br>5.6 information about emotional consequences
Perception that it is **safer to take patients’ word for it** [QI]	“Penicillin Allergy Testing: Information for general practice” leaflet	• Provide evidence that only a small percentage of patients who think to be allergic actually are	Beliefs about consequences<br><br>Emotions	Reflective motivation<br><br>Automatic motivation	Education<br><br>Persuasion	5.1 information about health consequences

Fear of **getting it wrong** and **professional ramifications** [*QI]	Training<br><br>PAT procedures in clinic	• Provide support to refer low risk patients to do PAT	Social/Professional role and identity<br><br>Environmental context and resources<br><br>Emotion	Social opportunities<br><br>Physical opportunity<br><br>Automatic motivation	Enablement<br><br>Modelling	3.2 social support (practical)
**Lack of time** during consultation to discuss referral [RR]	Consultation with patients<br><br>Training	• Provide the opportunity to have a consultation with patients to specifically discuss PAT	Environmental context and resources<br><br>Beliefs about capabilities	Physical opportunities<br><br>Reflective motivation	Enablement	12.2 restructuring of social environment
Anticipation that **patients will not want** to have a reaction [RR]	Consultations with patients<br><br>"Penicillin allergy testing: going for a test" booklet	• Provide the opportunity to discuss with patients the risks of taking the test<br>• Provide pre-test booklet with information around the risks of PAT	Social influences	Social opportunity	Enablement	12.2 restructuring of social environment
**Forgetting to discuss** testing [RR]	Consultations with patients<br><br>Training	• Provide the opportunity to have consultations specifically around PAT	Behavioural regulation	Psychological capability	Enablement	3.2 social support (practical)
**Not knowing** that patients had an **allergy** [RR]	Consultations with patients	• Provide the opportunity to discuss their medical history with patients	Knowledge	Psychological capability	Training	2.7 feedback on outcomes of behaviour
**Lack of access** to testing [RR]	PAT testing procedures in clinic<br><br>"Penicillin Allergy Testing: Information for general practice" leaflet	• Provide the information that they can refer low risks patients to undertake PAT<br>• Provide information about where the test will be and what the stages are	Environmental Context and resources<br><br>Knowledge	Physical opportunity<br><br>Psychological capability	Enablement<br><br>Training	5.3 information of social and environmental consequences<br><br>15.1 verbal persuasion of capabilities

**Lack of ability in drawing a conclusion** on allergy status from medical history [RR]	"Penicillin Allergy Testing: Information for general practice" leaflet	• Provide information about the differences between drug reaction and allergy	Knowledge	Psychological capability	Training	4.2 information about antecedents
**Limited knowledge** of drug **allergies** [QI, RR]	"Penicillin Allergy Testing: Information for general practice" leaflet	• Provide information on how an allergy reaction is different than drug reaction	Knowledge	Psychological capability	Training	4.2 information about antecedents
**Poor knowledge** of allergy skin **testing** [RR]	"Penicillin Allergy Testing: Information for general practice" leaflet	• Provide information about skin testing (stage 2 of PAT), what the test will include, how patients will be monitored and how the results will be interpreted in relation to stage 3.	Knowledge	Psychological capability	Training	2.7 feedback on outcome of behaviour
**Lack of confidence** in dealing with allergies [RR]	"Penicillin Allergy Testing: Information for general practice" leaflet	• Provide information about penicillin allergy records and its implication	Beliefs about capabilities	Reflective motivation	Education<br><br>Persuasion	5.1 information about health consequences
Ready **availability of alternate antibiotics** [RR]	"Penicillin Allergy Testing: Information for general practice" leaflet	• Provide information on standards of care and use of broad-spectrum antibiotics	Beliefs about consequences	Reflective motivation	Education<br><br>Persuasion	5.1 information about health consequences
Awareness that **CCG are "cash strapped"** [QI]	PAT procedures at clinic	• Provide allergy testing at the hospital	Environmental context and resources	Environmental Context and resources	Physical opportunity<br><br>Enablement	12.2 restructuring of social environment
Worries about **costs of tests** [*QI]	PAT procedures at clinic	• Provide support in referring patients to PAT	Environmental context and resources	Environmental Context and resources	Physical opportunity<br><br>Enablement	12.2 restructuring of social environment

**Referral takes time** [QI]	PAT procedures at clinic<br>Training and WIs	• Provide guidance about referral and time of responses	Environmental context and resources	Environmental Context and resources	Physical opportunity<br><br>Enablement	4.1 instructions on how to perform a behaviour
**Patients not aware that the allergy label** may have a negative consequences [QI]	Consultations with patients	• Provide the opportunity to discuss the effects of inaccurate allergy labels	Social Influences	Social opportunity	Enablement	12.2 restructuring of social environment
Lack **of support from colleagues** in changing the label [QI]	Training and WIs	• Provide support from lead GPs and colleagues about changing the labels	Social influences	Social opportunity	Modelling	3.2 social support (practical)
**Experience of prescribing an alternative antibiotic** as routine [QI]	Training<br><br>“Penicillin Allergy Testing: Information for general practice” leaflet	• Provide information about how to refer low risks patients<br>• Provide information on the benefits of taking penicillin	Behavioural regulation	Psychological capability	Enablement	4.1 instruction on how to perform a behaviour
*Perception of how **easy finding an alternative antibiotic** is [QI]*	“Penicillin Allergy Testing: Information for general practice” leaflet	• Provide information about the benefits of taking penicillin<br>• Provide information about the effects of broad spectrum antibiotics	Knowledge	Psychological capability	Education<br><br>Enablement	5.1 information about health consequences
*Awareness that patients have been **historically over diagnosed** with penicillin allergy [QI]*	“Penicillin Allergy Testing: Information for general practice” leaflet<br><br>Training	• Provide information that incorrect penicillin allergy records are very common	Knowledge<br><br>Memory	Psychological capability	Education<br><br>Training	4.2 information about antecedents
*Awareness that **records might not be true** because patients do not remember any details of the allergy [QI]*	“Penicillin Allergy Testing: Information for general practice” leaflet	• Provide information about the reasons of incorrect penicillin allergy labels	Beliefs about capability	Reflective motivation	Education	4.2 information about antecedents

*Perception of **importance of referral** and penicillin allergy becoming more on the radar [RR]*	"Penicillin Allergy Testing: Information for general practice" leaflet<br><br>Training	• Provide information of the benefit of PAT and appropriate allergy labels	Reinforcement<br><br>Beliefs about consequences<br><br>Memory<br><br>Environmental context and resources	Automatic motivation<br><br>Reflective motivation<br><br>Physical opportunities	Incentivisation<br><br>Education<br><br>Persuasion<br><br>Training	5.1 information about health consequences
***Previous medical** and reaction **history** of patients [QI]*	Consultation with patients	• Provide the opportunity to discuss with patients their medical history and why the test will be relevant for them	Beliefs about capabilities	Reflective motivation	Enablement	12.2 restructuring of social environment
*Awareness that it is **important to prescribe** the most suitable antibiotic [QI]*	"Penicillin Allergy Testing: Information for general practice" leaflet<br><br>Training	• Provide information about the benefit of taking penicillin and about antimicrobial resistance	Reinforcement<br><br>Memory	Automatic Motivation<br><br>Psychological capability	Incentivisation<br><br>Training	4.2 information about antecedents
*Belief that **testing would lead to removal** of the allergy label [RR]*	"Penicillin Allergy Testing: Information for general practice" leaflet	• Provide information about how to update the patients records after receiving a negative test results<br>• Provide information about prescribing penicillin after a negative test	Reinforcement	Automatic motivation	Incentivisation	1.1 goal setting<br><br>1.4 action planning.<br><br>4.1 instructions on how to perform a behaviour
*Belief of **benefits of removing** the allergy label [RR]*	"Penicillin Allergy Testing: Information for general practice" leaflet	• Provide information about how incorrect penicillin allergy labels can be harmful for patients	Reinforcement<br><br>Beliefs about consequences	Automatic motivation<br><br>Reflective motivation	Incentivisation<br><br>Education<br><br>Persuasion	5.1 information about health consequences

Previous experience of referring patients *to PAT [QI]*	Consultations with patients<br><br>PAT procedures in clinic	• Provide the opportunity to explain to patients about PAT procedures and results<br>• Provide the PAT service	Beliefs about capabilities	Reflective motivation	Enablement	15.1 verbal persuasion about capability
Warning patients *about side effects at the time of prescription [QI]*	Consultations with patients<br><br>“Penicillin allergy testing: going for a test” booklet	• Provide the opportunity to discuss any side effects with the patients	Social/Professional role and identity<br><br>Beliefs about capabilities	Social opportunities<br><br>Reflective motivation	Enablement	12.2 restructuring of social environment
NICE guidelines on importance of PAT *and criteria for selecting patients for referral [LR]*	“Penicillin Allergy Testing: Information for general practice” leaflet<br><br>Training	• Provide information on the NICE guidelines on referring to PAT	Knowledge<br><br>Social influences	Psychological Capability<br><br>Social opportunity	Training<br><br>Enablement	9.1 credible source
Belief that ***penicillin allergy is an important*** *issue [RR]*	“Penicillin Allergy Testing: Information for general practice” leaflet	• Provide information on incorrect allergy labels, adverse patient outcomes, effects on antimicrobial prescribing, and on antimicrobial resistance	Reinforcement	Automatic motivation	Education<br><br>Persuasion	5.1 information about health consequences
Perception of ***support of CCG*** *[QI]*	“Penicillin Allergy Testing: Information for general practice” leaflet	• Provide information about the support from colleagues and guidelines for referral	Professional Role	Reflective motivation	Persuasion	9.1 Credible source
Previous experience of removing *or challenging the records *QI]*	PAT procedures at clinic	• Provide the opportunity to refer patients to ultimately change inaccurate labels	Behavioural regulation	Psychological capability	Enablement	15.1 verbal persuasion of capability
**Target behaviour: Clinician prescription of penicillin after negative test result**						

Doubts on **accuracy of allergy labels** in medical records [QI]	"Penicillin Allergy Testing: Information for general practice" leaflet	• Provide information that the oral challenge is the gold standard test for penicillin allergy	Knowledge	Psychological capability	Education<br><br>Persuasion	4.2 information about antecedents
**Worries of harming patients** [QI, RR]	"Penicillin Allergy Testing: Information for general practice" leaflet	• Provide information about safety of patients having penicillin after the test	Emotions<br><br>Beliefs about consequences	Automatic motivation<br><br>Reflective motivation	Education<br><br>Persuasion	5.1 information about health consequences<br><br>5.6 information about emotional consequences
**Lack of knowledge on penicillin** [QI, RR]	"Penicillin Allergy Testing: Information for general practice" leaflet	• Provide information about penicillin allergy and effects on standard of care	Memory	Psychological capability	Training	2.7 feedback on outcomes of behaviour
**System did not distinguish between intolerances and allergies** and may still show alerts [QI]	Pop-up alert<br>WIs and allergy test results letter	• Provide information on the change of allergy status	Environmental context and resources<br><br>Beliefs about capabilities	Physical opportunity<br><br>Reflective motivation	Environmental restructuring<br><br>Enablement	12.5 adding objects to the environment
**Responsibility of changing the system** given to allergy clinic and lack of clarity on how to change them[QI]	Pop-up alert<br><br>Training, WIs and allergy test results letter	• Provide information on who will be responsible in changing the record	Social/professional role and identity<br><br>Beliefs about consequences<br><br>Behavioural regulation	Social opportunity<br><br>Reflective motivation<br><br>Psychological capability	Environmental restructuring<br><br>Enablement	4.1 instructions on how to perform a behaviour
**Lack of confidence** that penicillin could be safely administered [RR]	"Penicillin Allergy Testing: Information for	• Provide information about safety of taking penicillin when the test result is negative	Beliefs about consequences	Reflective motivation	Education persuasion	5.1 beliefs about health consequences

	general practice" leaflet					
**Lack of guidance** on how to interpret the results [QI]	"Penicillin Allergy Testing: Information for general practice" leaflet<br><br>Training, WIs, allergy test results letter	• Provide information on how the results will be presented to the clinicians and patients	Knowledge<br><br>Skills	Psychological capability<br><br>Cognitive skills	Education<br><br>Persuasion<br><br>Training	4.1 instructions on how to perform a behaviour<br><br>2.7 feedback on outcome of behaviour
Perception that **anxious patients may still not want to take penicillin,** and they would believe other people's advice instead [QI]	Consultation with patients<br><br>"Penicillin allergy testing: going for a test" booklet	• Provide the opportunity to discuss the accuracy of the results with patients	Beliefs about capability<br><br>Reinforcement<br><br>Social influences<br><br>Behavioural regulation	Reflective motivation<br><br>Automatic motivation<br><br>Social opportunity<br><br>Psychological capability	Enablement<br><br>Incentivisation	12.2 restructuring of social environment
**Lack of follow** up of the results [QI]	Pop-up alert Allergy test results letter	• Provide an alert when the results are available and the indication of a change of allergy label	Beliefs about consequences<br><br>Behavioural regulation	Reflective motivation<br><br>Psychological capability	Enablement<br><br>Environmental restructuring	12.4 adding object to the environment
Awareness **of different approaches in changing records** in different practices [QI]	Training , WIs, allergy test results letter	• Provide information of the new system in changing labels after PAT and how practices have been involved	Environmental context and resources<br><br>Behavioural regulation	Physical opportunity<br><br>Psychological capability	Environmental restructuring<br><br>Modelling	12.2 restructuring of the social environment<br><br>3.2 social support (practical)

Patients may worry less *about allergies [QI]*	Consultation with patients	• Provide the opportunity to reassure patients	Social influences	Social opportunity	Environmental restructuring	12.2 restructuring of social environment
Recognition that ***test provided evidence*** *that patient is not allergic [QI]*	"Penicillin Allergy Testing: Information for general practice" leaflet<br><br>PAT procedures at clinic	• Provide information about the accuracy of the test and who will perform it	Belief about consequences	Reflective motivation	Education<br><br>Persuasion	5.1 information about health consequences
Awareness that the ***test will allow prescribing first line*** *antibiotics and fight antibiotics resistance [QI]*	"Penicillin Allergy Testing: Information for general practice" leaflet	• Provide information about the effects of accurate penicillin allergy labels	Reinforcement	Automatic motivation	Incentivisation	2.7 feedback on outcomes of behaviour
Perception of ***penicillin allergy testing to be specialist advice*** *and to be objective evidence[QI]*	"Penicillin Allergy Testing: Information for general practice" leaflet	• Provide information of who will perform the test and interpret the results	Belief about consequences<br><br>Reinforcement	Reflective motivation<br><br>Automatic motivation	Education<br><br>Persuasion	5.1 information about consequences
Discussion of the negative test results *to address patients concerns about taking penicillin [QI]*	Consultations with patients	• Provide opportunity to discuss results with patients	Beliefs about capabilities<br><br>Social influences	Reflective motivation<br><br>Social opportunity	Enablement	5.1 beliefs about emotional consequences
Previous experience *of removing or challenging the records [QI]*	WIs, allergy test results letter	• Provide information about how to remove labels	Skills	Cognitive skills	Training	4.1 instructions on how to perform a behaviour
Perception that ***the system is easy to change*** *[QI]*	Pop-up alert, WIs and allergy test results letter	• Provide information about the change of label in the system	Beliefs about capabilities	Reflective motivation	Enablement<br><br>Environmental restructuring	12.5 adding objects to the environment

			Environmental context and resources	Physical opportunity		
Intention to change the label *[QI]*	Training, allergy test results letter	• Provide support and instructions on changing the label	Reinforcement	Automatic motivation	Incentive	2.7 feedback on outcomes of behaviour

Table S1b: Behavioural Analysis Table for the AlABAMA intervention, patient behaviours

**Barriers / *facilitators* to target behaviours**	**Intervention Component/s**	**Intervention ingredient**	**Theoretical Domains Framework (TDF)**	**Target construct (BCW)**	**Intervention function (BCW)**	**Behaviour Change Technique (using 93 BCT taxonomy v1)**
**Patient attendance at Penicillin Allergy Testing (PAT)**						
**Lack of information around the benefits of having access to penicillin**, when patients didn't experience any negative consequences of the allergy label [QI]	"Penicillin allergy testing: going for a test" booklet<br><br>Trial PIS	• Provide evidence on why penicillin is the preferred and best treatment for infections and kills fewer good bacteria;<br>• Provide information that other antibiotics could be less effective, and could increase the risk of "superbug" infections	Beliefs about consequences	Reflective Motivation	Education<br><br>Persuasion	5.1 information about health consequences
**Difficulty for patients to have access to the right antibiotics** if there is a penicillin allergy [QI]	PAT procedures at allergy clinic	• Provide opportunity to undertake allergy testing	Reinforcement	Automatic motivation	Environmental restructuring	12.2 restructuring of physical environment
Concerns related **to attending** the appointment [QI]	Consultations with clinicians;<br><br>Trial PIS	• Provide information about how the appointment will be arranged and that some travel expenses will be provided	Environmental Context and resources	Physical opportunity	Enablement	1.4 action planning

**Barriers / *facilitators* to target behaviours**	**Intervention Component/s**	**Intervention ingredient**	**Theoretical Domains Framework (TDF)**	**Target construct (BCW)**	**Intervention function (BCW)**	**Behaviour Change Technique (using 93 BCT taxonomy v1)**
Concerns whether the **GP will encourage/support** patient to have the test [QI]	Consultations with clinicians<br><br>Trial PIS	• Provide the opportunity to discuss the benefits of the testing with their clinicians	Social influences	Social opportunity	Enablement	3.1 social support (unspecified)
**Unsure about what the test is going to involve** and uncertainty around doses of penicillin given during the test [QI]	"Penicillin allergy testing: going for a test" booklet<br><br>Trial PIS	• Provide information about what a penicillin allergy test would involve, explaining the three stages of the testing	Knowledge;<br><br>Beliefs about consequences	Psychological capability<br><br>Reflective Motivation	Education	2.7 feedback on outcomes of behaviour<br><br>5.1 information about health consequences
**Lack of information about** the **credibility** of PAT [*QI]	Consultations with clinicians	• Provide the opportunity to address any concerns about what PAT is and what other options they may have	Knowledge;<br><br>Beliefs about consequences	Psychological capability<br><br>Reflective Motivation	Education	2.7 feedback on outcomes of behaviour<br><br>5.1 information about health consequences
**Concerns that the procedure is not safe** [QI]	"Penicillin allergy testing: going for a test" booklet<br><br>Trial PIS	• Provide information on the common reactions to PAT during the clinic visit and how patients will be treated by medical staff if they experience any reaction	Beliefs about consequences	Reflective Motivation	Persuasion	5.1 information about health consequences
**Concerns about a possible reaction** during test if they have been told by their clinicians for many years to avoid penicillin [QI]	"Penicillin allergy testing: going for a test" booklet	• Provide reassurance that the allergy test is very safe and that serious reactions are very rare.	Emotions	Automatic motivation	Persuasion	5.1 information about emotional consequences

**Barriers / *facilitators* to target behaviours**	**Intervention Component/s**	**Intervention ingredient**	**Theoretical Domains Framework (TDF)**	**Target construct (BCW)**	**Intervention function (BCW)**	**Behaviour Change Technique (using 93 BCT taxonomy v1)**
**Apprehension about having a test** for patients with a previous severe allergic reaction [QI]	"Penicillin allergy testing: going for a test" booklet<br><br>Trial PIS	• Provide reassurance that the test will be offered only if the nurses and doctors in the allergy clinic think that the patient is low risk	Emotions	Automatic motivation	Persuasion	5.1 information about emotional consequences
Concerns about a reaction that could **worsen their overall state of health** when patients have a chronic co-morbidity [QI]	Consultations with clinicians	• Provide reassurance about the safety of test	Beliefs about consequences;<br><br>Emotions	Reflective Motivation<br><br>Automatic motivation	Education<br><br>Persuasion	5.1 information about health consequences<br><br>5.6 information about emotional consequences
Perception of **skin testing as less frightening** than the oral challenge test [QI]	"Penicillin allergy testing: going for a test" booklet	• Provide information about the oral challenge test; Provide reassurance that the length of time spent at the clinic is long enough to check for any delayed reactions	Beliefs about consequences;<br><br>Emotions	Reflective Motivation<br><br>Automatic motivation	Education<br><br>Persuasion	2.7 feedback on outcomes of behaviour<br><br>5.1 information about health consequences
Patients **concerned about how they would be monitored** during the allergy test and whether doctors would respond quickly in case of reaction [QI]	"Penicillin allergy testing: going for a test" booklet<br><br>Trial PIS	• Provide information on how doctors and nurses will monitor patients during the skin testing and the oral challenge	Beliefs about consequences;<br><br>Emotions	Reflective Motivation<br><br>Automatic motivation	Education<br><br>Persuasion	5.1 information about health consequences

**Barriers / *facilitators* to target behaviours**	**Intervention Component/s**	**Intervention ingredient**	**Theoretical Domains Framework (TDF)**	**Target construct (BCW)**	**Intervention function (BCW)**	**Behaviour Change Technique (using 93 BCT taxonomy v1)**
**Worries about taking penicillin at home** following the allergy test in clinic and whether there is someone to call in case of reaction [QI]	"Penicillin allergy testing: going for a test" booklet	• Provide information on how the nurse will contact the patients after they have finished taking the penicillin at home to check whether they had any symptoms;<br>• Provide information of possible side effects when taking penicillin at home and that they are mild and do not require treatment<br>• Provide contact details of who to call at the clinic once they experience side effects at home	Beliefs about consequences<br><br>Emotions	Reflective Motivation<br><br>Automatic motivation	Education<br><br>Persuasion	5.1 information about health consequences
Perception that the procedure is **over medicalised** [QI]	"Penicillin allergy testing: going for a test" booklet<br><br>PAT procedures at allergy clinic	• Provide information on the test procedures and on how only the necessary tests will be taken: some of the stages will be skipped based on previous history of reactions and on reaction to the skin test (only necessary tests are carried out)	Beliefs about consequences<br><br>Knowledge	Reflective Motivation	Education<br><br>Persuasion	5.1 information about health consequences
**Lack of knowledge** that allergy status could change over time [RR]	"Penicillin allergy testing: going for a test" booklet	• Provide information about consequences of incorrect allergy label and the benefit of taking the test	Knowledge	Psychological capability	Education	4.2 information about antecedents
Primary care providers **did not inform them about the availability** of PAT [RR]	Consultations with clinicians<br><br>Invitation letter/Trial PIS	• Provide information about penicillin allergy labels and PAT	Knowledge	Psychological capability	Incentivisation<br><br>Education	2.7 feedback on outcome of behaviour

**Barriers / *facilitators* to target behaviours**	**Intervention Component/s**	**Intervention ingredient**	**Theoretical Domains Framework (TDF)**	**Target construct (BCW)**	**Intervention function (BCW)**	**Behaviour Change Technique (using 93 BCT taxonomy v1)**
Always having had a label of penicillin allergy [QI]	"Penicillin allergy testing: going for a test" booklet	• Provide information that once the allergy label is on the patient's medical record is rarely questioned and difficult to remove<br>• Provide information about the percentages of patients who really have a penicillin allergy and the reasons of incorrect allergy label.	Behavioural regulation<br><br>Beliefs about capabilities	Psychological capability<br><br>Reflective Motivation	Education<br><br>Persuasion	5.1 beliefs about health consequences
*Previous **experience of negative consequences** of having a penicillin allergy label and intentions to take penicillin [QI]*	Consultations with clinicians	• Provide an opportunity for patients to discuss with clinicians their previous medical history of penicillin allergy reactions and highlight their personal motivations to undertake the test	Emotion	Automatic motivation	Incentivisation	2.7 feedback on outcomes of behaviour
*Patient **awareness that penicillin is a first-line treatment** for many infections [QI]*	"Penicillin allergy testing: going for a test" booklet<br><br>Trial PIS	• Provide evidence on benefits of taking penicillin compared to broad spectrum antibiotics	Knowledge<br><br>Beliefs about consequences	Psychological capability<br><br>Reflective Motivation	Education	2.7 feedback on outcome of behaviour
*Feeling **relieved because allergy status has been confirmed** [QI]*	"Penicillin allergy testing: going for a test" booklet	• Provide information about the accuracy of allergy labels	Reinforcement	Automatic motivation	Incentivisation	2.7 feedback on outcome of behaviour
*Diagnosis is **an emotionally difficult event** and relief in getting an answer [QI]*	Consultations with clinicians<br><br>"Penicillin allergy testing: a negative test result" booklet	• Provide information on how they will get the results of the testing and provide opportunity to discuss it with GPs<br>• Provide information on the meaning on a negative test, who to inform about the test result and how to do so	Emotions	Automatic motivation	Persuasion	5.3 information about social and environmental consequences

**Barriers / *facilitators* to target behaviours**	**Intervention Component/s**	**Intervention ingredient**	**Theoretical Domains Framework (TDF)**	**Target construct (BCW)**	**Intervention function (BCW)**	**Behaviour Change Technique (using 93 BCT taxonomy v1)**
Assurance of ***access to trained medical staff*** *at the time of the test [QI]*	"Penicillin allergy testing: going for a test" booklet	• Provide reassurance that all medical staff are fully trained and will act immediately in case of allergy reaction	Beliefs about consequences;<br><br>Emotions	Reflective Motivation<br><br>Automatic motivation	Education<br><br>Persuasion	5.1 information about health consequences
Perception of being ***properly monitored*** *and of feeling safe during the test [QI]*	"Penicillin allergy testing: going for a test" booklet<br><br>PAT procedures at allergy clinic<br><br>Trial PIS	• Provide reassurance that the medical staff will monitor the patients during the test and will follow up after the patients will have finished the course at home	Beliefs about consequences<br><br>Emotions	Reflective Motivation<br><br>Automatic motivation	Education<br><br>Persuasion	5.3 information about social and environmental consequences
Feeling ***safe if the test takes place on hospital premises*** *[QI]*	"Penicillin allergy testing: going for a test" booklet<br><br>PAT procedures at allergy clinic<br><br>Trial PIS	• Provide information on where the allergy clinic is.<br>• Attending the PAT at the allergy clinic at the hospital	Knowledge	Psychological capability	Education	2.7 feedback on outcomes of behaviour
Clear ***expectations of what the procedure involves?*** *[QI]*	Consultations with clinicians;<br><br>"Penicillin allergy testing: going for a test" booklet<br><br>Trial PIS	• Provide information about the procedures of skin testing and oral challenge test	Knowledge	Psychological capability	Education	2.7 feedback on outcomes of behaviour<br><br>4.2 information about antecedents

**Barriers / *facilitators* to target behaviours**	**Intervention Component/s**	**Intervention ingredient**	**Theoretical Domains Framework (TDF)**	**Target construct (BCW)**	**Intervention function (BCW)**	**Behaviour Change Technique (using 93 BCT taxonomy v1)**
*Opportunity for **addressing any concerns** about the procedure [QI]*	Consultations with clinicians	• Provide the opportunity to discuss any concerns about taking the test	Environmental Context and resources;<br><br>Emotions	Physical opportunity<br><br>Automatic motivation	Enablement	12.2 restructuring of social environment
*Belief that PAT provides **valuable information** [RR]*	PAT procedures at allergy clinic;<br><br>"Penicillin allergy testing: a negative test result" booklet	• Provide information about penicillin allergy label<br>• Provide information about the meaning of negative test result and the safety of taking penicillin	Beliefs about consequences	Reflective motivation	Incentivisation	2.7 feedback on outcomes of behaviour
***Interest** in being tested [RR]*	PAT procedures at allergy clinic<br><br>"Penicillin allergy testing: going for a test" booklet	• Provide information about the benefit of taking the test	Reinforcement	Automatic motivation	Incentivisation	2.7 feedback on outcomes of behaviour
***Benefits** of the negative test results [QI]*	"Penicillin allergy testing: going for a test" booklet	• Provide information about the benefits of having a negative test results (being able to take penicillin, being less worries about penicillin, not having to go back to the GP with a reaction)	Reinforcement	Automatic motivation	Incentivisation	2.7 feedback on outcome of behaviour
***Importance of knowing** that the penicillin allergy is correct and that the penicillin allergy test is positive [*QI]*	Consultations with clinicians;<br><br>Invitation letter/Trial PIS	• Provide information about the benefits of accurate allergy labels	Reinforcement	Automatic motivation	Incentivisation	2.7 feedback on outcome of behaviour

Barriers / *facilitators* to target behaviours	Intervention Component/s	Intervention ingredient	Theoretical Domains Framework (TDF)	Target construct (BCW)	Intervention function (BCW)	Behaviour Change Technique (using 93 BCT taxonomy v1)
**Target behaviour: Patient consumption of penicillin when prescribed first line**						
**Perceived risk of clinicians' not prescribing** penicillin even after a negative result [QI]	"Penicillin allergy testing: a negative test result" booklet<br><br>Post-test intervention card	• Provide a card which will inform the clinicians of the change in allergy label<br>• Provide information on how the GP will be informed of the allergy test result	Social Influences	Social opportunities	Enablement	3.2 social support (practical)<br><br>12.2 restructuring of social environment
**Perceived risk of Re-labelling** of patients allergy even after negative test result [QI]	"Penicillin allergy testing: a negative test result" booklet<br><br>Post-test intervention card,<br><br>allergy test result letter	Provide information on how the GP will update the medical record of the patient and other medical systems with negative test results	Social influences	Social opportunities	Enablement	3.2 social support (practical)
Perception of test as **not accurate** [QI, RR]	"Penicillin allergy testing: a negative test result" booklet	Provide information that the test was very accurate in identifying whether the patients have a true penicillin allergy	Beliefs about consequences	Reflective motivation	Education<br><br>Persuasion	5.1 information about health consequences
**Severity** of past allergy reactions [QI]	PAT procedures at allergy clinic	Provide information that nurse or doctors at allergy clinic will only test patient who are perceived as low risk	Beliefs about consequences<br><br>Knowledge	Reflective motivation<br><br>Psychological capability	Education<br><br>Persuasion	4.3 information about antecedents

**Barriers / *facilitators* to target behaviours**	**Intervention Component/s**	**Intervention ingredient**	**Theoretical Domains Framework (TDF)**	**Target construct (BCW)**	**Intervention function (BCW)**	**Behaviour Change Technique (using 93 BCT taxonomy v1)**
**Patient Belief that they are still allergic** to penicillin after a negative text result [RR]	"Penicillin allergy testing: a negative test result" booklet	Provide information about the reliability of the test	Beliefs about consequences	Reflective motivation	Education<br><br>Persuasion	5.1 beliefs about consequences
**Fear of having an allergic reaction** again [RR]	"Penicillin allergy testing: a negative test result" booklet	Provide information about low risks of having an allergic reaction in the future	Emotion	Automatic Motivation	Education<br><br>Persuasion	5.6 beliefs about emotional consequences
**Lack of confidence** that penicillin could be safely administered [RR]	"Penicillin allergy testing: a negative test result" booklet	Provide information about the meaning of test results and which antibiotics are now safe to use	Beliefs about consequences	Reflective motivation	Education<br><br>Persuasion	5.1 beliefs about consequences
**Lack of knowledge of which antibiotics they could take** safely [RR]	"Penicillin allergy testing: a negative test result" booklet	Provide a list of antibiotics that patients with a negative test result can take	Knowledge	Psychological capability	Education	4.2 information about antecedents
**Distrust in health care providers'** advice if they don't prescribe penicillin even after the negative test result [RR]	Post-test intervention card	Provide card to show to health care professionals which gives details about the allergy test completed and explains that the patient had a negative test result	Reinforcement	Automatic motivation	Environmental restructuring	12.2 restructuring of social environment<br><br>12.5 adding objects to the environment
**Lack of knowledge about how to interpret skin test results** [RR]	Post-test intervention card	Provide explanation of the meaning of negative test results and what to do the next time they will need antibiotics	Knowledge	Psychological capability	Education	4.2 information about antecedents
**No differences in concerns** between patients who were investigated through skin test or oral test [RR]	"Penicillin allergy testing: a negative test result" booklet	Provide information about the accuracy of the test completed	Beliefs about consequences	Reflective motivation	Education<br><br>Persuasion	5.1 beliefs about consequences

**Barriers / *facilitators* to target behaviours**	**Intervention Component/s**	**Intervention ingredient**	**Theoretical Domains Framework (TDF)**	**Target construct (BCW)**	**Intervention function (BCW)**	**Behaviour Change Technique (using 93 BCT taxonomy v1)**
*Perception of the test as **definitive answer** and proof of allergy status [QI]*	PAT procedures at allergy clinic;<br><br>"Penicillin allergy testing: a negative test result" booklet<br><br>Post-test intervention card<br><br>allergy test result letter	• Provide the test results from the allergy clinic<br>• Provide information on the meaning of the negative test result and information about the accuracy of the test<br>Provide information about reliability of test	Reinforcement	Automatic motivation	Environmental restructuring	2.7 feedback on outcomes of behaviour
*Perception to have undergone a **thorough testing procedure** [QI]*	PAT procedures at allergy clinic	Monitoring of the patients by the nurse or doctor at allergy clinic who will provide medical assistance when needed	Reinforcement	Automatic motivation	Environmental restructuring	12.2 restructuring of the social environment
***Lack of reaction** following the test [QI]*	PAT procedures at allergy clinic<br><br>"Penicillin allergy testing: going for a test" booklet	Provide information about the type of reactions and their indication on the state of the allergy	Reinforcement	Automatic motivation	Environmental restructuring	12.1 restructuring of the physical environment

S2. Think-aloud topic guides for clinicians and patients

"Penicillin Allergy Testing: Information for general practice" leaflet

In this feedback session, I am interested in what you think about when you read the information provided in the booklets. I would like you to think aloud as you read through each section, if you have any comments. There is no right or wrong answers. So when you read a section of the booklet, if you could tell me what thoughts come to your mind when you read it.

1. Looking at the Penicillin allergy" and "Incorrect penicillin allergy records are very common what did you think after reading it? Did you think it was comprehensive/ new information/ useful information
2. Going through the section on "Incorrect penicillin allergy records can be harmful for patients what did you think while reading it? What did this section tell you? Did you find it convincing?
3. Looking at the section "Penicillin allergy testing -What does the test involve?", was it clear to you what the testing involves? Would you feel confident in explaining the process to patients?
4. What do you think about the section: "How accurate is the test"? How do you understand this section? Is there anything unclear?
5. What do you think about the section: "Are there any risks to patients having the test?" Do you feel reassured by this section? Would you feel confident to refer patients for testing based on this information?
6. What do you think about the section: "How should I discuss the test with patients? "? Do you think this is an important section? Is the description clear?
7. What do you think about the section: "Updating medical records"? Is it clear what you need to do?
8. What do you think about the section: "Prescribing penicillin after a negative test"? Would you feel confident to prescribe penicillin after reading this section?

- What are your overall thoughts on the leaflet?
- How can this leaflet be improved?
- What will stick in your mind from this leaflet?
- How easy or difficult was it to read/ understand this leaflet?
- Is there anything which you think is missing?
-

Intervention Card

- What do you think about this card?

- Can you tell me how do you understand the last sentences: that the test was negative which means that the patient is not allergic
- What does: with the same risk of allergy as the general population mean to you?

Can you tell me what you would do if a patient came with this card

"Penicillin allergy testing: going for a test" booklet

In this feedback session, I am interested in what you think about when you read the information provided in the booklets. I would like you to think aloud as you read through each section, if you have any comments. There is no right or wrong answers. So when you read a section of the booklet, if you could tell me what thoughts come to your mind when you read it.

9. Looking at the front cover the writing and the pictures, what did you think this leaflet was going to be about?
10. Looking at page 3, with a list of contents, what did you think after reading it? Did you at that point think that it was comprehensive?
11. Going through the first section: "How do I know if I am allergic to penicillin", what did you think while reading it? What did this section tell you? Why do you think this section is here?
    Specifically, there is some numerical information provided here about how many people are allergic etc, can you tell me in your own words what you understand by this paragraph?
12. Going through the next paragraph on why is it important for people to have a penicillin allergy test, again what did you think while reading it? Were you convinced by the argument? How do you understand this section?
13. Going through the next section: "what does a penicillin allergy test involve", what do you think of this section? Could you talk me through what you think you would have to do if you had this test? Is there anything unclear? Is there anything missing? What do you think about the test and what is required of you.
14. Going through the next section: "Are there any risks", what was going through your head when you were reading that? What were you feeling when you have read that? How would you feel about going for a test after reading this section? Specifically, this section tell you what symptoms people may get- what did you think while reading this section? What is this section telling you? How would you put this section in your own words?

- What are your overall thoughts on the leaflet?
- How can this leaflet be improved?
- What will stick in your mind from this leaflet?

- How easy or difficult was it to read/ understand this leaflet?

"Penicillin allergy testing: a negative test result" booklet

1. Looking at the front cover the writing and the pictures, what did you think this leaflet was going to be about? How do you understand the heading: Negative test result? What did you think about the picture?
2. Looking at the table of contents, what did you think after reading it? Did you at that point think that it was comprehensive?
3. Going through the next section: "what does a negative test result mean", what was going through your head when you were reading that? How do you understand this section? What do you think about the picture used here?
4. Going through the next section: "Is it safe for me to now take penicillin", what were you thinking while going through this section?
5. Going through the next section: "What should I do now?" How do you understand this section? What were you thinking reading this section? Can you tell me in your words what you are asked to do?
6. Going through the next section: "What should I do next time I have an infection?", how do you understand this section?

- What are your overall thoughts on the leaflet?
- How can this leaflet be improved?
- What will stick in your mind from this leaflet?
- How easy or difficult was it to read/ understand this leaflet?

Intervention Card

- What do you think about this card?
- Can you tell me how do you understand the last sentences: that the test was negative which means that the patient is not allergic
- What does: with the same risk of allergy as the general population mean to you?
- Can you tell me in what situations you think you may use it?

S3. Table of changes: "Penicillin Allergy Testing: going for a test" booklet

Suggested changes	Changes made	Reasons for change	Priority for change
**Front page**			
The image on the cover of the booklet must be changed as it does not relate to what happens during penicillin allergy testing; also the blood samples on the GP desk look scary.	A new image was selected showing a more friendly looking consultation between patient and GP without any medical equipment	EO	Must do
The logos could be put at the bottom of the cover pager (rather than at the top) as they distract from the image)	The logos are on the bottom of the page	EO	Could do
The participants suggested making the booklets even easier to follow by adding text boxes throughout to separate big chunks of text.	The booklet was made more visually appealing	EO	Could do
**How can this booklet help me?**			
The contents page looks a bit dense on the page. The participants did not like the "chemical symbols" image in the background design.	• Better use of the space on the contents page was made and the text more was spread across the page.<br>• The introduction to the booklet was put in a separate box to make it look more manageable. Chemical symbols image were removed.	EO	Should do

Suggested changes	Changes made	Reasons for change	Priority for change
**How do I know if I am allergic to penicillin?**			
The participants suggested putting the last sentence of this section: “A penicillin allergy test is the safest way to find out whether or not you are allergic to penicillin” in bold.	The last sentence was put in bold	EO, QI	Must do
Statistics about the prevalence of allergy were not always understood by the participants, as the participants often thought that 1 in 10 people are allergic. They wanted a more visual presentation of this key information	An infographic was created showing how many people think they are allergic and how many people actually are allergic. The image of a GP taking blood pressure was replaced by the infographic.	EO	Should do
This section looks also a bit dense on the page	The text was spread across to the following page	EO	Should do
**Why is it important for people to have a penicillin allergy test?**			
Participants were unsure what narrow and broad spectrum antibiotics were and did not recognise MRSA abbreviation.	• The reference to narrow and broad spectrum antibiotics was removed<br>• The full name for MRSA was presented	RT, CT	Must do
Participants did not always know that penicillin is more than one antibiotic. This message should be made clearer.	An additional sentence was added to provide information that penicillin includes a number of different antibiotics	EO, QI	Must do

Suggested changes	Changes made	Reasons for change	Priority for change
Booklet 2 mentioned that penicillin is good at treating throat infections- this information was missing from this booklet.	A sentence was added to explain that "antibiotics are very useful to treat many infections, including ear, skin, throat and chest infections"	EO, QI	Must do
The information was slightly repetitive and perhaps the section should start with the strongest message (which participants thought was that penicillin are effective).	• The section was made more concise.<br>• The first paragraph includes information on what penicillins are, what they are used for, and that they are considered the best treatment for infections	EO	Should do
Other sentences in this section, for example: "other antibiotics may not be as effective" could also be put in bold.	The sentence was put in bold	EO, QI	Should do
The section looks a bit dense.	The text was spread across the previous page	EO	Should do
**What does a penicillin allergy test involve?**			
Participants wanted to know if they can call the allergy clinic and whether they will be provided a number and what details the doctor or nurse will be checking (Why they would not have to be checked in person?).	It reads: "you will be provided with details of who to call if you have any symptoms or concerns"	RT, CT, QI	Must do

Suggested changes	Changes made	Reasons for change	Priority for change
Participants wanted to have reassurance that 3 days would be enough to detect delayed reactions.	It reads: “this is long enough to check for any delayed reactions”	RT, CT, QI	Must do
Participants suggested making it clearer that the clinic is in the hospital.	This sections starts with “Penicillin allergy testing takes place in a hospital, in a specialist allergy clinic”. The sentence was put in bold	RT, CT, QI	Must do
Participants had queries about symptoms they could experience after three days of taking penicillin as the booklet said: “mild and mostly side effects”; they wanted clarification whether these could be allergic reaction symptoms	It reads: “these symptoms are generally mild and do not require treatment”	RT, CT, QI	Must do
Participants did not always like the phrase “like a mosquito bite” (skin testing stage) as some people may have an “unpleasant reaction to mosquitos”.	No change made – comment from one participant, felt by clinical team to be best way to describe and usual term used in current NHS leaflets.	RT, CT	Could do
There is a lot of text on one page	The descriptions of the test was spread over two pages	EO	Should do
A diagram alongside the stages of the test could be presented to make it even clearer	A diagram was provided for the three stages of testing and the description of the test was spread over two pages	EO	Could do
**Are there any risks to having the test?**			

Suggested changes	Changes made	Reasons for change	Priority for change
The participants wanted to have a separate paragraph on what can happen during the test and what can happen during 3 days of taking penicillin at home.	There are two paragraphs, one on what could happen during the clinic visit, and one about what could happen at home	RT, CT	Must do
They wanted clarification whether the listed symptoms for taking penicillin at home: "sickness, a mild, small rash, nausea, upset stomach, or other mild symptoms" were symptoms of an allergic reaction; this was especially confusing as feeling sick was also listed as part of the allergic reaction when taking penicillin in the clinic (you might feel sick, itchy, develop hives ('nettle rash'), swelling, a fast heartbeat, dizziness or difficulty breathing.	It reads: "Once you go home, it is possible you may develop mild symptoms such as sickness, a mild, small rash, nausea, upset stomach, or other mild symptoms. It is extremely unlikely that you will have any serious symptoms, as these would have happened when you took penicillin during the clinic visit"	RT, CT, QI	Must do
The participants suggested putting the phrase: "extremely unlikely" in bold ("It is extremely unlikely that you will have any serious symptoms, as these would have happened when you took penicillin during the clinic visit".	The phrase was put in bold	EO	Could do

Note: EO: patient; RT: research team; CT: clinical team; QI: qualitative interviews

S4. Full description of the AlABAMA (ALlergy AntiBiotics And Microbial resistAnce) Intervention package in accordance with the Template for Intervention Description and Replication (TIDieR) framework.

- **1. BRIEF NAME:** AlABAMA (ALlergy AntiBiotics And Microbial resistAnce) Intervention package
- **2. WHY:** Penicillins are the most prescribed antibiotics and they are used as first-line therapy for many infections. Side effects and symptoms related to the infections are commonly mislabelled as allergy. Around 6% of people think that they are allergic to penicillin but fewer than 10% of these patients are truly allergic. As a consequence a significant proportion of the population have restricted access to highly effective penicillins, and are prescribed broad-spectrum antibiotics instead, which may be less effective and could increase the risks of acquiring multi-drug resistant bacteria. The goal of AlABAMA is to introduce a "pre-emptive" penicillin allergy assessment pathway (PAAP) for patients who are more likely to receive antibiotics that could impact antibiotic prescribing, have patients benefits, limit antimicrobial resistance (AMR) infections and deliver NHS cost savings. The AlABAMA approach will develop a "one stop shop" single Penicillin Allergy Test (PAT) at the hospital clinic for specialist immunology assessment (skin testing and/or oral challenge test).
- **3&4: WHAT:** AlABAMA is a complex behaviour change intervention which targets prescribers in general practice and low risk patients with a penicillin allergy record. See Table 2 for an overview of the key intervention components. For more details about the specific behaviour change techniques used, refer to the behavioural analysis.
- **5. WHO PROVIDED**: The AlABAMA research team will provide intervention materials to general practices taking part in the feasibility and main trial. Practices will be responsible for local implementation by the practice manager(s). GPs will have consultations with patients to check eligibility for entering the trial.
  Research nurses at the hospital will be responsible for sending the two intervention booklets to patients in the intervention arm by post. Research nurses at the immunology hospital clinic will deliver Stage 1, Stage 2 and/or stage 3 of the PAT.

- **6. HOW**: The intervention will be delivered mainly by written information for prescribers and for patients in the intervention arm. The site training will be delivered to all the GP leads and the practice manager(s) in one session per practice. Prescribers will receive information by email. Patients randomised to the PAAP intervention arm will receive the "Penicillin Allergy Testing: going for a test" by post. Patients will complete Stage 1 and Stage 2 (skin test) and /or Stage 3 (oral challenge test) in a single hospital visit which lasts around 3 hours. Patients with a negative PAT result will receive the "Penicillin Allergy Testing: a Negative Test Result" booklet by post. Prescribers will receive the update about the penicillin allergy test result by post and electronically.
- **7. WHERE**: AlABAMA is intended for implementation in general practices, where the site training and patient consultation also take place. The PAT takes place at the immunology clinic of a local hospital. Written information for clinicians is provided electronically subsequently to the site training. Written information for patients is sent by post by research nurses at the hospital.
- **8. WHEN and HOW MUCH**: Site training is provided by the AlABAMA research team prior to the screening and eligibility assessment of patients. Patients randomised to the PAAP trial arm will be sent the "Penicillin Allergy Testing: going for a test" booklet with their hospital appointment letter. Patients randomised to the control arm will receive usual clinical care. All patients completing the PAT will receive a letter with the test result after their appointment. Patients who have tested negative will receive the "Penicillin Allergy Testing: a Negative Test Result" booklet and Intervention card by post. Practices will be informed of the test result and instructed to update the patients' electronic health record.
- **9. TAILORING**: The implementation of behavioural and clinical elements of the AlABAMA intervention package will be implemented in all GP practices and local immunology clinics participating in the trial.
- **10. MODIFICATIONS**: Modifications were made to the intervention materials following qualitative feedback from the feasibility study.
- **11-12. HOW WELL**: The intervention is being tested in a feasibility trial in 8 general practices. It will then be tested in a randomised control trial across 70 GP practices.

S5 GUIDED – a guideline for reporting for intervention development studies.

Item description	Page in manuscript where item is located
1. Report the context for which the intervention was developed.	4
2. Report the purpose of the intervention development process.	5
3. Report the target population for the Intervention development process.	4
4. Report how any published intervention development approach contributed to the development process	5
5. Report how evidence from different sources informed the intervention development process.	6
6. Report how/if published theory informed the intervention development process.	11
7. Report any use of components from an existing intervention in the current intervention development process.	5
8. Report any guiding principles, people or factors that were prioritised when making decisions during the intervention development process.	9
9. Report how stakeholders contributed to the Intervention development process.	8
10. Report how the intervention changed in content and format from the start of the intervention development process.	14-15
11. Report any changes to interventions required or likely to be required for subgroups.	15-17
12. Report important uncertainties at the end of the intervention development process.	21-22

13. Follow TIDieR guidance when describing the developed intervention.	18
14. Report the intervention development process in an open access format.	1
