# Supplementary material for: Developing a behavioural intervention package to identify and amend incorrect penicillin allergy records in UK general practice and subsequently change antibiotic use
Source: BMJ Open. 2020 Oct 1;10(10):e035793. doi: 10.1136/bmjopen-2019-035793 (PMC7534681; doi:10.1136/bmjopen-2019-035793)
Supplement: Author's manuscript [file bmjopen-2019-035793.draft_revisions.pdf]

BMJ Open is committed to open peer review. As part of this commitment we make the peer review history of every article we publish publicly available.

When an article is published we post the peer reviewers' comments and the authors' responses online. We also post the versions of the paper that were used during peer review. These are the versions that the peer review comments apply to.

The versions of the paper that follow are the versions that were submitted during the peer review process. They are not the versions of record or the final published versions. They should not be cited or distributed as the published version of this manuscript.

BMJ Open is an open access journal and the full, final, typeset and author-corrected version of record of the manuscript is available on our site with no access controls, subscription charges or pay-per-view fees (http://bmjopen.bmj.com).

If you have any questions on BMJ Open's open peer review process please email info.bmjopen@bmj.com

BMJ Open

Developing a behavioural intervention package to identify and amend incorrect penicillin allergy records in UK general practice

Journal:	*BMJ Open*
Manuscript ID	bmjopen-2019-035793
Article Type:	Original research
Date Submitted by the Author:	18-Nov-2019

Complete List of Authors:	Santillo, Marta; Oxford University, Nuffield Department of Primary Care Health Sciences<br>Wanat, Marta; Oxford University, Nuffield Department of Primary Care Health Sciences<br>Davoudianfar, Mina; University of Oxford, Clinical Trials Unit, Nuffield Department of Primary Care Health Sciences<br>Bongard, Emily; University of Oxford, Nuffield Department of Primary Care Health Sciences<br>Savic, Sinisa; University of Leeds Faculty of Medicine and Health<br>Savic, Louise; University of Leeds Faculty of Medicine and Health<br>Porter, Catherine; University of Leeds, Healthcare Associated Infection Group<br>Fielding, Joanne; University of Leeds, Healthcare Associated Infection Group<br>Butler, C; University of Oxford,<br>Pavitt, Sue; University of Leeds<br>Sandoe, Jonathan; University of Leeds Faculty of Medicine and Health<br>Tonkin-Crine, Sarah ; University of Oxford, Nuffield Department of Primary Care Health Science

Keywords:	GENERAL MEDICINE (see Internal Medicine), INFECTIOUS DISEASES, Allergy < THORACIC MEDICINE

SCHOLARONE™
Manuscripts

I, the Submitting Author has the right to grant and does grant on behalf of all authors of the Work (as defined in the below author licence), an exclusive licence and/or a non-exclusive licence for contributions from authors who are: i) UK Crown employees; ii) where BMJ has agreed a CC-BY licence shall apply, and/or iii) in accordance with the terms applicable for US Federal Government officers or employees acting as part of their official duties; on a worldwide, perpetual, irrevocable, royalty-free basis to BMJ Publishing Group Ltd ("BMJ") its licensees and where the relevant Journal is co-owned by BMJ to the co-owners of the Journal, to publish the Work in this journal and any other BMJ products and to exploit all rights, as set out in our licence.

The Submitting Author accepts and understands that any supply made under these terms is made by BMJ to the Submitting Author unless you are acting as an employee on behalf of your employer or a postgraduate student of an affiliated institution which is paying any applicable article publishing charge ("APC") for Open Access articles. Where the Submitting Author wishes to make the Work available on an Open Access basis (and intends to pay the relevant APC), the terms of reuse of such Open Access shall be governed by a Creative Commons licence – details of these licences and which Creative Commons licence will apply to this Work are set out in our licence referred to above.

Other than as permitted in any relevant BMJ Author's Self Archiving Policies, I confirm this Work has not been accepted for publication elsewhere, is not being considered for publication elsewhere and does not duplicate material already published. I confirm all authors consent to publication of this Work and authorise the granting of this licence.

For peer review only

Developing a behavioural intervention package to identify and amend incorrect penicillin allergy records in UK general practice

Corresponding author: Marta Santillo, Nuffield Department of Primary Care Health Sciences, University of Oxford, Radcliffe Observatory Quarter, Woodstock Road, Oxford, OX2 6GG, marta.santillo@phc.ox.ac.uk, 01865617935

Santillo, M. [1], Wanat, M. [1], Davoudianfar, M. [1] , Bongard, E. [1], Savic, S. [2] Savic, L. [2], Porter, C. E. [3], Fielding, J. [3], Butler C. [1], Pavitt, S. H. [4], Sandoe, J. A. T. [3] , Tonkin-Crine, S. [1,5]On behalf of the ALABAMA team.

[1] Nuffield Department of Primary Care Health Sciences, University of Oxford, Radcliffe Observatory Quarter, Woodstock Road, Oxford, OX2 6GG , UK

[2] Faculty of Medicine and Health, University of Leeds and Leeds Teaching Hospitals NHS Trust, Leeds LS1 3EX, UK

[3] Healthcare Associated Infection Group, University of Leeds and Leeds Teaching Hospitals NHS Trust, Leeds LS1 3EX, UK

[4] Dental Translational and Clinical Research Unit, Faculty of Medicine and Health, Worsley Building, Clarendon Way, University of Leeds, LS2 9LU, UK

[5] NIHR Health Protection Research Unit in Healthcare Associated Infections and Antimicrobial Resistance, University of Oxford, UK

SHORT RUNNING TITLE: Intervening to amend incorrect penicillin allergy records in general practice

KEYWORDS: intervention development, penicillin allergy testing, general practice

Words: 3923/4000

For peer review only - http://bmjopen.bmj.com/site/about/guidelines.xhtml

For peer review only

ABSTRACT

Words: 288/300

Objectives To develop a behavioural intervention package to support clinicians and patients to identify and amend incorrect penicillin allergy records in English general practice. The intervention aimed to: 1) support general practice clinicians to refer patients for penicillin allergy testing (PAT), 2) support patients to attend for PAT and 3) support clinicians and patients to prescribe or consume penicillin, when indicated, following a negative PAT result.

Methods Theory-based, evidence-based and person-based approaches were utilised in the intervention development. We used evidence from a rapid review, two qualitative studies, one with patients and one with general practice clinicians, and expert consultation with the clinical research team in order to identify the intervention guiding principles and develop an intervention plan. Barriers and facilitators to the target behaviours were mapped to behaviour change theory in order to describe the proposed mechanisms of change. In the final stage, think-aloud interviews and consultations with the clinical research team were conducted to optimise intervention materials.

Results The collated evidence showed that the key barriers to referral of patients by clinicians were limited experience of referral and limited knowledge of referral criteria and PAT. Barriers for patients attending PAT were lack of knowledge of the benefits of testing and lack of motivation to get tested. Intervention materials were designed and developed to address these barriers.

Conclusions We present a novel behavioural intervention package designed to address the multiple barriers to uptake of PAT in general practice by clinicians and patients. The intervention development details how behaviour change techniques have been incorporated to hypothesise how the intervention is likely to work to help amend incorrect penicillin allergy records. The intervention will go on to be tested in a feasibility trial and randomised controlled trial in England.

For peer review only - http://bmjopen.bmj.com/site/about/guidelines.xhtml

ARTICLE SUMMARY

Strengths and limitations of this study

- This research describes the development of a novel behavioural intervention package which will be part of a 'Penicillin Allergy Assessment Pathway' (PAAP) to help identify and amend incorrect penicillin allergy records in English general practice.
- Theory-, evidence- and person-based approaches were used in the intervention development which consisted of three iterative stages: collating the evidence on views and experiences of PAT, intervention development and optimisation of intervention materials.
- This approach details the systematic process by which intervention development was conducted, informing intervention developers and policy makers on how the intervention components are hypothesized to change clinician and patient target behaviours.
- The views of other general practice prescribers should be considered in future research.
- Further work will examine the feasibility of using intervention materials in general practice.

For peer review only

For peer review only - http://bmjopen.bmj.com/site/about/guidelines.xhtml

BACKGROUND

Incorrect penicillin allergy records are common, as side effects and symptoms of illness can be mistaken for allergic reaction symptoms [1]. About 6% of the UK general practice population has a record of a penicillin allergy but fewer than 10% of these patients are likely to be truly allergic [2]. As a result, a significant portion of the population is denied effective first line antibiotics for many common infections. Moreover, penicillin allergy records are also linked to antimicrobial resistance (AMR): evidence suggests that patients with a penicillin allergy label are more likely to be prescribed broad spectrum antibiotics and to acquire antibiotic resistance infections [3 4].

Penicillin allergy testing (PAT) is already provided in the NHS in specialist clinics [5] and offers the opportunity to confirm or discount a penicillin allergy label. This service is only available to a subset of patients following NICE guidance [6]. Current assessment is performed over two clinic visits.

The ALABAMA (Allergy Antibiotics and Microbial resistance) study aims to develop a behavioural intervention package for general practice to effectively amend incorrect penicillin allergy records. The intervention package is designed to target both general practice clinicians and patients with a suspected incorrect penicillin allergy record. It introduces a pre-emptive 'penicillin allergy assessment pathway' (PAAP) which targets patients assessed as "low risk" of true allergy, who are not at risk of anaphylaxis or other severe adverse reactions, and it aims to streamline the test process by undertaking patient history screening in general practice (Stage 1) and introducing an efficient one-stop procedure at a hospital clinic for the penicillin allergy test (PAT). The test includes either a skin test (ST), testing penicillin solution on the forearm (Stage 2), and oral challenge test (OCT), taking doses of penicillin solution over time (Stage 3), or just OCT depending on the individual patient. Following the PAT, patients and practices will receive confirmation of a patient's allergy status.

For peer review only - http://bmjopen.bmj.com/site/about/guidelines.xhtml

This paper describes the planning, development and optimisation of the ALABAMA PAAP intervention package.

METHODS AND RESULTS

Intervention planning methodology

We followed an integrated approach to intervention development that combines theory-, evidence- and person-based approaches [7-9]. This approach has been successfully used in the development of a variety of behavioural interventions including for reduction of antibiotic prescriptions in European general practice [10]. The ALABAMA intervention package was developed in three stages described below.

Patient and Public Involvement

The AlABAMA programme grant involved PPI members, as co-applicants, and study advisors from the start so research questions were informed by their input. Patients were involved as participants within the exploratory qualitative work and their experiences were used to inform intervention development. Patients were again involved in think aloud interviews to help develop specific materials within the intervention package. Results of all work from the AlABAMA programme will be disseminated with the help from our PPI co-apps and our existing PPI networks towards the end of the programme grant.

The methods and key results for each stage are presented below.

Stage 1: Collating and analysing evidence

Rapid review methods

The full rapid review is available elsewhere [11]. Rapid review is a form of synthesis which supports the review of existing evidence in a timely manner [12]. It aimed to explore clinicians' and patients' views and experiences of penicillin allergy testing services.

For peer review only - http://bmjopen.bmj.com/site/about/guidelines.xhtml

Outcomes

The review identified only two studies which reported patients' views of PAT. All patients thought that PAT would provide valuable medical information. The majority of patients reported limited knowledge of penicillin allergy and PAT services. Clinicians reported several barriers to referring patients for PAT. These included difficulties establishing the allergy history, lack of knowledge on referral processes and organisational pressures making allergy testing a low priority. A number of clinicians and patients reported being reluctant to prescribe or consume penicillin after a negative PAT result.

Qualitative methods

Full details of the qualitative work are available elsewhere[13]. Two qualitative studies were undertaken by MW; one interviewing 31 patients with a penicillin allergy record (16 with previous experience of PAT) and the second interviewing 19 general practitioners. The aim was to identify patients' and clinicians' views on the barriers and facilitators for PAT and antibiotic use after a negative test. Semi-structured interviews were conducted over the phone by an experienced qualitative researcher (PhD qualified) with substantial previous experience of conducting qualitative research. Patients were identified from a general adult hospital allergy clinic and from general practices in the same geographical area. Clinicians were identified in general practices and by the local microbiology services.

Outcomes

An inductive thematic analysis approach was used to analyse data. The majority of patients who were motivated to get tested had experienced a negative consequence of having a penicillin allergy label (such as limited availability of antibiotics they could use). Patients reported concerns about the possibility of having an allergic reaction during PAT; the degree of the severity of their previous reactions affected how apprehensive they were about the test. Moreover, when the test was perceived as more invasive, e.g. the OCT compared to the ST, patients reported being more

For peer review only

For peer review only - http://bmjopen.bmj.com/site/about/guidelines.xhtml

concerned about PAT. Patients were also concerned about how much they would be monitored during the test and highlighted the importance of feeling informed, safe and observed by qualified professionals. Some patients reported being unsure about a negative test result and feeling anxious about taking penicillin if prescribed, as they doubted whether the test result was accurate.

Clinicians reported that they often felt that penicillin allergy records were incorrect however reported uncertainty about how to identify patients with true penicillin allergy and were reluctant to amend medical records without objective evidence. Penicillin allergy status was not seen to be a major problem in general practice due to the availability of alternative antibiotics and clinicians struggled to identify the risks of incorrect allergy records. Clinicians were seen to differ in their consultation styles when speaking to patients about their antibiotic prescribing decisions and allergy status. They reported lack of experience of PAT services and the need for more information on referral criteria. Regarding the process of changing a patients' record after a negative test result, clinicians reported being happy to update medical records on directions from secondary care but were unsure who was responsible for making sure that patients understood allergy test results.

Expert Discussions

As part of this first stage we consulted our wider clinical research team using monthly tele-conferences and emails to gain their feedback on several aspects of the intervention development, such as the interpretation of evidence collated in the rapid review and the qualitative studies, the development of early iterations of the intervention materials, and the development of the initial intervention plan and components. The clinical research team included a consultant immunologist, a consultant microbiologist, a consultant anaesthetist, a general practitioner and professors with expertise in applied health research.

For peer review only - http://bmjopen.bmj.com/site/about/guidelines.xhtml

Stage 2: Intervention planning and development

Creating guiding principles

In line with the person-based approach[7] brief guiding principles were created to be consulted through the whole intervention development process. This ensured that the intervention met the original objectives. Based on the findings of the rapid review, qualitative interviews and expert discussion, the characteristics and behavioural needs of the target users were identified. Guiding principles were then created to outline the intervention objectives and the key design features which addressed them.

Outcomes

Table 1 presents the ALABAMA guiding principles. These focused on increasing confidence to refer and attend for PAT and increasing motivation to prescribe/consume penicillin following a negative PAT result. Guiding principles also included increasing clinician confidence in discussing penicillin allergy with patients and improving communication between primary and secondary care about penicillin allergy status. Lastly the intervention needed to present the PAAP as reliable and trusted and provide accessible and easy to use materials for clinicians and patients.

For peer review only - http://bmjopen.bmj.com/site/about/guidelines.xhtml

Table 1: Guiding Principles for the ALABAMA intervention package.

PAAP =Penicillin Allergy Assessment Pathway; PAT = Penicillin Allergy Testing

Intervention design objectives	Key features
To present the PAAP as a reliable and trusted approach to confirm allergy status	• Present the PAAP as a trusted/scientific/official way to get a confirmed PAT result, for both clinicians and patients
To increase clinician's confidence in referring patients, and patients' motivation to attend, for PAT	• Provide evidence on the potential harms of an incorrect penicillin allergy records and the process of testing in the Information Pack<br>• Provide opportunity to address patients' concerns about potential benefits and risks of testing by both clinicians and allergists during consultations before the allergy test and during the appointment at the immunology clinic
To motivate clinicians/patients to prescribe/take penicillin following a negative PAT test result	• Provide information to clinicians about the clinical meaning of a test result and its implication as part of the Information Pack<br>• Provide information to patients about the PAT (process) and the potential benefits of being able to take penicillin in the Pre-test Intervention Booklet
To increase clinicians' ability to discuss PAT with patients	• Provide training for general practice clinicians about penicillin allergy and PAT, including its accuracy, implications, benefits
To improve communication between primary and secondary care so that allergy status records are correct	• Provide a clear and consistent approach to de-labelling with support from colleagues in secondary care and preventing re-labelling in the future<br>• Provide information about who is responsible for ensuring patients understand the results and for updating the medical records during the site training and in the Information Pack

For peer review only - http://bmjopen.bmj.com/site/about/guidelines.xhtml

Intervention design objectives	Key features
	• Provide clear and precise documentation of side effects during future courses of penicillin in the Information Pack
To provide easily accessible tools that are suitable for use by general practice clinicians	• Make interventions materials for clinicians accessible, short, easy to follow, easy to implement and not increase workload
To provide easily accessible resources for patients	• Make patients materials brief, easy to read, accessible<br>• Make materials easy to carry with them at all time and that provide evidence of the results of their test

For peer review only

Behavioural analysis

The aim of the behavioural analysis was to use behaviour change theory[14] to describe the content of the ALABAMA intervention package and map the evidence from the rapid review, qualitative studies and expert consultations.

Methods

The first step of the behavioural analysis process was to identify target behaviours, their barriers and facilitators, and how intervention components would support desired behaviour change based on evidence collated in stage 1. Intervention components were mapped to the TDF framework[15] and the Behaviour Change Wheel (BCW)[14] referring to the Behaviour Change Techniques Taxonomy (BCTv1)[16]. This produced a list of TDF barriers, target constructs (what needs to change for the behaviour to occur), intervention functions (ways an intervention can change behaviour) and behaviour change techniques used for each of the barriers/facilitators.

Outcomes

The full behavioural analysis is presented in supplementary materials (S1). Firstly, we identified barriers and facilitators to referral of low risk patients to PAT and patient attendance to PAT. The

For peer review only - http://bmjopen.bmj.com/site/about/guidelines.xhtml

analysis highlighted that both clinicians' and patients' knowledge and perceptions of penicillin allergy and test procedures could be modified; information needed to be supported by scientific evidence for clinicians and patients to be reassured that the test was safe. We designed a resource for clinicians entitled "Penicillin Allergy Testing: Information for general practice" which contained information on penicillin allergy and PAAP procedures. As part of the ALABAMA trial, this will be supported by site training and working instructions which provide practical guidance on screening patients and referral to PAT (relevant BCTs for clinicians were 'information about antecedents' and 'information about health consequences'). For patients we developed two patient booklets, one to be provided prior to PAT and one following a negative test result. All patients, on entering the trial, will have a consultation with a GP to answer questions and address concerns about PAT. We developed a patient booklet entitled "Penicillin Allergy Testing: going for a test" which included information on PAAP procedures and PAT safety (relevant BCTs for patients were 'information about health consequences' and 'feedback on outcomes of the behaviour').

The barriers to the prescription and consumption of first-line penicillin following a negative test result were patient and clinician beliefs about the accuracy of PAT and whether taking penicillin was safe. Clinicians also needed reassurance that colleagues saw de-labelling as beneficial and resources to support them in changing incorrect penicillin allergy records. We developed a second patient booklet entitled "Penicillin Allergy Testing: a negative test result", which contained information about which antibiotics patients could safely take in the future following a negative test result, a negative result intervention card and a result letter which confirmed the patient allergy status to penicillin (relevant BCTs were 'social support' and 'restructuring of the social and physical environment'). As part of the trial, clinicians received working instructions, which contained guidance on how to change the patient allergy label in medical records, result letter which confirmed the patient allergy status to penicillin, and an electronic-pop up, which included a reminder of the patient's new allergy status (relevant BCTs were 'feedback on outcomes of behaviour' and 'adding objects to the environment').

For peer review only - http://bmjopen.bmj.com/site/about/guidelines.xhtml

Logic modelling

The next step included the development of a logic model, which summarised the behavioural analysis, providing a diagrammatic representation of the hypothesised processes and causal pathways from the intervention components to the desired outcomes.[17 18]

Methods

The research team opted for a process oriented iterative logic model which was refined during the whole intervention development stage.

Outcomes

The logic model (Figure 1) included four components:

Intervention components and techniques: Intervention components were organised based on the two target groups (clinicians and patients). Intervention techniques summarised the BCTs used as part of the intervention which were identified in the behavioural analysis.

Intervention processes: These were the psychological factors which explained the relationships between the intervention components and the outcome of the intervention: each intervention technique was hypothesised to mainly affect one of these processes. As part of the intervention, clinicians would receive information on penicillin allergy and implications of incorrect allergy records in order to increase their knowledge of PAT and allergies. In addition to this, providing medical and scientific evidence and current guidelines on how to perform PAT would change their professional identity related to their role in the referral process and act as reinforcement to increase their motivation to refer patients in the future. Regarding the processes affecting patient's attendance of PAT, providing evidence of penicillin as best treatment and the safety of PAT would increase their beliefs of positive consequences of accurate records; informing them of common reactions during PAT and reassuring them of monitoring at the clinic and at home would increase their knowledge on

For peer review only - http://bmjopen.bmj.com/site/about/guidelines.xhtml

PAT, decrease their negative emotions, such as anxiety, and act as positive reinforcement to attend the clinic.

Both patients and clinicians would receive evidence of the accuracy of PAT, and of safety of prescribing penicillin after a negative test result, in order to increase their belief about positive consequences of prescribing penicillin after a negative test result. Finally, providing a negative test result card to use with clinicians would affect patients' expectations of social influences regarding clinician's acceptance of the test result.

Purported mediators: These are the target behaviours of the intervention which directly affect the outcomes. In the logic model the assessment of potential incorrect penicillin records was operationalised as the referral of low risk patients to PAT and patient attendance at PAT. The introduction of the PAAP was hypothesized to affect the change of incorrect penicillin allergy records (clinician changing medical records and patient acceptance of change of penicillin allergy status) which would ultimately affect the consumption of penicillin.

Outcomes: The behavioural outcomes of the model were the prescription and consumption of first-line penicillin when indicated. The main outcome of the trial is "treatment response failure" to assess patient recovery from infection when taking antibiotics.

Stage 3: Optimising the intervention materials

Think-aloud interviews with GPs

Methods

Think aloud telephone interviews were conducted with 6 participants by MW; 2 additional participants provided feedback via email. Interviews asked about views of each section of the "Information for general practice leaflet" developed for clinicians.

For peer review only - http://bmjopen.bmj.com/site/about/guidelines.xhtml

Outcomes

The leaflet was well received. Participants reported that it was informative, useful and generally easy to read. The participants perceived it not only as information for themselves, but also a tool to use in a consultation with patients. Some participants felt that they knew about the consequences of incorrect penicillin allergy record; and therefore the leaflet could be shortened. Most participants understood the testing stages; however, a couple of participants were confused about which stages of the test patients could skip. One participant wanted exact doses of penicillin specific (rather than just amounts). Regarding the section on patient discussions, some clinicians felt that there was no need to discuss the test with patients. Participants queried whether being tested with amoxicillin meant that the patient could now take all penicillin based antibiotics and wanted more information.

Clinicians' feedback was collated and organised in a 'table of changes' (supplementary materials) where suggested changes were listed and given a level of priority for that change, following the MoScoW framework [19], and the source of the suggested change (expert opinion, research team, clinical research team, literature review). Changes to the 'Information for general practice' included changes to the title, to the exact doses of penicillin given to the patients during the test, information about side effects and information about which antibiotics patients with a negative test result can take safely.

Think-aloud interviews with patients

Methods

Think aloud interviews telephone interviews were conducted with 7 patients (3 with experience of PAT and 4 with no experience) by MW. Interviews asked their views about the two patient booklets ("Penicillin Allergy Testing: going for a test", "Penicillin Allergy testing: a negative test result") and the intervention card.

For peer review only - http://bmjopen.bmj.com/site/about/guidelines.xhtml

Outcomes

The booklets and intervention card were very well received by the participants. Participants considered the booklets to have the right amount of information and felt they were generally easy to read. Patients reported that the booklets convinced them that going for a PAT could be beneficial. They felt that they could relate to the description of how people were given penicillin allergy labels. Patients thought the description of the test was clear and they knew what to expect. Statistics about the prevalence of allergy were not always understood by the participants, as the participants often thought that 1 in 10 people are allergic and they wanted a more visual presentation of this key information. Participants were unsure what narrow and broad spectrum antibiotics were and did not recognise MRSA abbreviation. Participants did not always know that penicillin is more than one antibiotic. The participants wanted to have a separate paragraph on what could happen during the test and what could happen during three days of taking penicillin at home. They also wanted reassurance that three days would be enough to detect delayed reactions. The participants wanted more reassurance that after being tested with one type of penicillin (e.g. amoxicillin), it would mean that they could safely take all penicillin antibiotics. The participants were slightly concerned about the risk of allergic reaction in the future (despite negative test results).

Patient feedback was collated in a table of changes. Changes made to the booklets were the selection of new images of patients for the front cover, inclusion of definitions of narrow and broad spectrum antibiotics, and reassurance that 3 days of oral challenge would be enough to detect delayed reactions to penicillin.

Intervention components

All Working Instructions developed to support clinicians and research nurses activities as part of the ALABAMA intervention package were shown to a group of clinicians to gain their feedback on content and layout. Among the clinicians who provided feedback there were two practice managers,

For peer review only - http://bmjopen.bmj.com/site/about/guidelines.xhtml

and one nurse. Their overall feedback was positive and the main changes to the intervention materials included the identification of the best way of updating the patient's medical records after PAT, and the introduction of screenshots of the medical record in the working instructions.

All participants letter (patient appointment letter, patient result letter, clinician result letter) were developed among the wider clinical and research team in order to make them effective in motivating patient to attend the penicillin allergy testing and in order to persuade clinician to change patient records and prescribe penicillin after a negative test result, and patient to consume penicillin after a negative test result.

At the end of the intervention development stage, a description of the intervention was completed following the TIDieR [20] guidance (supplementary materials S2) together with a description of the intervention components for clinicians and patients (Table 2).

For peer review only

Table 2: Overview of the key intervention components of the AlABAMA intervention package.

PAT = Penicillin Allergy Testing

Intervention components	Description
**For clinicians**	
'Penicillin Allergy Testing: Information for general practice' sheet	Information sheet which includes evidence-based information to increase knowledge about penicillin allergy testing and motivation to refer patients to PAT and prescribe penicillin after a negative PAT result
Electronic health record pop-up	Electronic pop-up on medical records which tells clinicians that patient has had a negative test result and that their allergy status needs to be amended
Allergy result letter	A letter is sent to the GP to inform them of the patient's penicillin allergy test result, including details on which test(s) they undertook, whether it is safe or not to prescribe penicillin in the future and instructions on how to change the allergy label
Consultation with patients	Discussions with patients to check eligibility for the trial and to answer any queries about the trial and PAAP testing
Site Training and working instructions	Training in trial procedures delivered to GP leads including provision of information on penicillin allergy de-labelling, the referral process, the three stages of the PAAP and the interpretation of test results
**For patients in intervention arm**	
"Penicillin allergy testing: going for a test" booklet	A booklet to inform patients, in the PAAP intervention arm, about incorrect allergy records, how they may benefit from having a penicillin allergy test and what the test involves
Penicillin Allergy Test appointment letter	The letter includes information on the PAAP procedures in hospital, including pre-test assessment and monitoring during the test and at home

For peer review only - http://bmjopen.bmj.com/site/about/guidelines.xhtml

Penicillin Allergy Assessment Pathway (PAAP)	Appointment at the immunology clinic for patients in the PAAP intervention arm. At the appointment patients will complete Stage 1, Stage 2 and/or Stage 3 of the PAAP pathway
Allergy test result letter	Patient will receive a letter with information on the result of their allergy test and whether it is safe or not to take penicillin in the future
"Penicillin allergy testing: a negative test result" booklet	A booklet on the reliability of the test results and consequences of a negative test result (sent with allergy test result letter).
Post-test intervention card	Laminated credit card-sized card which says which test the patient has had and confirms the negative allergy result
For all patients in control and intervention arm	
An invitation letter and Participant information Sheet	An invitation letter and Participant information Sheet on the purpose of the trial and what the research study would involve for patient participants
Discussion with clinicians	Discussion with clinicians about attending the test to ask any queries around the benefits of taking the test and why removing the incorrect record might be good

The iterative process of intervention development and optimisation of intervention materials informed by the rapid review, qualitative work, expert consultation and think-aloud interviews is shown in Figure 2 and 3. It presents the example of this process for the development of one section of the "Penicillin allergy testing: going for a test" patient booklet and the "Information for General Practice" leaflet for clinicians.

DISCUSSION

We the development of the ALABAMA intervention package which seeks to change behaviours to facilitate PAT to identify and amend incorrect allergy records in English general practice, that the PAT result is appropriately documented, and impacts on antibiotic prescribing and consumption. The

For peer review only - http://bmjopen.bmj.com/site/about/guidelines.xhtml

approach used here has previously been used for the development of behaviour change interventions targeted to reduce antibiotic prescribing by clinicians in primary[21] and secondary care[22], but it is the first time that this approach has been used to develop an intervention to amend incorrect penicillin allergy records. The transparency of the intervention development process will inform intervention developers on how this methodology could be used in different contexts, and will facilitate the comparison with other interventions which have used similar processes.

The ALABAMA intervention package targets clinician referral of patients for PAT and updating incorrect penicillin allergy records; factors previously identified in previous qualitative research as barriers to effective penicillin allergy de-labelling.[11 13] Recent exploration of clinician reported barriers and enablers to identifying and de-labelling hospital in patients with incorrect penicillin allergy records has highlighted the need to introduce patient education concerning the risks of avoiding penicillin.[23] Inconsistencies in the management of penicillin allergic patients were reported, together with a lack of time to discuss allergy testing, and the need to improve communication between primary and secondary care about patient allergy status, as well as updating of patient medical records.[23] A previous exploration of views about implementing de-labelling of patients ahead of elective surgery identified barriers to implementing it on a large scale, such as human factors linked to anxiety and financial implications. The human factors were: lack of interest from patients in undertaking an allergy test; lack of acceptance of the test result among clinicians; high proportion of patient re-labelled themselves after a negative testing for penicillin allergy or re-labelling by health care professionals. The financial barrier was significant despite long-term cost benefit, as there is an upfront cost to perform the test.[24]

The evidence collated from the rapid review and qualitative interviews allowed in-depth understanding of participant needs (lack of knowledge of PAT, lack of knowledge of negative consequences of allergy labels) and which behavioural influences needed to be modified as part of the intervention. The mapping of these behavioural influences and the ALABAMA intervention

For peer review only - http://bmjopen.bmj.com/site/about/guidelines.xhtml

package components to behaviour change theory and the logic model allowed a transparent reporting of the psychological processes that are hypothesized to explain the effect of the intervention components on the trial outcomes. More specifically it highlighted the BCTs used in each component of the ALABAMA intervention package and how they addressed the barriers identified in the rapid review and qualitative work. It also explained which psychological mechanisms were changed by the intervention components (clinicians' knowledge on penicillin allergy and PAT procedures, patients' beliefs about positive consequences of taking the PAT) and which target behaviours (referral and attendance to PAT, prescription and consumption of penicillin first line) affected the intervention primary outcome (the treatment "response failure"). Think-aloud interviews with patients and clinicians, and in-depth feedback from the clinical research team on the intervention materials, highlighted changes that needed to be made in order to increase their acceptability and potential effectiveness.

A limitation of the AlABAMA intervention package is to how widely applicable it might be. Allergy services across the UK vary significantly, and access to specialist testing ranges widely. The AlABAMA programme focuses on one geographical area (North of England), which is covered by the specialist unit used in the AlABAMA trial. Although the intervention is centred around functionality that has been incorporated into SystemOne, which is widely used, the intervention package is not necessarily suitable for use in other areas of England, the UK or wider and contextual factors to delivery should be considered. Moreover, it is only a small group of patients (around 25-30%) who are suitable to undergo the abbreviated test (patient history, skin test and oral challenge test). Many will still require full assessment by an immunologist or allergist as per current guidelines. Cost-effectiveness analysis of the PAT and intervention package as a whole will be carried out the in upcoming AlABAMA trial.

For peer review only - http://bmjopen.bmj.com/site/about/guidelines.xhtml

Conclusions

Current clinical practice involves referral to penicillin allergy testing with little attention to other elements of the pathway that help to ensure that testing impacts positively on patient care. This study presents the development of a behavioural intervention package to support the process of amending incorrect penicillin allergy records. Numerous barriers to the uptake of PAT have been identified as well as penicillin prescribing and consumption following a negative test. We have identified relevant behaviour change techniques to inform the development of the AlABAMA intervention package to overcome these barriers. The intervention is currently being tested in a feasibility trial in primary care to lead on to a randomised-controlled trial.

Acknowledgements: The AlABAMA team consists of: Jenny Boards, Emily Bongard, Christopher C Butler, Mina Davoudianfar, Mandy East, Joanne Fielding, Philip Howard, Sue H Pavitt, Catherine E Porter, Jonathan AT Sandoe, Marta Santillo, Louise Savic, Sinisa Savic, Bethany Shinkins, Sarah Tonkin-Crine, Marta Wanat, Robert West, Ly-Mee Yu.

Author contributions: All authors designed the study. MW, STC and MS led the intervention development, with input from the other co-authors. MW was responsible for the rapid review, recruitment, carrying out the interviews and analysing the data, under STC's supervision. MS drafted the manuscript with support from STC. All authors critically reviewed the manuscript and approved the final version of the manuscript.

Funding: This study summarises independent research funded by the National Institute for Health Research (NIHR) under its Programme Grants for Applied Research Programme (Grant Reference Number RP-PG-1214-20007). STC received additional funding from the National Institute for Health Research Health Protection Research Unit (NIHR HPRU) in Healthcare Associated Infections and Antimicrobial Resistance at the University of Oxford in partnership with Public Health England (PHE)

For peer review only - http://bmjopen.bmj.com/site/about/guidelines.xhtml

[HPRU-2012-10041]. The research is supported by the National Institute for Health Research (NIHR) infrastructure at Leeds. The funder had no role in the design of the study; in the collection, analyses, or interpretation of data; in the writing of the manuscript, or in the decision to publish.

Disclaimer: The views expressed are those from the authors and not necessary those of the NHS, NIHR or the Department of Health and Social Care.

Competing Interests: The authors have received funding from the National Institute for Health Research.

Patient Consent: Not required

Ethics approval: This study received ethical approval from the London Bridge Research Ethics Committee (Ref: 19/LO/0176).

Data sharing statements: No additional data available

REFERENCES

1. McLean-Tooke A, Aldridge C, Stroud C, et al. Practical management of antibiotic allergy in adults. *Journal of Clinical Pathology* 2011;64(3):192-99. doi: 10.1136/jcp.2010.077289

2. Borch JE, Andersen KE, Bindslev-Jensen C. The prevalence of suspected and challenge-verified penicillin allergy in a university hospital population. *Basic Clin Pharmacol Toxicol* 2006;98(4):357-62. doi: 10.1111/j.1742-7843.2006.pto_230.x

3. Reddy V, Baman NS, Whitener C, et al. Drug Resistant Infections with Methicillin-Resistant Staphylococcus Aureus, Clostridium Difficile, and Vancomycin Resistant Enterococcus

For peer review only - http://bmjopen.bmj.com/site/about/guidelines.xhtml

Are Associated with a Higher Prevalence of Penicillin Allergy. *J Allergy Clin Immunol* 2013;131(2):AB170-AB70. doi: 10.1016/j.jaci.2012.12.1269

4. West RM, Smith CJ, Pavitt SH, et al. "Warning: allergic to penicillin': association between penicillin allergy status in 2.3 million NHS general practice electronic health records, antibiotic prescribing and health outcomes. *J Antimicrob Chemother* 2019;74(7):2075-82. doi: 10.1093/jac/dkz127

5. Krishna MT, Huissoon AP, Li M, et al. Enhancing antibiotic stewardship by tackling "spurious" penicillin allergy. *Clin Exp Allergy* 2017;47(11):1362-73. doi: 10.1111/cea.13044

6. NICE. Drug allergy: giagnosis and management of drug allergy in adults, children and young poeple. NICE clinical guideline 183. 2014

7. Yardley L, Morrison L, Bradbury K, et al. The Person-Based Approach to Intervention Development: Application to Digital Health-Related Behavior Change Interventions. *Journal of Medical Internet Research* 2015;17(1) doi: 10.2196/jmir.4055

8. Greenwell K, Sivyer K, Vedhara K, et al. Intervention planning for the REDUCE maintenance intervention: a digital intervention to reduce reulceration risk among patients with a history of diabetic foot ulcers. *BMJ Open* 2018;8(5):12. doi: 10.1136/bmjopen-2017-019865

9. Band R, Bradbury K, Morton K, et al. Intervention planning for a digital intervention for self-management of hypertension: a theory-, evidence- and person- based approach. *Implementation Science* 2017;12 doi: 10.1186/s13012-017-0553-4

10. Little P, Stuart B, Francis N, et al. Effects of internet-based training on antibiotic prescribing rates for acute respiratory-tract infections: a multinational, cluster, randomised, factorial, controlled trial. *The Lancet* 2013;382(9899):1175-82.

For peer review only - http://bmjopen.bmj.com/site/about/guidelines.xhtml

11. Wanat M, Anthierens S, Butler CC, et al. Patient and Prescriber Views of Penicillin Allergy Testing and Subsequent Antibiotic Use: A Rapid Review. *Antibiotics-Basel* 2018;7(3) doi: 10.3390/antibiotics7030071

12. Khangura S, Polisena J, Clifford TJ, et al. RAPID REVIEW: AN EMERGING APPROACH TO EVIDENCE SYNTHESIS IN HEALTH TECHNOLOGY ASSESSMENT. *International Journal of Technology Assessment in Health Care* 2014;30(1):20-27. doi: 10.1017/s0266462313000664

13. Wanat M, Anthierens S, Butler CC, et al. Patient and Primary Care Physician Perceptions of Penicillin Allergy Testing and Subsequent Use of Penicillin-Containing Antibiotics: A Qualitative Study. *Journal of Allergy and Clinical Immunology-in Practice* 2019;7(6):1888-+. doi: 10.1016/j.jaip.2019.02.036

14. Michie S, van Stralen MM, West R. The behaviour change wheel: A new method for characterising and designing behaviour change interventions. *Implementation Science* 2011;6 doi: 10.1186/1748-5908-6-42

15. Atkins L, Francis J, Islam R, et al. A guide to using the Theoretical Domains Framework of behaviour change to investigate implementation problems. *Implementation Science* 2017;12 doi: 10.1186/s13012-017-0605-9

16. Michie S, Richardson M, Johnston M, et al. The Behavior Change Technique Taxonomy (v1) of 93 Hierarchically Clustered Techniques: Building an International Consensus for the Reporting of Behavior Change Interventions. *Annals of Behavioral Medicine* 2013;46(1):81-95. doi: 10.1007/s12160-013-9486-6

17. Baxter S, Killoran A, Kelly MP, et al. Synthesizing diverse evidence: the use of primary qualitative data analysis methods and logic models in public health reviews. *Public Health* 2010;124(2):99-106. doi: 10.1016/j.puhe.2010.01.002

For peer review only - http://bmjopen.bmj.com/site/about/guidelines.xhtml

18. Baxter SK, Blank L, Woods HB, et al. Using logic model methods in systematic review synthesis: describing complex pathways in referral management interventions. *Bmc Medical Research Methodology* 2014;14 doi: 10.1186/1471-2288-14-62

19. Bradbury K, Morton K, Band R, et al. Using the Person-Based Approach to optimise a digital intervention for the management of hypertension. *PloS one* 2018;13(5):e0196868.

20. Hoffmann TC, Glasziou PP, Boutron I, et al. Better reporting of interventions: template for intervention description and replication (TIDieR) checklist and guide. *Bmj-British Medical Journal* 2014;348 doi: 10.1136/bmj.g1687

21. Anthierens S, Tonkin-Crine S, Douglas E, et al. General practitioners' views on the acceptability and applicability of a web-based intervention to reduce antibiotic prescribing for acute cough in multiple European countries: a qualitative study prior to a randomised trial. *BMC family practice* 2012;13(1):101.

22. Santillo M, Sivyer K, Krusche A, et al. Intervention planning for Antibiotic Review Kit (ARK): a digital and behavioural intervention to safely review and reduce antibiotic prescriptions in acute and general medicine. *J Antimicrob Chemother* 2019

23. Powell N, Wilcock M, Roberts N, et al. Focus group study exploring the issues and the solutions to incorrect penicillin allergy-labelled patients: an antibiotic stewardship patient safety initiative. *European Journal of Hospital Pharmacy* 2019:ejhpharm-2019-001863.

24. Savic L, Gurr L, Kaura V, et al. Penicillin allergy de-labelling ahead of elective surgery: feasibility and barriers. *Br J Anaesth* 2019;123(1):E110-E16. doi: 10.1016/j.bja.2018.09.009

For peer review only - http://bmjopen.bmj.com/site/about/guidelines.xhtml

Figure 1: AlABAMA logic model

Figure 2: Example of intervention development for patient materials. PAT = Penicillin Allergy Testing

Figure 3: Example of intervention development for clinician materials

For peer review only

For peer review only - http://bmjopen.bmj.com/site/about/guidelines.xhtml

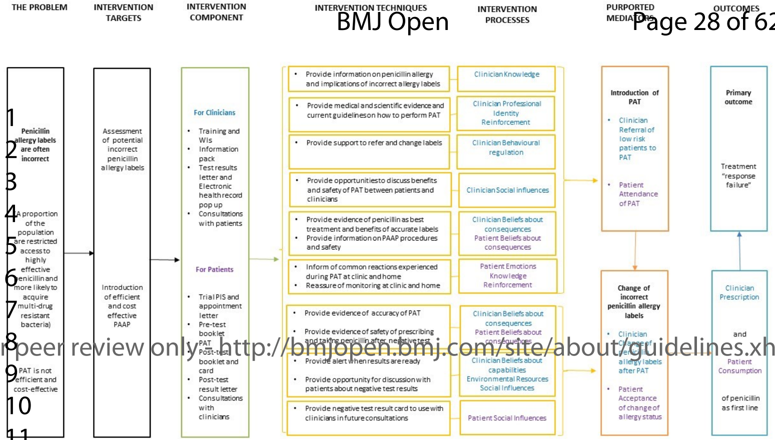

For peer review only - http://bmjopen.bmj.com/site/about/guidelines.xhtml

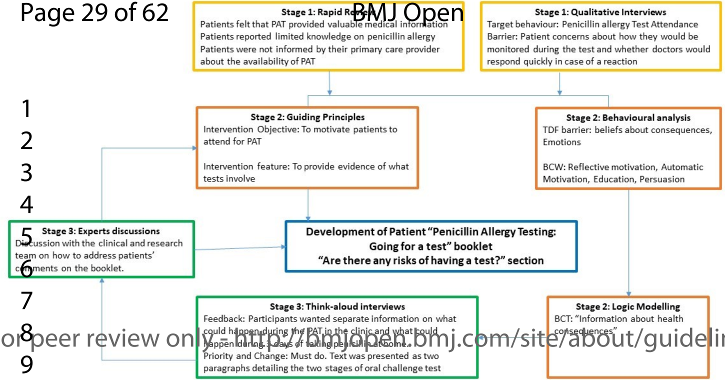

For peer review only - http://bmjopen.bmj.com/site/about/guidelines.xhtml

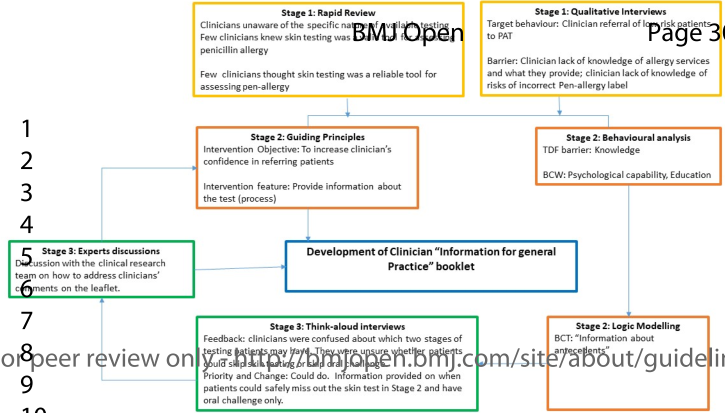

For peer review only - http://bmjopen.bmj.com/site/about/guidelines.xhtml

SUPPLEMENTARY MATERIALS

S1. Behavioural Analysis Table ALABAMA

S2. Table of changes: "Penicillin Allergy Testing: going for a test" booklet

S3. Full description of the AlABAMA (ALlergy AntiBiotics And Microbial resistAnce) Intervention package in accordance with the Template for Intervention Description and Replication (TIDieR) framework.

For peer review only

S1. Behavioural Analysis Table ALABAMA

Target Behaviours:

- Patient attendance at Penicillin Allergy Testing (PAT)
- Patient consumption of penicillin when prescribed after negative test result
- Clinician referral of low risk patients to undertake the Penicillin Allergy Testing (PAT)
- Clinician prescription of penicillin after negative test result

Notes: [QI]: Qualitative Interviews; [RR]: Rapid Review; [LR]: Literature Review; PIS; Participant Information Sheet

Table S1 Behavioural Analysis Table for the AlABAMA intervention

**Barriers / *facilitators* to target behaviours**	**Intervention Component/s**	**Intervention ingredient**	**Theoretical Domains Framework (TDF)**	**Target construct (BCW)**	**Intervention function (BCW)**	**Behaviour Change Technique (using 93 BCT taxonomy v1)**
**Patient attendance at Penicillin Allergy Testing (PAT)**						
**Lack of information around the benefits of having access to penicillin**, when patients didn't experience any negative consequences of the allergy label [QI]	Pre-test intervention booklet<br><br>Trial PIS	• Provide evidence on why penicillin is the preferred and best treatment for infections and kills fewer good bacteria;<br>• Provide information that other antibiotics could be less effective, and could increase the risk of "superbug" infections	Beliefs about consequences	Reflective Motivation	Education<br><br>Persuasion	5.1 information about health consequences
**Difficulty for patients to have access to the right antibiotics** if there is a penicillin allergy [QI]	PAT procedures at allergy clinic	• Provide opportunity to undertake allergy testing	Reinforcement	Automatic motivation	Environmental restructuring	12.2 restructuring of physical environment
Concerns related **to attending** the appointment [QI]	Consultations with clinicians;<br><br>Trial PIS	• Provide information about how the appointment will be arranged and that some travel expenses will be provided	Environmental Context and resources	Physical opportunity	Enablement	1.4 action planning
Concerns whether the **GP will encourage/support** patient to have the test [QI]	Consultations with clinicians<br><br>Trial PIS	• Provide the opportunity to discuss the benefits of the testing with their clinicians	Social influences	Social opportunity	Enablement	3.1 social support (unspecified)

For peer review only

**Barriers /** ***facilitators*** **to target behaviours**	**Intervention Component/s**	**Intervention ingredient**	**Theoretical Domains Framework (TDF)**	**Target construct (BCW)**	**Intervention function (BCW)**	**Behaviour Change Technique (using 93 BCT taxonomy v1)**
**Unsure about what the test is going to involve** and uncertainty around doses of penicillin given during the test [QI]	Pre-test intervention booklet<br><br>Trial PIS	• Provide information about what a penicillin allergy test would involve, explaining the three stages of the testing	Knowledge;<br><br>Beliefs about consequences	Psychological capability<br><br>Reflective Motivation	Education	2.7 feedback on outcomes of behaviour<br><br>5.1 information about health consequences
**Lack of information about** the **credibility** of PAT [*QI]	Consultations with clinicians	• Provide the opportunity to address any concerns about what PAT is and what other options they may have	Knowledge;<br><br>Beliefs about consequences	Psychological capability<br><br>Reflective Motivation	Education	2.7 feedback on outcomes of behaviour<br><br>5.1 information about health consequences
**Concerns that the procedure is not safe** [QI]	Pre-test intervention booklet<br><br>Trial PIS	• Provide information on the common reactions to PAT during the clinic visit and how patients will be treated by medical staff if they experience any reaction	Beliefs about consequences	Reflective Motivation	Persuasion	5.1 information about health consequences
**Concerns about a possible reaction** during test if they have been told by their clinicians for many years to avoid penicillin [QI]	Pre-test intervention booklet	• Provide reassurance that the allergy test is very safe and that serious reactions are very rare.	Emotions	Automatic motivation	Persuasion	5.1 information about emotional consequences
**Apprehension about having a test** for patients with a previous severe allergic reaction [QI]	Pre-test intervention booklet<br><br>Trial PIS	• Provide reassurance that the test will be offered only if the nurses and doctors in the allergy clinic think that the patient is low risk	Emotions	Automatic motivation	Persuasion	5.1 information about emotional consequences

For peer review only

**Barriers / *facilitators* to target behaviours**	**Intervention Component/s**	**Intervention ingredient**	**Theoretical Domains Framework (TDF)**	**Target construct (BCW)**	**Intervention function (BCW)**	**Behaviour Change Technique (using 93 BCT taxonomy v1)**
Concerns about a reaction that could **worsen their overall state of health** when patients have a chronic co-morbidity [QI]	Consultations with clinicians	• Provide reassurance about the safety of test	Beliefs about consequences;<br><br>Emotions	Reflective Motivation<br><br>Automatic motivation	Education<br><br>Persuasion	5.1 information about health consequences<br><br>5.6 information about emotional consequences
Perception of **skin testing as less frightening** than the oral challenge test [QI]	Pre-test intervention booklet	• Provide information about the oral challenge test; Provide reassurance that the length of time spent at the clinic is long enough to check for any delayed reactions	Beliefs about consequences;<br><br>Emotions	Reflective Motivation<br><br>Automatic motivation	Education<br><br>Persuasion	2.7 feedback on outcomes of behaviour<br><br>5.1 information about health consequences
Patients **concerned about how they would be monitored** during the allergy test and whether doctors would respond quickly in case of reaction [QI]	Pre-test intervention booklet<br><br>Trial PIS	• Provide information on how doctors and nurses will monitor patients during the skin testing and the oral challenge	Beliefs about consequences;<br><br>Emotions	Reflective Motivation<br><br>Automatic motivation	Education<br><br>Persuasion	5.1 information about health consequences
**Worries about taking penicillin at home** following the allergy test in clinic and whether there is someone to call in case of reaction [QI]	Pre-test intervention booklet	• Provide information on how the nurse will contact the patients after they have finished taking the penicillin at home to check whether they had any symptoms;<br>• Provide information of possible side effects when taking penicillin at home and that they are mild and do not require treatment<br>• Provide contact details of who to call at the clinic once they experience side effects at home	Beliefs about consequences<br><br>Emotions	Reflective Motivation<br><br>Automatic motivation	Education<br><br>Persuasion	5.1 information about health consequences

For peer review only

Barriers / *facilitators* to target behaviours	Intervention Component/s	Intervention ingredient	Theoretical Domains Framework (TDF)	Target construct (BCW)	Intervention function (BCW)	Behaviour Change Technique (using 93 BCT taxonomy v1)
Perception that the procedure is **over medicalised** [QI]	Pre-test intervention booklet;<br><br>PAT procedures at allergy clinic	• Provide information on the test procedures and on how only the necessary tests will be taken: some of the stages will be skipped based on previous history of reactions and on reaction to the skin test (only necessary tests are carried out)	Beliefs about consequences<br><br>Knowledge	Reflective Motivation	Education<br><br>Persuasion	5.1 information about health consequences
**Lack of knowledge** that allergy status could change over time [RR]	Pre-test intervention booklet	• Provide information about consequences of incorrect allergy label and the benefit of taking the test	Knowledge	Psychological capability	Education	4.2 information about antecedents
Primary care providers **did not inform them about the availability** of PAT [RR]	Consultations with clinicians<br><br>Invitation letter/Trial PIS	• Provide information about penicillin allergy labels and PAT	Knowledge	Psychological capability	Incentivisation<br><br>Education	2.7 feedback on outcome of behaviour
Always having had a label of penicillin allergy [QI]	Pre-test intervention booklet	• Provide information that once the allergy label is on the patient's medical record is rarely questioned and difficult to remove<br>• Provide information about the percentages of patients who really have a penicillin allergy and the reasons of incorrect allergy label.	Behavioural regulation<br><br>Beliefs about capabilities	Psychological capability<br><br>Reflective Motivation	Education<br><br>Persuasion	5.1 beliefs about health consequences
*Previous **experience of negative consequences** of having a penicillin allergy label and intentions to take penicillin [QI]*	Consultations with clinicians	• Provide an opportunity for patients to discuss with clinicians their previous medical history of penicillin allergy reactions and highlight their personal motivations to undertake the test	Emotion	Automatic motivation	Incentivisation	2.7 feedback on outcomes of behaviour

**Barriers / *facilitators* to target behaviours**	**Intervention Component/s**	**Intervention ingredient**	**Theoretical Domains Framework (TDF)**	**Target construct (BCW)**	**Intervention function (BCW)**	**Behaviour Change Technique (using 93 BCT taxonomy v1)**
*Patient **awareness that penicillin is a first-line treatment** for many infections [QI]*	Pre-test intervention booklet<br><br>Trial PIS	• Provide evidence on benefits of taking penicillin compared to broad spectrum antibiotics	Knowledge<br><br>Beliefs about consequences	Psychological capability<br><br>Reflective Motivation	Education	2.7 feedback on outcome of behaviour
*Feeling **relieved because allergy status has been confirmed** [QI]*	Pre-test booklet	• Provide information about the accuracy of allergy labels	Reinforcement	Automatic motivation	Incentivisation	2.7 feedback on outcome of behaviour
*Diagnosis is **an emotionally difficult event** and relief in getting an answer [QI]*	Consultations with clinicians<br><br>Post-test intervention booklet	• Provide information on how they will get the results of the testing and provide opportunity to discuss it with GPs<br>• Provide information on the meaning on a negative test, who to inform about the test result and how to do so	Emotions	Automatic motivation	Persuasion	5.3 information about social and environmental consequences
*Assurance of **access to trained medical staff** at the time of the test [QI]*	Pre-test intervention booklet	• Provide reassurance that all medical staff are fully trained and will act immediately in case of allergy reaction	Beliefs about consequences;<br><br>Emotions	Reflective Motivation<br><br>Automatic motivation	Education<br><br>Persuasion	5.1 information about health consequences
*Perception of being **properly monitored** and of feeling safe during the test [QI]*	Pre-test intervention booklet;<br><br>PAT procedures at allergy clinic<br><br>Trial PIS	• Provide reassurance that the medical staff will monitor the patients during the test and will follow up after the patients will have finished the course at home	Beliefs about consequences<br><br>Emotions	Reflective Motivation<br><br>Automatic motivation	Education<br><br>Persuasion	5.3 information about social and environmental consequences

For peer review only

**Barriers / *facilitators* to target behaviours**	**Intervention Component/s**	**Intervention ingredient**	**Theoretical Domains Framework (TDF)**	**Target construct (BCW)**	**Intervention function (BCW)**	**Behaviour Change Technique (using 93 BCT taxonomy v1)**
*Feeling **safe if the test takes place on hospital premises** [QI]*	Pre-test intervention booklet;<br><br>PAT procedures at allergy clinic<br><br>Trial PIS	• Provide information on where the allergy clinic is.<br>• Attending the PAT at the allergy clinic at the hospital	Knowledge	Psychological capability	Education	2.7 feedback on outcomes of behaviour
*Clear **expectations of what the procedure involves?** [QI]*	Consultations with clinicians;<br><br>Pre-test intervention booklet<br><br>Trial PIS	• Provide information about the procedures of skin testing and oral challenge test	Knowledge	Psychological capability	Education	2.7 feedback on outcomes of behaviour<br><br>4.2 information about antecedents
*Opportunity for **addressing any concerns** about the procedure [QI]*	Consultations with clinicians	• Provide the opportunity to discuss any concerns about taking the test	Environmental Context and resources;<br><br>Emotions	Physical opportunity<br><br>Automatic motivation	Enablement	12.2 restructuring of social environment
*Belief that PAT provides **valuable information** [RR]*	PAT procedures at allergy clinic;<br><br>Post-test intervention booklet	• Provide information about penicillin allergy label<br>• Provide information about the meaning of negative test result and the safety of taking penicillin	Beliefs about consequences	Reflective motivation	Incentivisation	2.7 feedback on outcomes of behaviour

For peer review only

**Barriers / *facilitators* to target behaviours**	**Intervention Component/s**	**Intervention ingredient**	**Theoretical Domains Framework (TDF)**	**Target construct (BCW)**	**Intervention function (BCW)**	**Behaviour Change Technique (using 93 BCT taxonomy v1)**
***Interest** in being tested [RR]*	PAT procedures at allergy clinic<br><br>Pre-test intervention booklet	• Provide information about the benefit of taking the test	Reinforcement	Automatic motivation	Incentivisation	2.7 feedback on outcomes of behaviour
***Benefits** of the negative test results [QI]*	Pre-test intervention booklet	• Provide information about the benefits of having a negative test results (being able to take penicillin, being less worries about penicillin, not having to go back to the GP with a reaction)	Reinforcement	Automatic motivation	Incentivisation	2.7 feedback on outcome of behaviour
***Importance of knowing** that the penicillin allergy is correct and that the penicillin allergy test is positive [*QI]*	Consultations with clinicians;<br><br>Invitation letter/Trial PIS	• Provide information about the benefits of accurate allergy labels	Reinforcement	Automatic motivation	Incentivisation	2.7 feedback on outcome of behaviour
**Target behaviour: Patient consumption of penicillin when prescribed first line**						
**Perceived risk of clinicians' not prescribing** penicillin even after a negative result [QI]	Post-test allergy booklet<br><br>Post-test intervention card	• Provide a card which will inform the clinicians of the change in allergy label<br>• Provide information on how the GP will be informed of the allergy test result	Social Influences	Social opportunities	Enablement	3.2 social support (practical)<br><br>12.2 restructuring of social environment

For peer review only

**Barriers / *facilitators* to target behaviours**	**Intervention Component/s**	**Intervention ingredient**	**Theoretical Domains Framework (TDF)**	**Target construct (BCW)**	**Intervention function (BCW)**	**Behaviour Change Technique (using 93 BCT taxonomy v1)**
**Perceived risk of Re-labelling** of patients allergy even after negative test result [QI]	Post-test allergy booklet<br><br>Post-test intervention card, allergy test result letter	Provide information on how the GP will update the medical record of the patient and other medical systems with negative test results	Social influences	Social opportunities	Enablement	3.2 social support (practical)
Perception of test as **not accurate** [QI, RR]	Post-test allergy booklet	Provide information that the test was very accurate in identifying whether the patients have a true penicillin allergy	Beliefs about consequences	Reflective motivation	Education<br><br>Persuasion	5.1 information about health consequences
**Severity** of past allergy reactions [QI]	PAT procedures at allergy clinic	Provide information that nurse or doctors at allergy clinic will only test patient who are perceived as low risk	Beliefs about consequences<br><br>Knowledge	Reflective motivation<br><br>Psychological capability	Education<br><br>Persuasion	4.3 information about antecedents
**Patient Belief that they are still allergic** to penicillin after a negative text result [RR]	Post-test allergy booklet	Provide information about the reliability of the test	Beliefs about consequences	Reflective motivation	Education<br><br>Persuasion	5.1 beliefs about consequences
**Fear of having an allergic reaction** again [RR]	Post-test allergy booklet	Provide information about low risks of having an allergic reaction in the future	Emotion	Automatic Motivation	Education<br><br>Persuasion	5.6 beliefs about emotional consequences
**Lack of confidence** that penicillin could be safely administered [RR]	Post-test intervention booklet	Provide information about the meaning of test results and which antibiotics are now safe to use	Beliefs about consequences	Reflective motivation	Education<br><br>Persuasion	5.1 beliefs about consequences
**Lack of knowledge of which antibiotics they could take** safely [RR]	Post-test intervention booklet	Provide a list of antibiotics that patients with a negative test result can take	Knowledge	Psychological capability	Education	4.2 information about antecedents

**Barriers / *facilitators* to target behaviours**	**Intervention Component/s**	**Intervention ingredient**	**Theoretical Domains Framework (TDF)**	**Target construct (BCW)**	**Intervention function (BCW)**	**Behaviour Change Technique (using 93 BCT taxonomy v1)**
**Distrust in health care providers'** advice if they don't prescribe penicillin even after the negative test result [RR]	Post-test intervention card	Provide card to show to health care professionals which gives details about the allergy test completed and explains that the patient had a negative test result	Reinforcement	Automatic motivation	Environmental restructuring	12.2 restructuring of social environment<br><br>12.5 adding objects to the environment
**Lack of knowledge about how to interpret skin test results** [RR]	Post-test intervention card	Provide explanation of the meaning of negative test results and what to do the next time they will need antibiotics	Knowledge	Psychological capability	Education	4.2 information about antecedents
**No differences in concerns** between patients who were investigated through skin test or oral test [RR]	Post-test intervention booklet	Provide information about the accuracy of the test completed	Beliefs about consequences	Reflective motivation	Education<br><br>Persuasion	5.1 beliefs about consequences
*Perception of the test as **definitive answer** and proof of allergy status [QI]*	PAT procedures at allergy clinic;<br><br>Post-test intervention booklet and card, allergy test result letter	• Provide the test results from the allergy clinic<br>• Provide information on the meaning of the negative test result and information about the accuracy of the test<br>Provide information about reliability of test	Reinforcement	Automatic motivation	Environmental restructuring	2.7 feedback on outcomes of behaviour
*Perception to have undergone a **thorough testing procedure** [QI]*	PAT procedures at allergy clinic	Monitoring of the patients by the nurse or doctor at allergy clinic who will provide medical assistance when needed	Reinforcement	Automatic motivation	Environmental restructuring	12.2 restructuring of the social environment

**Barriers / *facilitators* to target behaviours**	**Intervention Component/s**	**Intervention ingredient**	**Theoretical Domains Framework (TDF)**	**Target construct (BCW)**	**Intervention function (BCW)**	**Behaviour Change Technique (using 93 BCT taxonomy v1)**
***Lack of reaction** following the test [QI]*	PAT procedures at allergy clinic<br><br>Pre-test intervention booklet	Provide information about the type of reactions and their indication on the state of the allergy	Reinforcement	Automatic motivation	Environmental restructuring	12.1 restructuring of the physical environment
**Target behaviour: Clinician referral of low risks patients to Penicillin Allergy Testing**						
**Lack of knowledge** about incidence of **incorrect penicillin allergy labels** [QI]	Information for general practice booklet<br><br>Training	• Provide information on penicillin allergy records and its implications	Knowledge	Psychological capability	Education	4.2 information about antecedents
**Lack of knowledge** of how **accurate** the tests are [QI]	Information for general practice booklet	• Provide information that the oral challenge test is the gold standard test for penicillin allergy	Knowledge	Psychological capability	Education	4.2 information about antecedents
**Lack of knowledge** of the existence of **allergy services** or what they involve [QI]	Information for general practice booklet<br><br>Training	• Provide information what the penicillin allergy test involves and where it will take place	Knowledge	Psychological capability	Education	4.2 information about antecedents
**Poor experience** of PAT [QI, RR]	Information for general practice booklet	• Provide information about why it is important to have correct penicillin allergy labels and what the test involve	Skills<br><br>Behavioural regulation	Physical capability<br><br>Psychological Capability	Training<br><br>Education<br><br>Persuasion	2.7 feedback on outcome of behaviour
**Lack of understanding** PAT procedures and results [QI, RR]	Information for general practice booklet<br><br>Training	• Provide information about the three stages of penicillin allergy testing and what negative and positive results mean	Knowledge	Psychological capability	Education	4.2 information about antecedents

For peer review only

**Barriers / *facilitators* to target behaviours**	**Intervention Component/s**	**Intervention ingredient**	**Theoretical Domains Framework (TDF)**	**Target construct (BCW)**	**Intervention function (BCW)**	**Behaviour Change Technique (using 93 BCT taxonomy v1)**
**Lack of guidelines** in learning about **benefits and risks** of allergy services [QI, RR]	Information for general practice booklet	• Provide a series of references around penicillin allergy and PAT, including NICE guidelines	Knowledge	Psychological capability	Education	9.1 credible source
**Lack of guidance on referral** [QI, RR]	Information for general practice booklet<br><br>Training and WIs	• Provide information about the NICE guidelines<br>• Provide a system to refer low risks patients to do testing	Knowledge<br><br>Skills	Psychological capability<br><br>Physical skills	Education<br><br>Training	1.4 action planning<br><br>4.1 instructions on how to perform a behaviour
**Lack of unified approach** for referral of patients to PAT [RR]	Information for general practice booklet<br><br>Training and WIs	• Provide references on NICE guidelines on referral<br>• Provide a system to refer low risks patients to do testing	Skills<br><br>Social influences	Physical skills<br><br>Social opportunity	Training<br><br>Modelling	1.4 action planning<br><br>4.1 instructions on how to perform a behaviour
**Lack of knowledge** regarding the **oral challenge test** [QI]	Information for general practice booklet<br><br>Training	• Provide information about the oral challenge test (stage 3 or PAT), why it is done, what the procedure is and how accurate it is	Knowledge	Psychological capability	Education	4.2 information about antecedents
Perception of **overburdening the allergy service** [QI]	Information for general practice booklet	• Provide information around NICE guidelines in support of PAT	Social/Professional role and identity<br><br>Knowledge	Social opportunity<br><br>Psychological capability	Modelling<br><br>Enablement<br><br>Training	9.1 credible source<br><br>6.3 information about others' approval

For peer review only

**Barriers / *facilitators* to target behaviours**	**Intervention Component/s**	**Intervention ingredient**	**Theoretical Domains Framework (TDF)**	**Target construct (BCW)**	**Intervention function (BCW)**	**Behaviour Change Technique (using 93 BCT taxonomy v1)**
**Lack of follow up** after referral [QI]	Pop-up alert<br><br>Information for general practice booklet<br><br>Training	• Provide information that they will receive confirmation about the patients' results	Environmental Context and resources	Physical opportunity	Environmental restructuring<br><br>Enablement	7.1 prompts/cues<br><br>2.7 feedback on outcome of behaviour
Expectations that **patients may not want to be tested** [RR]	Consultations with patients<br><br>Pre-test intervention booklet	• Provide pre-test booklet to discuss with patients<br>• Provide the opportunity to discuss the benefits of the test during the consultation	Social influences	Social opportunities	Enablement	3.2 social support (practical)<br><br>21.2 restructuring of social environment
Concerns that the test might be **expensive** [QI]	PAT procedures at clinic	• Provide support to referral of patients from colleagues and practice managers<br>• Provide the PAT testing procedures	Social/Professional role and identity	Social opportunities	Enablement<br><br>Modelling	3.2 social support (practical)
Fear of **getting it wrong** and the safety patients [QI]	Training<br><br>PAT procedures in clinic	• Provide support to refer low risk patients to do PAT<br>• Provide evidence of safety of PAT	Social/Professional role and identity<br><br>Beliefs about consequences<br><br>Emotion	Social opportunities<br><br>Reflective motivation<br><br>Automatic motivation	Enablement<br><br>Modelling<br><br>Education<br><br>Persuasion	5.1 information about health consequences<br><br>5.6 information about emotional consequences

For peer review only

**Barriers / *facilitators* to target behaviours**	**Intervention Component/s**	**Intervention ingredient**	**Theoretical Domains Framework (TDF)**	**Target construct (BCW)**	**Intervention function (BCW)**	**Behaviour Change Technique (using 93 BCT taxonomy v1)**
Perception that it is **safer to take patients' word for it** [QI]	Information for general practice booklet	• Provide evidence that only a small percentage of patients who think to be allergic actually are	Beliefs about consequences<br><br>Emotions	Reflective motivation<br><br>Automatic motivation	Education<br><br>Persuasion	5.1 information about health consequences
Fear of **getting it wrong** and **professional ramifications** [*QI]	Training<br><br>PAT procedures in clinic	• Provide support to refer low risk patients to do PAT	Social/Professional role and identity<br><br>Environmental context and resources<br><br>Emotion	Social opportunities<br><br>Physical opportunity<br><br>Automatic motivation	Enablement<br><br>Modelling	3.2 social support (practical)
**Lack of time** during consultation to discuss referral [RR]	Consultation with patients<br><br>Training	• Provide the opportunity to have a consultation with patients to specifically discuss PAT	Environmental context and resources<br><br>Beliefs about capabilities	Physical opportunities<br><br>Reflective motivation	Enablement	12.2 restructuring of social environment
Anticipation that **patients will not want** to have a reaction [RR]	Consultations with patients<br><br>Pre-test intervention booklet	• Provide the opportunity to discuss with patients the risks of taking the test<br>• Provide pre-test booklet with information around the risks of PAT	Social influences	Social opportunity	Enablement	12.2 restructuring of social environment
**Forgetting to discuss** testing [RR]	Consultations with patients<br><br>Training	• Provide the opportunity to have consultations specifically around PAT	Behavioural regulation	Psychological capability	Enablement	3.2 social support (practical)
**Not knowing** that patients had an **allergy** [RR]	Consultations with patients	• Provide the opportunity to discuss their medical history with patients	Knowledge	Psychological capability	Training	2.7 feedback on outcomes of behaviour

For peer review only

**Barriers / *facilitators* to target behaviours**	**Intervention Component/s**	**Intervention ingredient**	**Theoretical Domains Framework (TDF)**	**Target construct (BCW)**	**Intervention function (BCW)**	**Behaviour Change Technique (using 93 BCT taxonomy v1)**
**Lack of access** to testing [RR]	PAT testing procedures in clinic<br><br>Information for general practice booklet	• Provide the information that they can refer low risks patients to undertake PAT<br>• Provide information about where the test will be and what the stages are	Environmental Context and resources<br><br>Knowledge	Physical opportunity<br><br>Psychological capability	Enablement<br><br>Training	5.3 information of social and environmental consequences<br><br>15.1 verbal persuasion of capabilities
**Lack of ability in drawing a conclusion** on allergy status from medical history [RR]	Information for general practice booklet	• Provide information about the differences between drug reaction and allergy	Knowledge	Psychological capability	Training	4.2 information about antecedents
**Limited knowledge** of drug **allergies** [QI, RR]	Information for general practice booklet	• Provide information on how an allergy reaction is different than drug reaction	Knowledge	Psychological capability	Training	4.2 information about antecedents
**Poor knowledge** of allergy skin **testing** [RR]	Information for general practice booklet	• Provide information about skin testing (stage 2 of PAT), what the test will include, how patients will be monitored and how the results will be interpreted in relation to stage 3.	Knowledge	Psychological capability	Training	2.7 feedback on outcome of behaviour
**Lack of confidence** in dealing with allergies [RR]	Information for general practice booklet	• Provide information about penicillin allergy records and its implication	Beliefs about capabilities	Reflective motivation	Education<br><br>Persuasion	5.1 information about health consequences
Ready **availability of alternate antibiotics** [RR]	Information for general practice booklet	• Provide information on standards of care and use of broad-spectrum antibiotics	Beliefs about consequences	Reflective motivation	Education<br><br>Persuasion	5.1 information about health consequences
Awareness that **CCG are "cash strapped"** [QI]	PAT procedures at clinic	• Provide allergy testing at the hospital	Environmental context and resources	Environmental Context and resources	Physical opportunity<br><br>Enablement	12.2 restructuring of social environment

For peer review only

**Barriers / *facilitators* to target behaviours**	**Intervention Component/s**	**Intervention ingredient**	**Theoretical Domains Framework (TDF)**	**Target construct (BCW)**	**Intervention function (BCW)**	**Behaviour Change Technique (using 93 BCT taxonomy v1)**
Worries about **costs of tests** [*QI]	PAT procedures at clinic	• Provide support in referring patients to PAT	Environmental context and resources	Environmental Context and resources	Physical opportunity<br><br>Enablement	12.2 restructuring of social environment
**Referral takes time** [QI]	PAT procedures at clinic<br>Training and WIs	• Provide guidance about referral and time of responses	Environmental context and resources	Environmental Context and resources	Physical opportunity<br><br>Enablement	4.1 instructions on how to perform a behaviour
**Patients not aware that the allergy label** may have a negative consequences [QI]	Consultations with patients	• Provide the opportunity to discuss the effects of inaccurate allergy labels	Social Influences	Social opportunity	Enablement	12.2 restructuring of social environment
Lack **of support from colleagues** in changing the label [QI]	Training and WIs	• Provide support from lead GPs and colleagues about changing the labels	Social influences	Social opportunity	Modelling	3.2 social support (practical)
**Experience of prescribing an alternative antibiotic** as routine [QI]	Training<br><br>Information for general practice booklet	• Provide information about how to refer low risks patients<br>• Provide information on the benefits of taking penicillin	Behavioural regulation	Psychological capability	Enablement	4.1 instruction on how to perform a behaviour
*Perception of how **easy finding an alternative antibiotic** is [QI]*	Information for general practice booklet	• Provide information about the benefits of taking penicillin<br>• Provide information about the effects of broad spectrum antibiotics	Knowledge	Psychological capability	Education<br><br>Enablement	5.1 information about health consequences
*Awareness that patients have been **historically over diagnosed** with penicillin allergy [QI]*	Information for general practice booklet<br><br>Training	• Provide information that incorrect penicillin allergy records are very common	Knowledge<br><br>Memory	Psychological capability	Education<br><br>Training	4.2 information about antecedents

**Barriers / *facilitators* to target behaviours**	**Intervention Component/s**	**Intervention ingredient**	**Theoretical Domains Framework (TDF)**	**Target construct (BCW)**	**Intervention function (BCW)**	**Behaviour Change Technique (using 93 BCT taxonomy v1)**
*Awareness that **records might not be true** because patients do not remember any details of the allergy [QI]*	Information for general practice booklet	• Provide information about the reasons of incorrect penicillin allergy labels	Beliefs about capability	Reflective motivation	Education	4.2 information about antecedents
*Perception of **importance of referral** and penicillin allergy becoming more on the radar [RR]*	Information for general practice booklet<br><br>Training	• Provide information of the benefit of PAT and appropriate allergy labels	Reinforcement<br><br>Beliefs about consequences<br><br>Memory<br><br>Environmental context and resources	Automatic motivation<br><br>Reflective motivation<br><br>Physical opportunities	Incentivisation<br><br>Education<br><br>Persuasion<br><br>Training	5.1 information about health consequences
***Previous medical** and reaction **history** of patients [QI]*	Consultation with patients	• Provide the opportunity to discuss with patients their medical history and why the test will be relevant for them	Beliefs about capabilities	Reflective motivation	Enablement	12.2 restructuring of social environment
*Awareness that it is **important to prescribe** the most suitable antibiotic [QI]*	Information for general practice booklet<br><br>Training	• Provide information about the benefit of taking penicillin and about antimicrobial resistance	Reinforcement<br><br>Memory	Automatic Motivation<br><br>Psychological capability	Incentivisation<br><br>Training	4.2 information about antecedents

For peer review only

**Barriers / *facilitators* to target behaviours**	**Intervention Component/s**	**Intervention ingredient**	**Theoretical Domains Framework (TDF)**	**Target construct (BCW)**	**Intervention function (BCW)**	**Behaviour Change Technique (using 93 BCT taxonomy v1)**
*Belief that **testing would lead to removal** of the allergy label [RR]*	Information for general practice booklet	• Provide information about how to update the patients records after receiving a negative test results<br>• Provide information about prescribing penicillin after a negative test	Reinforcement	Automatic motivation	Incentivisation	1.1 goal setting<br><br>1.4 action planning.<br><br>4.1 instructions on how to perform a behaviour
*Belief of **benefits of removing** the allergy label [RR]*	Information for general practice booklet	• Provide information about how incorrect penicillin allergy labels can be harmful for patients	Reinforcement<br><br>Beliefs about consequences	Automatic motivation<br><br>Reflective motivation	Incentivisation<br><br>Education<br><br>Persuasion	5.1 information about health consequences
***Previous experience of referring patients** to PAT [QI]*	Consultations with patients<br><br>PAT procedures in clinic	• Provide the opportunity to explain to patients about PAT procedures and results<br>• Provide the PAT service	Beliefs about capabilities	Reflective motivation	Enablement	15.1 verbal persuasion about capability
***Warning patients** about side effects at the time of prescription [QI]*	Consultations with patients<br><br>Pre-test intervention booklet	• Provide the opportunity to discuss any side effects with the patients	Social/Professional role and identity<br><br>Beliefs about capabilities	Social opportunities<br><br>Reflective motivation	Enablement	12.2 restructuring of social environment
***NICE guidelines on importance of PAT** and criteria for selecting patients for referral [LR]*	Information for general practice booklet<br><br>Training	• Provide information on the NICE guidelines on referring to PAT	Knowledge<br><br>Social influences	Psychological Capability<br><br>Social opportunity	Training<br><br>Enablement	9.1 credible source

For peer review only

Barriers / *facilitators* to target behaviours	Intervention Component/s	Intervention ingredient	Theoretical Domains Framework (TDF)	Target construct (BCW)	Intervention function (BCW)	Behaviour Change Technique (using 93 BCT taxonomy v1)
*Belief that **penicillin allergy is an important** issue [RR]*	Information for general practice booklet	• Provide information on incorrect allergy labels, adverse patient outcomes, effects on antimicrobial prescribing, and on antimicrobial resistance	Reinforcement	Automatic motivation	Education<br><br>Persuasion	5.1 information about health consequences
*Perception of **support of CCG** [QI]*	Information for general practice booklet	• Provide information about the support from colleagues and guidelines for referral	Professional Role	Reflective motivation	Persuasion	9.1 Credible source
***Previous experience of removing** or challenging the records *QI]*	PAT procedures at clinic	• Provide the opportunity to refer patients to ultimately change inaccurate labels	Behavioural regulation	Psychological capability	Enablement	15.1 verbal persuasion of capability
**Target behaviour: Clinician prescription of penicillin after negative test result**						
Doubts on **accuracy of allergy labels** in medical records [QI]	Information for general practice booklet	• Provide information that the oral challenge is the gold standard test for penicillin allergy	Knowledge	Psychological capability	Education<br><br>Persuasion	4.2 information about antecedents
**Worries of harming patients** [QI, RR]	Information for general practice booklet	• Provide information about safety of patients having penicillin after the test	Emotions<br><br>Beliefs about consequences	Automatic motivation<br><br>Reflective motivation	Education<br><br>Persuasion	5.1 information about health consequences<br><br>5.6 information about emotional consequences
**Lack of knowledge on penicillin** [QI, RR]	Information for general practice booklet	• Provide information about penicillin allergy and effects on standard of care	Memory	Psychological capability	Training	2.7 feedback on outcomes of behaviour
**System did not distinguish between intolerances and**	Pop-up alert WIs and allergy test results letter	• Provide information on the change of allergy status	Environmental context and resources	Physical opportunity	Environmental restructuring	12.5 adding objects to the environment

For peer review only

Barriers / *facilitators* to target behaviours	Intervention Component/s	Intervention ingredient	Theoretical Domains Framework (TDF)	Target construct (BCW)	Intervention function (BCW)	Behaviour Change Technique (using 93 BCT taxonomy v1)
**allergies** and may still show alerts [QI]			Beliefs about capabilities	Reflective motivation	Enablement	
**Responsibility of changing the system** given to allergy clinic and lack of clarity on how to change them[QI]	Pop-up alert<br><br>Training, WIs and allergy test results letter	• Provide information on who will be responsible in changing the record	Social/professional role and identity<br><br>Beliefs about consequences<br><br>Behavioural regulation	Social opportunity<br><br>Reflective motivation<br><br>Psychological capability	Environmental restructuring<br><br>Enablement	4.1 instructions on how to perform a behaviour
**Lack of confidence** that penicillin could be safely administered [RR]	Information for general practice booklet	• Provide information about safety of taking penicillin when the test result is negative	Beliefs about consequences	Reflective motivation	Education persuasion	5.1 beliefs about health consequences
**Lack of guidance** on how to interpret the results [QI]	Information for general practice booklet<br><br>Training, WIs, allergy test results letter	• Provide information on how the results will be presented to the clinicians and patients	Knowledge<br><br>Skills	Psychological capability<br><br>Cognitive skills	Education<br><br>Persuasion<br><br>Training	4.1 instructions on how to perform a behaviour<br><br>2.7 feedback on outcome of behaviour
Perception that **anxious patients may still not want to take penicillin,** and they would believe other people's advice instead [QI]	Consultation with patients<br><br>Pre-test booklet	• Provide the opportunity to discuss the accuracy of the results with patients	Beliefs about capability<br><br>Reinforcement<br><br>Social influences<br><br>Behavioural regulation	Reflective motivation<br><br>Automatic motivation<br><br>Social opportunity	Enablement<br><br>Incentivisation	12.2 restructuring of social environment

For peer review only

**Barriers / *facilitators* to target behaviours**	**Intervention Component/s**	**Intervention ingredient**	**Theoretical Domains Framework (TDF)**	**Target construct (BCW)**	**Intervention function (BCW)**	**Behaviour Change Technique (using 93 BCT taxonomy v1)**
				Psychological capability		
**Lack of follow** up of the results [QI]	Pop-up alert<br>Allergy test results letter	• Provide an alert when the results are available and the indication of a change of allergy label	Beliefs about consequences<br><br>Behavioural regulation	Reflective motivation<br><br>Psychological capability	Enablement<br><br>Environmental restructuring	12.4 adding object to the environment
Awareness **of different approaches in changing records** in different practices [QI]	Training , WIs, allergy test results letter	• Provide information of the new system in changing labels after PAT and how practices have been involved	Environmental context and resources<br><br>Behavioural regulation	Physical opportunity<br><br>Psychological capability	Environmental restructuring<br><br>Modelling	12.2 restructuring of the social environment<br><br>3.2 social support (practical)
Patients may worry less *about allergies [QI]*	Consultation with patients	• Provide the opportunity to reassure patients	Social influences	Social opportunity	Environmental restructuring	12.2 restructuring of social environment
*Recognition that **test provided evidence** that patient is not allergic [QI]*	Information for general practice booklet<br><br>PAT procedures at clinic	• Provide information about the accuracy of the test and who will perform it	Belief about consequences	Reflective motivation	Education<br><br>Persuasion	5.1 information about health consequences
*Awareness that the **test will allow prescribing first line** antibiotics and fight antibiotics resistance [QI]*	Information for general practice booklet	• Provide information about the effects of accurate penicillin allergy labels	Reinforcement	Automatic motivation	Incentivisation	2.7 feedback on outcomes of behaviour
*Perception of **penicillin allergy testing to be specialist advice** and to be objective evidence[QI]*	Information for general practice booklet	• Provide information of who will perform the test and interpret the results	Belief about consequences<br><br>Reinforcement	Reflective motivation<br><br>Automatic motivation	Education<br><br>Persuasion	5.1 information about consequences

For peer review only

**Barriers / *facilitators* to target behaviours**	**Intervention Component/s**	**Intervention ingredient**	**Theoretical Domains Framework (TDF)**	**Target construct (BCW)**	**Intervention function (BCW)**	**Behaviour Change Technique (using 93 BCT taxonomy v1)**
Discussion of the negative test results *to address patients concerns about taking penicillin [QI]*	Consultations with patients	• Provide opportunity to discuss results with patients	Beliefs about capabilities<br><br>Social influences	Reflective motivation<br><br>Social opportunity	Enablement	5.1 beliefs about emotional consequences
Previous experience *of removing or challenging the records [QI]*	WIs, allergy test results letter	• Provide information about how to remove labels	Skills	Cognitive skills	Training	4.1 instructions on how to perform a behaviour
Perception that ***the system is easy to change*** *[QI]*	Pop-up alert, WIs and allergy test results letter	• Provide information about the change of label in the system	Beliefs about capabilities<br><br>Environmental context and resources	Reflective motivation<br><br>Physical opportunity	Enablement<br><br>Environmental restructuring	12.5 adding objects to the environment
Intention to change the label *[QI]*	Training, allergy test results letter	• Provide support and instructions on changing the label	Reinforcement	Automatic motivation	Incentive	2.7 feedback on outcomes of behaviour

S2 Table of changes: “Penicillin Allergy Testing: going for a test” booklet

Suggested changes	Changes made	Reasons for change	Priority for change
**Front page**			
The image on the cover of the booklet must be changed as it does not relate to what happens during penicillin allergy testing; also the blood samples on the GP desk look scary.	A new image was selected showing a more friendly looking consultation between patient and GP without any medical equipment	EO	Must do

Suggested changes	Changes made	Reasons for change	Priority for change
The logos could be put at the bottom of the cover pager (rather than at the top) as they distract from the image)	The logos are on the bottom of the page	EO	Could do
The participants suggested making the booklets even easier to follow by adding text boxes throughout to separate big chunks of text.	The booklet was made more visually appealing	EO	Could do
**How can this booklet help me?**			
The contents page looks a bit dense on the page. The participants did not like the "chemical symbols" image in the background design.	• Better use of the space on the contents page was made and the text more was spread across the page.<br>• The introduction to the booklet was put in a separate box to make it look more manageable. Chemical symbols image were removed.	EO	Should do
**How do I know if I am allergic to penicillin?**			
The participants suggested putting the last sentence of this section: "A penicillin allergy test is the safest way to find out whether or not you are allergic to penicillin" in bold.	The last sentence was put in bold	EO, QI	Must do

For peer review only

Suggested changes	Changes made	Reasons for change	Priority for change
Statistics about the prevalence of allergy were not always understood by the participants, as the participants often thought that 1 in 10 people are allergic. They wanted a more visual presentation of this key information	An infographic was created showing how many people think they are allergic and how many people actually are allergic. The image of a GP taking blood pressure was replaced by the infographic.	EO	Should do
This section looks also a bit dense on the page	The text was spread across to the following page	EO	Should do
**Why is it important for people to have a penicillin allergy test?**			
Participants were unsure what narrow and broad spectrum antibiotics were and did not recognise MRSA abbreviation.	• The reference to narrow and broad spectrum antibiotics was removed<br>• The full name for MRSA was presented	RT, CT	Must do
Participants did not always know that penicillin is more than one antibiotic. This message should be made clearer.	An additional sentence was added to provide information that penicillin includes a number of different antibiotics	EO, QI	Must do
Booklet 2 mentioned that penicillin is good at treating throat infections- this information was missing from this booklet.	A sentence was added to explain that “antibiotics are very useful to treat many infections, including ear, skin, throat and chest infections”	EO, QI	Must do
The information was slightly repetitive and perhaps the section should start with the strongest message (which participants	• The section was made more concise.<br>• The first paragraph includes information on what	EO	Should do

For peer review only

Suggested changes	Changes made	Reasons for change	Priority for change
thought was that penicillin are effective).	penicillins are, what they are used for, and that they are considered the best treatment for infections		
Other sentences in this section, for example: "other antibiotics may not be as effective" could also be put in bold.	The sentence was put in bold	EO, QI	Should do
The section looks a bit dense.	The text was spread across the previous page	EO	Should do
**What does a penicillin allergy test involve?**			
Participants wanted to know if they can call the allergy clinic and whether they will be provided a number and what details the doctor or nurse will be checking (Why they would not have to be checked in person?).	It reads: "you will be provided with details of who to call if you have any symptoms or concerns"	RT, CT, QI	Must do
Participants wanted to have reassurance that 3 days would be enough to detect delayed reactions.	It reads: "this is long enough to check for any delayed reactions"	RT, CT, QI	Must do
Participants suggested making it clearer that the clinic is in the hospital.	This sections starts with "Penicillin allergy testing takes place in a hospital, in a specialist allergy clinic". The sentence was put in bold	RT, CT, QI	Must do

For peer review only

Suggested changes	Changes made	Reasons for change	Priority for change
Participants had queries about symptoms they could experience after three days of taking penicillin as the booklet said: “mild and mostly side effects”; they wanted clarification whether these could be allergic reaction symptoms	It reads: “these symptoms are generally mild and do not require treatment”	RT, CT, QI	Must do
Participants did not always like the phrase “like a mosquito bite” (skin testing stage) as some people may have an “unpleasant reaction to mosquitos”.	No change made – comment from one participant, felt by clinical team to be best way to describe and usual term used in current NHS leaflets.	RT, CT	Could do
There is a lot of text on one page	The descriptions of the test was spread over two pages	EO	Should do
A diagram alongside the stages of the test could be presented to make it even clearer	A diagram was provided for the three stages of testing and the description of the test was spread over two pages	EO	Could do
**Are there any risks to having the test?**			
The participants wanted to have a separate paragraph on what can happen during the test and what can happen during 3 days of taking penicillin at home.	There are two paragraphs, one on what could happen during the clinic visit, and one about what could happen at home	RT, CT	Must do
They wanted clarification whether the listed symptoms for taking penicillin at home: “sickness, a mild, small rash, nausea, upset stomach,	It reads: “Once you go home, it is possible you may develop mild symptoms such as sickness, a mild, small rash, nausea, upset stomach,	RT, CT, QI	Must do

For peer review only

Suggested changes	Changes made	Reasons for change	Priority for change
or other mild symptoms" were symptoms of an allergic reaction; this was especially confusing as feeling sick was also listed as part of the allergic reaction when taking penicillin in the clinic (you might feel sick, itchy, develop hives ('nettle rash'), swelling, a fast heartbeat, dizziness or difficulty breathing.	or other mild symptoms. It is extremely unlikely that you will have any serious symptoms, as these would have happened when you took penicillin during the clinic visit"		
The participants suggested putting the phrase: "extremely unlikely" in bold ("It is extremely unlikely that you will have any serious symptoms, as these would have happened when you took penicillin during the clinic visit".	The phrase was put in bold	EO	Could do

Note: EO: patient; RT: research team; CT: clinical team; QI: qualitative interviews

For peer review only

S3. Full description of the AlABAMA (ALlergy AntiBiotics And Microbial resistAnce) Intervention package in accordance with the Template for Intervention Description and Replication (TIDieR) framework.

- **1. BRIEF NAME:** AlABAMA (ALlergy AntiBiotics And Microbial resistAnce) Intervention package
- **2. WHY:** Penicillins are the most prescribed antibiotics and they are used as first-line therapy for many infections. Side effects and symptoms related to the infections are commonly mislabelled as allergy. Around 6% of people think that they are allergic to penicillin but fewer than 10% of these patients are truly allergic. As a consequence a significant proportion of the population have restricted access to highly effective penicillins, and are prescribed broad-spectrum antibiotics instead, which may be less effective and could increase the risks of acquiring multi-drug resistant bacteria. The goal of AlABAMA is to introduce a "pre-emptive" penicillin allergy assessment pathway (PAAP) for patients who are more likely to receive antibiotics that could impact antibiotic prescribing, have patients benefits, limit antimicrobial resistance (AMR) infections and deliver NHS cost savings. The AlABAMA approach will develop a "one stop shop" single Penicillin Allergy Test (PAT) at the hospital clinic for specialist immunology assessment (skin testing and/or oral challenge test).
- **3&4: WHAT:** AlABAMA is a complex behaviour change intervention which targets prescribers in general practice and low risk patients with a penicillin allergy record. See Table 2 for an overview of the key intervention components. For more details about the specific behaviour change techniques used, refer to the behavioural analysis.
- **5. WHO PROVIDED**: The AlABAMA research team will provide intervention materials to general practices taking part in the feasibility and main trial. Practices will be responsible for local implementation by the practice manager(s). GPs will have consultations with patients to check eligibility for entering the trial.
  Research nurses at the hospital will be responsible for sending the two intervention booklets to patients in the intervention arm by post. Research nurses at the immunology hospital clinic will deliver Stage 1, Stage 2 and/or stage 3 of the PAT.

For peer review only

- **6. HOW**: The intervention will be delivered mainly by written information for prescribers and for patients in the intervention arm. The site training will be delivered to all the GP leads and the practice manager(s) in one session per practice. Prescribers will receive information by email. Patients randomised to the PAAP intervention arm will receive the "Penicillin Allergy Testing: going for a test" by post. Patients will complete Stage 1 and Stage 2 (skin test) and /or Stage 3 (oral challenge test) in a single hospital visit which lasts around 3 hours. Patients with a negative PAT result will receive the "Penicillin Allergy Testing: a Negative Test Result" booklet by post. Prescribers will receive the update about the penicillin allergy test result by post and electronically.
- **7. WHERE**: AlABAMA is intended for implementation in general practices, where the site training and patient consultation also take place. The PAT takes place at the immunology clinic of a local hospital. Written information for clinicians is provided electronically subsequently to the site training. Written information for patients is sent by post by research nurses at the hospital.
- **8. WHEN and HOW MUCH**: Site training is provided by the AlABAMA research team prior to the screening and eligibility assessment of patients. Patients are invited to participate in the trial by a practice mail out. Once an expression of interest form had been received, patients attend a consultation with a clinician at their general practice. After confirming that they wish to take part in the trial patients will be contacted by a research nurse from the hospital by phone. The research nurse will randomise patients and book PAT appointments for those patients randomised to the trial arm. Patients randomised to the PAAP trial arm will be sent the "Penicillin Allergy Testing: going for a test" booklet with their hospital appointment letter. Patients randomised to the control arm will receive usual clinical care. All patients completing the PAT will receive a letter with the test result after their appointment. Patients who have tested negative will receive the "Penicillin Allergy Testing: a Negative Test Result" booklet and Intervention card by post. Practices will be informed of the test result and instructed to update the patients' electronic health record.

For peer review only

- **9. TAILORING**: The implementation of behavioural and clinical elements of the ALABAMA intervention package will be implemented in all GP practices and local immunology clinics participating in the trial.
- **10. MODIFICATIONS**: Modifications were made to the intervention materials following qualitative feedback from the feasibility study.
- **11-12. HOW WELL**: The intervention is being tested in a feasibility trial in 8 general practices. It will then be tested in a randomised control trial across 70 GP practices.

For peer review only

S2. Full description of the AlABAMA (ALlergy AntiBiotics And Microbial resistAnce) Intervention package in accordance with the Template for Intervention Description and Replication (TIDieR) framework.

- **1. BRIEF NAME:** AlABAMA (ALlergy AntiBiotics And Microbial resistAnce) Intervention package
- **2. WHY:** Penicillins are the most prescribed antibiotics and they are used as first-line therapy for many infections. Side effects and symptoms related to the infections are commonly mislabelled as allergy. Around 6% of people think that they are allergic to penicillin but fewer than 10% of these patients are truly allergic. As a consequence a significant proportion of the population have restricted access to highly effective penicillins, and are prescribed broad-spectrum antibiotics instead, which may be less effective and could increase the risks of acquiring multi-drug resistant bacteria. The goal of AlABAMA is to introduce a "pre-emptive" penicillin allergy assessment pathway (PAAP) for patients who are more likely to receive antibiotics that could impact antibiotic prescribing, have patients benefits, limit antimicrobial resistance (AMR) infections and deliver NHS cost savings. The AlABAMA approach will develop a "one stop shop" single Penicillin Allergy Test (PAT) at the hospital clinic for specialist immunology assessment (skin testing and/or oral challenge test).
- **3&4: WHAT:** AlABAMA is a complex behaviour change intervention which targets prescribers in general practice and low risk patients with a penicillin allergy record. See Table 2 for an overview of the key intervention components. For more details about the specific behaviour change techniques used, refer to the behavioural analysis.
- **5. WHO PROVIDED**: The AlABAMA research team will provide intervention materials to general practices taking part in the feasibility and main trial. Practices will be responsible for local implementation by the practice manager(s). GPs will have consultations with patients to check eligibility for entering the trial.
  Research nurses at the hospital will be responsible for sending the two intervention booklets to patients in the intervention arm by post. Research nurses at the immunology hospital clinic will deliver Stage 1, Stage 2 and/or stage 3 of the PAT.

For peer review only

- **6. HOW**: The intervention will be delivered mainly by written information for prescribers and for patients in the intervention arm. The site training will be delivered to all the GP leads and the practice manager(s) in one session per practice. Prescribers will receive information by email. Patients randomised to the PAAP intervention arm will receive the “Penicillin Allergy Testing: going for a test” by post. Patients will complete Stage 1 and Stage 2 (skin test) and /or Stage 3 (oral challenge test) in a single hospital visit which lasts around 3 hours. Patients with a negative PAT result will receive the “Penicillin Allergy Testing: a Negative Test Result” booklet by post. Prescribers will receive the update about the penicillin allergy test result by post and electronically.
- **7. WHERE**: AlABAMA is intended for implementation in general practices, where the site training and patient consultation also take place. The PAT takes place at the immunology clinic of a local hospital. Written information for clinicians is provided electronically subsequently to the site training. Written information for patients is sent by post by research nurses at the hospital.
- **8. WHEN and HOW MUCH**: Site training is provided by the AlABAMA research team prior to the screening and eligibility assessment of patients. Patients are invited to participate in the trial by a practice mail out. Once an expression of interest form had been received, patients attend a consultation with a clinician at their general practice. After confirming that they wish to take part in the trial patients will be contacted by a research nurse from the hospital by phone. The research nurse will randomise patients and book PAT appointments for those patients randomised to the trial arm. Patients randomised to the PAAP trial arm will be sent the “Penicillin Allergy Testing: going for a test” booklet with their hospital appointment letter. Patients randomised to the control arm will receive usual clinical care. All patients completing the PAT will receive a letter with the test result after their appointment. Patients who have tested negative will receive the “Penicillin Allergy Testing: a Negative Test Result” booklet and Intervention card by post. Practices will be informed of the test result and instructed to update the patients’ electronic health record.

For peer review only

- **9. TAILORING**: The implementation of behavioural and clinical elements of the ALABAMA intervention package will be implemented in all GP practices and local immunology clinics participating in the trial.
- **10. MODIFICATIONS**: Modifications were made to the intervention materials following qualitative feedback from the feasibility study.
- **11-12. HOW WELL**: The intervention is being tested in a feasibility trial in 8 general practices. It will then be tested in a randomised control trial across 70 GP practices.

For peer review only

BMJ Open

Developing a behavioural intervention package to identify and amend incorrect penicillin allergy records in UK general practice and subsequently change antibiotic use

Journal:	*BMJ Open*
Manuscript ID	bmjopen-2019-035793.R1
Article Type:	Original research
Date Submitted by the Author:	25-Jun-2020
Complete List of Authors:	Santillo, Marta; Oxford University, Nuffield Department of Primary Care Health Sciences<br>Wanat, Marta; Oxford University, Nuffield Department of Primary Care Health Sciences<br>Davoudianfar, Mina; University of Oxford, Clinical Trials Unit, Nuffield Department of Primary Care Health Sciences<br>Bongard, Emily; University of Oxford, Nuffield Department of Primary Care Health Sciences<br>Savic, Sinisa; University of Leeds Faculty of Medicine and Health<br>Savic, Louise; University of Leeds Faculty of Medicine and Health<br>Porter, Catherine; University of Leeds, Healthcare Associated Infection Group<br>Fielding, Joanne; University of Leeds, Healthcare Associated Infection Group<br>Butler, Christopher C.; University of Oxford,<br>Pavitt, Sue; University of Leeds<br>Sandoe, Jonathan; University of Leeds Faculty of Medicine and Health<br>Tonkin-Crine, Sarah ; University of Oxford, Nuffield Department of Primary Care Health Science
**Primary Subject Heading**:	General practice / Family practice
Secondary Subject Heading:	Infectious diseases
Keywords:	GENERAL MEDICINE (see Internal Medicine), INFECTIOUS DISEASES, Allergy < THORACIC MEDICINE

SCHOLARONE™
Manuscripts

I, the Submitting Author has the right to grant and does grant on behalf of all authors of the Work (as defined in the below author licence), an exclusive licence and/or a non-exclusive licence for contributions from authors who are: i) UK Crown employees; ii) where BMJ has agreed a CC-BY licence shall apply, and/or iii) in accordance with the terms applicable for US Federal Government officers or employees acting as part of their official duties; on a worldwide, perpetual, irrevocable, royalty-free basis to BMJ Publishing Group Ltd ("BMJ") its licensees and where the relevant Journal is co-owned by BMJ to the co-owners of the Journal, to publish the Work in this journal and any other BMJ products and to exploit all rights, as set out in our licence.

The Submitting Author accepts and understands that any supply made under these terms is made by BMJ to the Submitting Author unless you are acting as an employee on behalf of your employer or a postgraduate student of an affiliated institution which is paying any applicable article publishing charge ("APC") for Open Access articles. Where the Submitting Author wishes to make the Work available on an Open Access basis (and intends to pay the relevant APC), the terms of reuse of such Open Access shall be governed by a Creative Commons licence – details of these licences and which Creative Commons licence will apply to this Work are set out in our licence referred to above.

Other than as permitted in any relevant BMJ Author's Self Archiving Policies, I confirm this Work has not been accepted for publication elsewhere, is not being considered for publication elsewhere and does not duplicate material already published. I confirm all authors consent to publication of this Work and authorise the granting of this licence.

For peer review only

Developing a behavioural intervention package to identify and amend incorrect penicillin allergy records in UK general practice and subsequently change antibiotic use.

Corresponding author: Marta Santillo, Nuffield Department of Primary Care Health Sciences, University of Oxford, Radcliffe Observatory Quarter, Woodstock Road, Oxford, OX2 6GG, marta.santillo@phc.ox.ac.uk, 01865617935

Santillo, M. [1], Wanat, M. [1], Davoudianfar, M. [1] , Bongard, E. [1], Savic, S. [2] Savic, L. [2], Porter, C. [3], Fielding, J. [3], Butler C. C. [1], Pavitt, S. [4], Sandoe, J. [3] , Tonkin-Crine, S. [1,5] On behalf of the AlABAMA team.

[1] Nuffield Department of Primary Care Health Sciences, University of Oxford, Radcliffe Observatory Quarter, Woodstock Road, Oxford, OX2 6GG , UK

[2] Faculty of Medicine and Health, University of Leeds and Leeds Teaching Hospitals NHS Trust, Leeds LS1 3EX, UK

[3] Healthcare Associated Infection Group, University of Leeds and Leeds Teaching Hospitals NHS Trust, Leeds LS1 3EX, UK

[4] Dental Translational and Clinical Research Unit, Faculty of Medicine and Health, Worsley Building, Clarendon Way, University of Leeds, LS2 9LU, UK

[5] NIHR Health Protection Research Unit in Healthcare Associated Infections and Antimicrobial Resistance, University of Oxford, UK

SHORT RUNNING TITLE: Intervening to amend incorrect penicillin allergy records in general practice

KEYWORDS: intervention development, penicillin allergy testing, general practice

Words: 4548/4000

For peer review only

For peer review only - http://bmjopen.bmj.com/site/about/guidelines.xhtml

ABSTRACT

Words: 297/300

Objectives To develop a behavioural intervention package to support clinicians and patients to amend incorrect penicillin allergy records in general practice. The intervention aimed to: 1) support clinicians to refer patients for penicillin allergy testing (PAT), 2) support patients to attend for PAT and 3) support clinicians and patients to prescribe or consume penicillin, when indicated, following a negative PAT result.

Methods Theory-based, evidence-based and person-based approaches were utilised in the intervention development. We used evidence from a rapid review, two qualitative studies, and expert consultations with the clinical research team to identify the intervention 'guiding principles' and develop an intervention plan. Barriers and facilitators to the target behaviours were mapped to behaviour change theory in order to describe the proposed mechanisms of change. In the final stage, think-aloud interviews were conducted to optimise intervention materials.

Results The collated evidence showed that the key barriers to referral of patients by clinicians were limited experience of referral and limited knowledge of referral criteria and PAT. Barriers for patients attending PAT were lack of knowledge of the benefits of testing and lack of motivation to get tested. The key barriers to the prescription and consumption of first-line penicillin following a negative test result were patient and clinician beliefs about the accuracy of PAT and whether taking penicillin was safe. Intervention materials were designed and developed to address these barriers.

Conclusions We present a novel behavioural intervention package designed to address the multiple barriers to uptake of PAT in general practice by clinicians and patients. The intervention development details how behaviour change techniques have been incorporated to hypothesise how

For peer review only - http://bmjopen.bmj.com/site/about/guidelines.xhtml

the intervention is likely to work to help amend incorrect penicillin allergy records. The intervention will go on to be tested in a feasibility trial and randomised controlled trial in England.

ARTICLE SUMMARY

Strengths and limitations of this study

- Intervention development was conducted using a systematic and iterative process.
- Evidence-, theory and person-based approaches were used which consisted of three iterative stages: collating the evidence on views and experiences of PAT, intervention planning informed by theory and optimisation of intervention materials.
- These approaches were used to increase the feasibility and acceptability of the intervention to its target users (GPs and patients).
- This approach clearly maps how the intervention components are hypothesized to change clinician and patient target behaviours.
- The views of other general practice prescribers (such as nurse prescribers and pharmacists) should be considered in future research.

For peer review only - http://bmjopen.bmj.com/site/about/guidelines.xhtml

BACKGROUND

Incorrect penicillin allergy records are common, as side effects and symptoms of illness can be mistaken for allergic reaction symptoms [1]. About 6% of the UK general practice population has a record of a penicillin allergy but fewer than 10% of these patients are likely to be truly allergic [2]. Penicillin allergy refers to an allergy to a whole group of antibiotics (penicillins) which are first line antibiotics for many common infections. As a result of incorrect penicillin allergy labels, a significant portion of the population is denied effective antibiotics. Moreover, penicillin allergy records are also linked to antimicrobial resistance (AMR): evidence suggests that patients with a penicillin allergy label are more likely to be prescribed broad spectrum antibiotics and to acquire antibiotic resistance infections [3 4].

Penicillin allergy testing is already provided in the NHS in specialist clinics [5] and offers the opportunity to confirm or discount a penicillin allergy label. This service is only available to a subset of patients following NICE guidance [6]. Current assessment is performed over two clinic visits.

The AlABAMA (Allergy Antibiotics and Microbial resistance) study aims to develop a behavioural intervention package for UK general practice to effectively amend incorrect penicillin allergy records. The intervention package is designed to target both general practice clinicians and patients with a suspected incorrect penicillin allergy record. It introduces a pre-emptive 'penicillin allergy assessment pathway' (PAAP) which targets patients assessed as "low risk" of true allergy, who are not at risk of anaphylaxis or other severe adverse reactions, and it aims to streamline the test process by undertaking patient history screening in general practice (Stage 1) and introducing an efficient one-stop procedure at a hospital clinic for the penicillin allergy test (PAT). The test includes either a skin test (ST), testing penicillin solution on the forearm (Stage 2), and oral challenge test (OCT), taking doses of penicillin solution over time (Stage 3), or just OCT depending on the individual patient. Following the PAT, patients and practices would receive confirmation of a patient's allergy

For peer review only - http://bmjopen.bmj.com/site/about/guidelines.xhtml

status. The AlABAMA intervention will be trialled in a national randomised controlled trial in winter 2020-2021. It will test whether changes removal of incorrect penicillin allergy records and subsequent penicillin prescribing will affect treatment response for patients taking antibiotics for infections.

This paper describes the planning, development and optimisation of the AlABAMA PAAP intervention package.

METHODS AND RESULTS

Intervention planning methodology

We followed an integrated approach to intervention development that combines theory-, evidence- and person-based approaches [7-9]. This approach has been successfully used in the development of a variety of behavioural interventions including for reduction of antibiotic prescriptions in European general practice [10].

Patient and Public Involvement (PPI)

The AlABAMA programme grant involved PPI members, as co-applicants, and study advisors from the start so research questions were informed by their input. Results of all work from the AlABAMA programme will be disseminated with the help from our PPI co-apps and our existing PPI networks towards the end of the programme grant.

The AlABAMA intervention package was developed in three stages described below. The methods and key results for each stage are presented below.

For peer review only - http://bmjopen.bmj.com/site/about/guidelines.xhtml

Stage 1: Collating and analysing evidence

Rapid review methods

The full rapid review is available elsewhere [11]. Rapid review is a form of synthesis which supports the review of existing evidence in a timely manner [12]. It aimed to explore clinicians' and patients' views and experiences of penicillin allergy testing services.

Results

The review identified only two studies which reported patients' views of PAT. The first study sought patients' views on undertaking a three-step testing procedure consisting of prick testing, intradermal testing and oral challenge[13] . It found that patients thought that PAT provided a valuable medical information; however, this was measured using a single-item questionnaire ("Do you think undergoing testing for penicillin allergy provides important information for your penicillin history"). The second study recruited patients who attended general allergy clinic and were given a brief 5-question survey examining their views of PAT[14] . This study found that patients were not aware of penicillin allergy testing and had limited understanding of penicillin allergy, but also expressed their interest in being tested.

The review also identified six studies on clinicians' views of PAT[13, 15-19]three of which specifically focused on barriers and facilitators to using PAT [13, 17, 19]. One of the studies included both patient and clinician views [19]. Clinicians reported several barriers to referring patients for PAT. These included difficulties establishing the allergy history, lack of knowledge on referral processes and organisational pressures making allergy testing a low priority [13,17,19]. A number of clinicians and patients reported being reluctant to prescribe or consume penicillin after a negative PAT result.

Qualitative methods

Full details of the qualitative work are available elsewhere[20]. Two qualitative studies were undertaken by MW; one interviewing 19 general practitioners and the second interviewing 31 patients with a penicillin allergy record (16 with previous experience of PAT). The aim was to identify

For peer review only - http://bmjopen.bmj.com/site/about/guidelines.xhtml

clinicians' and patients' views on the barriers and facilitators for PAT and antibiotic use after a negative test. Semi-structured interviews were conducted over the phone by an experienced qualitative researcher (PhD qualified) with substantial previous experience of conducting qualitative research. Clinicians were identified in general practices and by the local microbiology services. Patients were identified from a general adult hospital allergy clinic and from general practices in the same geographical area. Interview topic guides were informed by existing literature which indicated potential barriers and facilitators to target behaviours.[20] An inductive thematic analysis approach was used to analyse data[21].

Results

Clinicians reported that they often felt that penicillin allergy records were incorrect however reported uncertainty about how to identify patients with true penicillin allergy and were reluctant to amend medical records without objective evidence. Penicillin allergy status was not seen to be a major problem in general practice due to the availability of alternative antibiotics and clinicians struggled to identify the risks of incorrect allergy records. Clinicians were seen to differ in their consultation styles when speaking to patients about their antibiotic prescribing decisions and allergy status. They reported lack of experience of PAT services and the need for more information on referral criteria. Regarding the process of changing a patients' record after a negative test result, clinicians reported being happy to update medical records on directions from secondary care but were unsure who was responsible for making sure that patients understood allergy test results.

The majority of patients who were motivated to get tested had experienced a negative consequence of having a penicillin allergy label (such as limited availability of antibiotics they could use). Patients reported concerns about the possibility of having an allergic reaction during PAT; the degree of the severity of their previous reactions affected how apprehensive they were about the test. Moreover, when the test was perceived as more invasive, e.g. the OCT compared to the ST, patients reported being more concerned about PAT. Patients were also concerned about how much they would be

For peer review only - http://bmjopen.bmj.com/site/about/guidelines.xhtml

monitored during the test and highlighted the importance of feeling informed, safe and observed by qualified professionals. Some patients reported being unsure about a negative test result and feeling anxious about taking penicillin if prescribed, as they doubted whether the test result was accurate.

Expert Discussions

As part of this first stage we consulted our wider clinical research team using monthly tele-conferences and emails to gain their feedback on several aspects of the intervention development. The wider clinical research team included a consultant immunologist, a consultant microbiologist, a consultant anaesthetist, a general practitioner and professors with expertise in applied health research.

Results

The expert discussions informed the interpretation of the evidence collated in the rapid review and the qualitative studies, the development of early iterations of the intervention materials, and the development of the initial intervention plan and components.

Stage 2: Intervention planning and development

Creating guiding principles

In line with the person-based approach[7] brief guiding principles were created to be consulted through the whole intervention development process. This ensured that the intervention met the original objectives. As a first step to creating guiding principles, characteristics and behavioural needs of the target users were identified based on the findings of the rapid review, qualitative

For peer review only - http://bmjopen.bmj.com/site/about/guidelines.xhtml

interviews and expert discussions. The second step involved identification of intervention objectives and key design features which would address these needs.

Results

Table 1 presents the AlABAMA guiding principles. These focused on increasing confidence to refer and attend for PAT and increasing motivation to prescribe/consume penicillin following a negative PAT result. Guiding principles also included increasing clinician confidence in discussing penicillin allergy with patients and improving communication between primary and secondary care about penicillin allergy status. Lastly the intervention needed to present the PAAP as reliable and trusted and provide accessible and easy to use materials for clinicians and patients.

Table 1: Guiding Principles for the AlABAMA intervention package.

PAAP =Penicillin Allergy Assessment Pathway; PAT = Penicillin Allergy Testing

Intervention design objectives	Key features
To present the PAAP as a reliable and trusted approach to confirm allergy status	• Present the PAAP as a trusted/scientific/official way to get a confirmed PAT result, for both clinicians and patients
To increase clinician's confidence in referring patients, and patients' motivation to attend, for PAT	• Provide evidence on the potential harms of an incorrect penicillin allergy records and the process of testing in the Information Pack<br>• Provide opportunity to address patients' concerns about potential benefits and risks of testing by both clinicians and allergists during consultations before the allergy test and during the appointment at the immunology clinic

Intervention design objectives	Key features
To motivate clinicians/patients to prescribe/take penicillin following a negative PAT test result	• Provide information to clinicians about the clinical meaning of a test result and its implication as part of the Information Pack<br>• Provide information to patients about the PAT (process) and the potential benefits of being able to take penicillin in the Pre-test Intervention Booklet
To increase clinicians' ability to discuss PAT with patients	• Provide training for general practice clinicians about penicillin allergy and PAT, including its accuracy, implications, benefits
To improve communication between primary and secondary care so that allergy status records are correct	• Provide a clear and consistent approach to de-labelling with support from colleagues in secondary care and preventing re-labelling in the future<br>• Provide information about who is responsible for ensuring patients understand the results and for updating the medical records during the site training and in the Information Pack<br>• Provide clear and precise documentation of side effects during future courses of penicillin in the Information Pack
To provide easily accessible tools that are suitable for use by general practice clinicians	• Make interventions materials for clinicians accessible, short, easy to follow, easy to implement and not increase workload
To provide easily accessible resources for patients	• Make patients materials brief, easy to read, accessible<br>• Make materials easy to carry with them at all time and that provide evidence of the results of their test

194

195 **Behavioural analysis**

196 The aim of the behavioural analysis was to use behaviour change theory to describe the content of
197 the AlABAMA intervention package and map the evidence from the rapid review, qualitative studies

For peer review only - http://bmjopen.bmj.com/site/about/guidelines.xhtml

198 and expert consultations. We used a model of behaviour known as COM-B (Capability, Opportunity, Motivation, Behaviour)[22]. According to this model, behaviour is a result of these three constructs.. Changing behaviour will require a change in these three constructs. The Behaviour Change Wheel (BCW)[23] offers a systematic way to identify types of intervention component which are best suited to changing each of these components. A more specific description of elements which can be delivered within intervention components is provided in a taxonomy of 93 behaviour change techniques (Behaviour Change Technique Taxonomy, v1; BCTT v1)[24]."

Methods

The first step of the behavioural analysis process was to define the problem in behavioural terms which resulted in the identification of the fours specific target behaviours.

As a second step, we identified what needed to change using the Theoretical Domain Framework (TDF)[25] which includes 14 domains such as knowledge, skills, beliefs about capabilities. Each domain of the TDF relates to a COM-B component. This step allowed us to identify target behaviours, their barriers and facilitators, and how intervention components would support desired behaviour change based on evidence collated in stage 1.

Finally, the intervention components were mapped to the TDF framework and the BCW referring to the BCTv1[24]. This produced a list of TDF barriers to the target behaviours, target constructs (what needs to change for the behaviour to occur), intervention functions (ways an intervention can change behaviour) and behaviour change techniques used for each of the barriers/facilitators.

Results

The full behavioural analysis is presented in supplementary materials (S1). Firstly, we identified barriers and facilitators to referral of low risk patients to PAT and patient attendance to PAT. The analysis highlighted that both clinicians' and patients' knowledge and perceptions of penicillin

For peer review only - http://bmjopen.bmj.com/site/about/guidelines.xhtml
For peer review only

allergy and test procedures could be modified; information needed to be supported by scientific evidence for clinicians and patients to be reassured that the test was safe. We designed a resource for clinicians entitled "Penicillin Allergy Testing: Information for general practice" which contained information on penicillin allergy and PAAP procedures. As part of the AlABAMA trial, this will be supported by site training and working instructions which provide practical guidance on screening patients and referral to PAT (relevant BCTs for clinicians were 'information about antecedents' and 'information about health consequences').

For patients we developed two patient booklets, one to be provided prior to PAT and one following a negative test result. All patients, on entering the trial, will have a consultation with a GP to answer questions and address concerns about PAT. We developed a patient booklet entitled "Penicillin Allergy Testing: going for a test" which included information on PAAP procedures and PAT safety (relevant BCTs for patients were 'information about health consequences' and 'feedback on outcomes of the behaviour').

The barriers to the prescription and consumption of first-line penicillin following a negative test result were patient and clinician beliefs about the accuracy of PAT and whether taking penicillin was safe. Clinicians also needed reassurance that colleagues saw de-labelling as beneficial and resources to support them in changing incorrect penicillin allergy records. We developed a second patient booklet entitled "Penicillin Allergy Testing: a negative test result", which contained information about which antibiotics patients could safely take in the future following a negative test result, a negative result intervention card and a result letter which confirmed the patient allergy status to penicillin (relevant BCTs were 'social support' and 'restructuring of the social and physical environment'). As part of the trial, clinicians will receive working instructions, which contain guidance on how to change the patient allergy label in medical records, result letter which confirms the patient allergy status to penicillin, and an electronic-pop up, which includes a reminder of the

For peer review only - http://bmjopen.bmj.com/site/about/guidelines.xhtml

patient's new allergy status (relevant BCTs were 'feedback on outcomes of behaviour' and 'adding objects to the environment').

Logic modelling

The next step included the development of a logic model, which summarised the behavioural analysis, providing a diagrammatic representation of the hypothesised processes and causal pathways from the intervention components to the desired outcomes.[26-27]

Methods

The research team opted for a process oriented iterative logic model which was refined during the whole intervention development stage.

Results

The logic model (Figure 1) included four components:

Intervention components and techniques: Intervention components were organised based on the two target groups (clinicians and patients). Intervention techniques summarised the BCTs used as part of the intervention which were identified in the behavioural analysis.

Intervention processes: These were the psychological factors which explained the relationships between the intervention components and the outcome of the intervention. These processes were described in terms of TDF domains. Each intervention technique was hypothesised to mainly affect one of these processes. As part of the intervention, clinicians would receive information on penicillin allergy and implications of incorrect allergy records in order to increase their knowledge of PAT and allergies. In addition to this, providing medical and scientific evidence and current guidelines on how to perform PAT would change their professional identity related to their role in the referral process and act as reinforcement to increase their motivation to refer patients in the future. Regarding the processes affecting patient's attendance of PAT, providing evidence of penicillin as best treatment

For peer review only

and the safety of PAT would increase their beliefs of positive consequences of accurate records; informing them of common reactions during PAT and reassuring them of monitoring at the clinic and at home would increase their knowledge on PAT, decrease their negative emotions, such as anxiety, and act as positive reinforcement to attend the clinic.

Both patients and clinicians would receive evidence of the accuracy of PAT, and of safety of prescribing penicillin after a negative test result, in order to increase their belief about positive consequences of prescribing penicillin after a negative test result. Finally, providing a negative test result card to use with clinicians would affect patients' expectations of social influences regarding clinician's acceptance of the test result.

Purported mediators: These are the target behaviours of the intervention which directly affect the outcomes. In the logic model the assessment of potential incorrect penicillin records was operationalised as the referral of low risk patients to PAT and patient attendance at PAT. The introduction of the PAAP was hypothesized to affect the change of incorrect penicillin allergy records (clinician changing medical records and patient acceptance of change of penicillin allergy status) which would ultimately affect the consumption of penicillin.

Outcomes: The behavioural outcomes of the model were the prescription and consumption of first-line penicillin when indicated.

Stage 3: Optimising the intervention materials

The development of the intervention materials was part of an iterative process which was initially informed by the findings of the rapid review, qualitative interviews and expert consultations. Once the first drafts of the intervention materials were developed, a series of think-aloud interviews with clinicians and patients were conducted in order to gain their insights on the content, usability and format of the clinician "Penicillin Allergy Testing: Information for general practice" leaflet and two

For peer review only - http://bmjopen.bmj.com/site/about/guidelines.xhtml

patient booklets ("Penicillin Allergy Testing: going for a test", "Penicillin Allergy testing: a negative test result"). Subsequently to the feedback received by the participants in the think-aloud interviews and discussions in further expert consultations, the intervention materials were optimised. Think-aloud interviews were carried out as part of intervention development and not as a piece of qualitative research, as such ethical approval was not required. Interviews were not recorded and interviews were only used to refine intervention materials.

For peer review only

Think-aloud interviews with GPs

Methods

Think aloud telephone interviews were conducted with 6 participants by MW; 2 additional participants provided feedback via email. Four of the participants had previously taken part in the qualitative study on GP views on the barriers and facilitators for PAT and antibiotic use after a negative test (mentioned in the section Qualitative methods above Ref); four additional participants were recruited using convenience sampling. Interviews were not recorded, but the interviewer took detailed notes. Interviews focused on GP views of each section of the "Penicillin Allergy Testing: Information for general practice" (supplementary materials S2).

Results

The leaflet was well received. Participants reported that it was informative, useful and generally easy to read. The participants perceived it not only as information for themselves, but also a tool to use in a consultation with patients. Some participants felt that they knew about the consequences of incorrect penicillin allergy record; and therefore the leaflet could be shortened. Most participants understood the testing stages; however, a couple of participants were confused about which stages of the test patients could skip. One participant wanted exact doses of penicillin specific (rather than just amounts). Regarding the section on patient discussions, some clinicians felt that there was no

For peer review only - http://bmjopen.bmj.com/site/about/guidelines.xhtml

need to discuss the test with patients. Participants queried whether being tested with amoxicillin meant that the patient could now take all penicillin based antibiotics and wanted more information.

Clinicians' feedback was collated and organised in a 'table of changes' (supplementary materials S3) where suggested changes were listed and given a level of priority for that change, following the MoScoW framework [28], and the source of the suggested change (expert opinion, research team, clinical research team, literature review). Changes to the "Penicillin Allergy Testing: Information for general practice" leaflet included changes to the title, to the exact doses of penicillin given to the patients during the test, information about side effects and information about which antibiotics patients with a negative test result can take safely.

Think-aloud interviews with patients

Methods

Think aloud interviews were conducted with 7 patients (3 with experience of PAT and 4 with no experience) by MW. Five of these patients had previously taken part in the qualitative study on views on the barriers and facilitators for PAT and antibiotic use after a negative test (mentioned in the section Qualitative methods above Ref). These participants were asked at the end of the initial interview whether they would like to comment on the intervention materials at a later date; the additional two participants were identified using convenience sampling. Again, interviews were not recorded, but the interviewer took detailed notes. Interviews asked their views about the two patient booklets ("Penicillin Allergy Testing: going for a test", "Penicillin Allergy testing: a negative test result") and the intervention card (supplementary materials S2).

Results

The booklets and intervention card were very well received by the participants. Participants considered the booklets to have the right amount of information and felt they were generally easy to read. Patients reported that the booklets convinced them that going for a PAT could be beneficial.

For peer review only - http://bmjopen.bmj.com/site/about/guidelines.xhtml

For peer review only

They felt that they could relate to the description of how people were given penicillin allergy labels. Patients thought the description of the test was clear and they knew what to expect. Statistics about the prevalence of allergy were not always understood by the participants, as the participants often thought that 1 in 10 people are allergic and they wanted a more visual presentation of this key information. Participants were unsure what narrow and broad spectrum antibiotics were and did not recognise MRSA abbreviation. Participants did not always know that penicillin is more than one antibiotic. The participants wanted to have a separate paragraph on what could happen during the test and what could happen during three days of taking penicillin at home. They also wanted reassurance that three days would be enough to detect delayed reactions. The participants wanted more reassurance that after being tested with one type of penicillin (e.g. amoxicillin), it would mean that they could safely take all penicillin antibiotics. The participants were slightly concerned about the risk of allergic reaction in the future (despite negative test results).

Patient feedback was collated in a table of changes. Changes made to the booklets were the selection of new images of patients for the front cover, inclusion of definitions of narrow and broad spectrum antibiotics, and reassurance that 3 days of oral challenge would be enough to detect delayed reactions to penicillin.

Intervention components

The development and optimisation of clinician and patient intervention materials was part of an iterative process . Figures 2 and 3 present the example of this process for the development of one section of the “Penicillin allergy testing: going for a test” patient booklet and the “Information for General Practice” leaflet for clinicians.

All Working Instructions developed to support clinicians and research nurses activities as part of the AlABAMA intervention package were shown to a group of clinicians to gain their feedback on content and layout. Among the clinicians who provided feedback there were two practice managers,

For peer review only - http://bmjopen.bmj.com/site/about/guidelines.xhtml

and one nurse. Their overall feedback was positive and the main changes to the intervention materials included the identification of the best way of updating the patient's medical records after PAT, and the introduction of screenshots of the medical record in the working instructions.

All participants letter (patient appointment letter, patient result letter, clinician result letter) were developed among the wider clinical and research team in order to make them effective in motivating patient to attend the penicillin allergy testing and in order to persuade clinician to change patient records and prescribe penicillin after a negative test result, and patient to consume penicillin after a negative test result.

At the end of the intervention development stage, a description of the intervention was completed following the TIDieR [29] guidance (supplementary materials S4) together with a description of the intervention components for clinicians and patients (Table 2). We have followed the GUIDED[30] checklist (supplementary materials S5), when reporting intervention development in this paper.

Table 2: Overview of the key intervention components of the AlABAMA intervention package.

PAT = Penicillin Allergy Testing

Intervention components	Description
**For clinicians**	
"Penicillin Allergy Testing: Information for general practice" leaflet	Information leaflet which includes evidence-based information to increase knowledge about penicillin allergy testing and motivation to refer patients to PAT and prescribe penicillin after a negative PAT result
Electronic health record pop-up	Electronic pop-up on medical records which tells clinicians that patient has had a negative test result and that their allergy status

For peer review only

	needs to be amended
Allergy result letter	A letter is sent to the GP to inform them of the patient's penicillin allergy test result, including details on which test(s) they undertook, whether it is safe or not to prescribe penicillin in the future and instructions on how to change the allergy label
Consultation with patients	Discussions with patients to check eligibility for the trial and to answer any queries about the trial and PAAP testing
Site Training and working instructions	Training in trial procedures delivered to GP leads including provision of information on penicillin allergy de-labelling, the referral process, the three stages of the PAAP and the interpretation of test results
**For patients in intervention arm**	
"Penicillin allergy testing: going for a test" booklet	A booklet to inform patients, in the PAAP intervention arm, about incorrect allergy records, how they may benefit from having a penicillin allergy test and what the test involves
Penicillin Allergy Test appointment letter	The letter includes information on the PAAP procedures in hospital, including pre-test assessment and monitoring during the test and at home
Penicillin Allergy Assessment Pathway (PAAP)	Appointment at the immunology clinic for patients in the PAAP intervention arm. At the appointment patients will complete Stage 1, Stage 2 and/or Stage 3 of the PAAP pathway
Allergy test result letter	Patient will receive a letter with information on the result of their allergy test and whether it is safe or not to take penicillin in the future
"Penicillin allergy testing: a negative test result" booklet	A booklet on the reliability of the test results and consequences of a negative test result (sent with allergy test result letter).
Post-test intervention card	Laminated credit card-sized card which says which test the patient has had and confirms the negative allergy result
For all patients in control and intervention arm	
An invitation letter and Participant	An invitation letter and Participant information Sheet on the purpose

For peer review only - http://bmjopen.bmj.com/site/about/guidelines.xhtml

information Sheet	of the trial and what the research study would involve for patient participants
Discussion with clinicians	Discussion with clinicians about attending the test to ask any queries around the benefits of taking the test and why removing the incorrect record might be good

DISCUSSION

This paper describes the development of the AlABAMA intervention package, which aimed to change behaviours that facilitate penicillin allergy testing and subsequently amend incorrect allergy records in English general practice. Such changes in clinician and patient behaviours have potential to significantly impact on antibiotic prescribing and consumption. The approach used here has previously been used for the development of behaviour change interventions targeted to reduce antibiotic prescribing by clinicians in primary[31] and secondary care[32] but it is the first time that this approach has been used to develop an intervention to amend incorrect penicillin allergy records. The transparency of the intervention development process will inform intervention developers on how this methodology could be used in different contexts, and will facilitate the comparison with other interventions which have used similar processes.

The evidence collated from the rapid review and qualitative interviews allowed in-depth understanding of participant needs (lack of knowledge of PAT, lack of knowledge of negative consequences of allergy labels) and which behavioural influences needed to be modified as part of the intervention. The mapping of these behavioural influences and the AlABAMA intervention package components to behaviour change theory and the logic model allowed a transparent reporting of the psychological processes that are hypothesized to explain the effect of the intervention components on the outcomes. More specifically it highlighted the BCTs used in each component of the AlABAMA intervention package and how they addressed the barriers identified in

For peer review only

the rapid review and qualitative work. It also explained which psychological mechanisms were hypothesised to change by the intervention components (clinicians' knowledge on penicillin allergy and PAT procedures, patients' beliefs about positive consequences of taking the PAT) and which target behaviours (referral and attendance to PAT, prescription and consumption of penicillin first line) would likely affect the intervention outcomes. Think-aloud interviews with patients and clinicians, and in-depth feedback from the clinical research team on the intervention materials, highlighted changes that needed to be made in order to increase their acceptability and potential effectiveness.

The AlABAMA intervention package targeted clinician referral of patients for PAT and updating incorrect penicillin allergy records; factors previously identified in previous qualitative research as barriers to effective penicillin allergy de-labelling.[11-20] Recent exploration of clinician reported barriers and enablers to identifying and de-labelling hospital inpatients with incorrect penicillin allergy records has highlighted the need to introduce patient education concerning the risks of avoiding penicillin.[24] Inconsistencies in the management of penicillin allergic patients were reported, together with a lack of time to discuss allergy testing, and the need to improve communication between primary and secondary care about patient allergy status, as well as updating of patient medical records.[33] A previous exploration of views about implementing de-labelling of patients ahead of elective surgery identified barriers to implementing it on a large scale, such as human factors linked to anxiety and financial implications. These human factors were: lack of interest from patients in undertaking an allergy test; lack of acceptance of the test result among clinicians; high proportion of patient re-labelled themselves after a negative testing for penicillin allergy or re-labelling by health care professionals. The financial barrier was significant despite long-term cost benefit, as there is an upfront cost to perform the test.[34]

A limitation of the AlABAMA intervention package is to how widely applicable it might be. Allergy services across the UK vary significantly, and access to specialist testing ranges widely. The AlABAMA

For peer review only - http://bmjopen.bmj.com/site/about/guidelines.xhtml

programme will focus on one geographical area (the North of England), which will be covered by the specialist allergy unit participating in the AlABAMA trial. The intervention will be centred around functionality to be incorporated into SystemOne (an electronic medical record system widely used in general practice) but the intervention package is not necessarily suitable for use in other areas of England, the UK or wider. The contextual factors to delivery should be considered. Moreover, it is only a small group of patients (around 25-30%) who are suitable to undergo the abbreviated test (patient history, skin test and oral challenge test). Many will still require full assessment by an immunologist or allergist as per current guidelines. Cost-effectiveness analysis of the PAT and intervention package as a whole will be carried out the in upcoming AlABAMA trial.

Conclusions

Current clinical practice involves referral to penicillin allergy testing with little attention to other elements of the pathway that help to ensure that testing impacts positively on patient care. This study presents the development of a behavioural intervention package to support the process of amending incorrect penicillin allergy records. Numerous barriers to the uptake of PAT have been identified as well as penicillin prescribing and consumption following a negative test. We have identified relevant behaviour change techniques to inform the development of the AlABAMA intervention package to overcome these barriers. The intervention is currently being tested in a feasibility trial in primary care to lead on to a randomised-controlled trial.

For peer review only

Acknowledgements: The AlABAMA team consists of: Jenny Boards, Emily Bongard, Christopher C Butler, Mina Davoudianfar, Mandy East, Joanne Fielding, Philip Howard, Sue H Pavitt, Catherine E Porter, Jonathan AT Sandoe, Marta Santillo, Louise Savic, Sinisa Savic, Bethany Shinkins, Sarah Tonkin-Crine, Marta Wanat, Robert West, Ly-Mee Yu.

Author contributions: JS, SP, CB, SS, LS, EB and STC designed and developed the AlABAMA programme and the plan for the intervention. MW and STC led the intervention development, with

For peer review only - http://bmjopen.bmj.com/site/about/guidelines.xhtml

input from other co-authors. MW conducted the rapid review and qualitative work. MS led the behavioural analysis. CB, EB, MD, CP and JF led the design of the trial and trial materials. MS drafted the manuscript. All authors critically reviewed the manuscript and approved the final version.

Funding: This study summarises independent research funded by the National Institute for Health Research (NIHR) under its Programme Grants for Applied Research Programme (Grant Reference Number RP-PG-1214-20007). STC received additional funding from the National Institute for Health Research Health Protection Research Unit (NIHR HPRU) in Healthcare Associated Infections and Antimicrobial Resistance at the University of Oxford in partnership with Public Health England (PHE) [HPRU-2012-10041]. The research is supported by the National Institute for Health Research (NIHR) infrastructure at Leeds. The funder had no role in the design of the study; in the collection, analyses, or interpretation of data; in the writing of the manuscript, or in the decision to publish.

Disclaimer: The views expressed are those from the authors and not necessary those of the NHS, NIHR or the Department of Health and Social Care.

Competing Interests: The authors have received funding from the National Institute for Health Research.

Patient Consent: Not required

Ethics approval: This study received ethical approval from the London Bridge Research Ethics Committee (Ref: 19/LO/0176).

Data sharing statements: No additional data available

For peer review only

REFERENCES

1. McLean-Tooke A, Aldridge C, Stroud C, et al. Practical management of antibiotic allergy in adults. *Journal of Clinical Pathology* 2011;64(3):192-99. doi: 10.1136/jcp.2010.077289

2. Borch JE, Andersen KE, Bindslev-Jensen C. The prevalence of suspected and challenge-verified penicillin allergy in a university hospital population. *Basic Clin Pharmacol Toxicol* 2006;98(4):357-62. doi: 10.1111/j.1742-7843.2006.pto_230.x

3. Reddy V, Baman NS, Whitener C, et al. Drug Resistant Infections with Methicillin-Resistant Staphylococcus Aureus, Clostridium Difficile, and Vancomycin Resistant Enterococcus Are Associated with a Higher Prevalence of Penicillin Allergy. *J Allergy Clin Immunol* 2013;131(2):AB170-AB70. doi: 10.1016/j.jaci.2012.12.1269

4. West RM, Smith CJ, Pavitt SH, et al. "Warning: allergic to penicillin': association between penicillin allergy status in 2.3 million NHS general practice electronic health records, antibiotic prescribing and health outcomes. *J Antimicrob Chemother* 2019;74(7):2075-82. doi: 10.1093/jac/dkz127

5. Krishna MT, Huissoon AP, Li M, et al. Enhancing antibiotic stewardship by tackling "spurious" penicillin allergy. *Clin Exp Allergy* 2017;47(11):1362-73. doi: 10.1111/cea.13044

6. NICE. Drug allergy: giagnosis and management of drug allergy in adults, children and young poeple. NICE clinical guideline 183. 2014

For peer review only

For peer review only - http://bmjopen.bmj.com/site/about/guidelines.xhtml

7. Yardley L, Morrison L, Bradbury K, et al. The Person-Based Approach to Intervention Development: Application to Digital Health-Related Behavior Change Interventions. *Journal of Medical Internet Research* 2015;17(1) doi: 10.2196/jmir.4055

8. Greenwell K, Sivyer K, Vedhara K, et al. Intervention planning for the REDUCE maintenance intervention: a digital intervention to reduce reulceration risk among patients with a history of diabetic foot ulcers. *BMJ Open* 2018;8(5):12. doi: 10.1136/bmjopen-2017-019865

9. Band R, Bradbury K, Morton K, et al. Intervention planning for a digital intervention for self-management of hypertension: a theory-, evidence- and person- based approach. *Implementation Science* 2017;12 doi: 10.1186/s13012-017-0553-4

10. Little P, Stuart B, Francis N, et al. Effects of internet-based training on antibiotic prescribing rates for acute respiratory-tract infections: a multinational, cluster, randomised, factorial, controlled trial. *The Lancet* 2013;382(9899):1175-82.

11. Wanat M, Anthierens S, Butler CC, et al. Patient and Prescriber Views of Penicillin Allergy Testing and Subsequent Antibiotic Use: A Rapid Review. *Antibiotics-Basel* 2018;7(3) doi: 10.3390/antibiotics7030071

12. Khangura S, Polisena J, Clifford TJ, et al. RAPID REVIEW: AN EMERGING APPROACH TO EVIDENCE SYNTHESIS IN HEALTH TECHNOLOGY ASSESSMENT. *International Journal of Technology Assessment in Health Care* 2014;30(1):20-27. doi: 10.1017/s0266462313000664

13. Sundquist, B.K.; Bowen, B.J.; Otabor, U.; Celestin, J.; Sorum, P.C. Proactive penicillin allergy testing in primary care patients labeled as allergic: Outcomes and barriers. *Postgrad. Med.* **2017**

For peer review only

For peer review only - http://bmjopen.bmj.com/site/about/guidelines.xhtml

14. Jose J, Ishmael FTJJoA, Immunology C. A drug allergy education handout is an easy and effective method to improve patient awareness of penicillin allergy and increase penicillin testing. 2017;139(2):AB29.

15. Elkhalifa, S, Calisti, G, Owens, L, Garcez, T, Dodgson, K, Alexander, K. Management of patients with a history of penicillin allergy: Barriers to best practice and strategies to overcome them. *Allergy* 2017, *72*, 224.

16. Hayoun, MB, Bourrier, T, Pognonec, C, Sanfiorenzo, C, Marquette, C, Leroy, S. The impact of allergy to beta-lactam antibiotocs on general practitioners and patients in a cohort of154 French patients. *Rev. Fr. Allergol.* 2015, *55*, 333–340.

17. Picard, M, Bégin, P, Bouchard, H, Cloutier, J, Lacombe-Barrios, J, Paradis, J, Des Roches, A, Laufer, B, Paradis, L. Treatment of patients with a history of penicillin allergy in a large tertiary-care academic hospital. *J. Allergy Clin. Immunol. Pract.* 2013, *1*, 252–257.

18. Soni, D, Ramsey, A, Staicu, M. A clinical perspective: The prescriber's true understanding of the 'penicillin allergic'patient. *Ann. Allergy Asthma Immunol.* 2016, 117, S18

19. Trubiano, JA, Beekmann, SE, Worth, LJ, Polgreen, PM, Thursky, KA, Slavin, MA, Grayson, M.L, Phillips, E.J. Improving Antimicrobial Stewardship by Antibiotic Allergy Delabeling: Evaluation of Knowledge, Attitude, and Practices Throughout the Emerging Infections Network. *Open Forum Infect. Dis.* 2016, *3*

20. Wanat M, Anthierens S, Butler CC, et al. Patient and Primary Care Physician Perceptions of Penicillin Allergy Testing and Subsequent Use of Penicillin-Containing Antibiotics: A Qualitative Study. *Journal of Allergy and Clinical Immunology-in Practice* 2019;7(6):1888-+. doi: 10.1016/j.jaip.2019.02.036

For peer review only

For peer review only - http://bmjopen.bmj.com/site/about/guidelines.xhtml

21. Braun V, Clarke V. What can "thematic analysis" offer health and wellbeing researchers?. *International journal of qualitative studies on health and well-being.* 2014;9.

22. Michie S, Atkins L, West R. The behaviour change wheel: a guide to designing interventions. Needed: physician leaders. 2014;26:146.

23. Michie S, van Stralen MM, West R. The behaviour change wheel: A new method for characterising and designing behaviour change interventions. *Implementation Science* 2011;6 doi: 10.1186/1748-5908-6-42

24. Michie S, Richardson M, Johnston M, et al. The Behavior Change Technique Taxonomy (v1) of 93 Hierarchically Clustered Techniques: Building an International Consensus for the Reporting of Behavior Change Interventions. *Annals of Behavioral Medicine* 2013;46(1):81-95. doi: 10.1007/s12160-013-9486-6

25. Atkins L, Francis J, Islam R, et al. A guide to using the Theoretical Domains Framework of behaviour change to investigate implementation problems. *Implementation Science* 2017;12 doi: 10.1186/s13012-017-0605-9

26. Baxter S, Killoran A, Kelly MP, et al. Synthesizing diverse evidence: the use of primary qualitative data analysis methods and logic models in public health reviews. *Public Health* 2010;124(2):99-106. doi: 10.1016/j.puhe.2010.01.002

27. Baxter SK, Blank L, Woods HB, et al. Using logic model methods in systematic review synthesis: describing complex pathways in referral management interventions. *Bmc Medical Research Methodology* 2014;14 doi: 10.1186/1471-2288-14-62

28. Bradbury K, Morton K, Band R, et al. Using the Person-Based Approach to optimise a digital intervention for the management of hypertension. *PloS one* 2018;13(5):e0196868.

For peer review only - http://bmjopen.bmj.com/site/about/guidelines.xhtml

29. Hoffmann TC, Glasziou PP, Boutron I, et al. Better reporting of interventions: template for intervention description and replication (TIDieR) checklist and guide. *Bmj-British Medical Journal* 2014;348 doi: 10.1136/bmj.g1687

30. Duncan E, O'Cathain A, Rousseau N, Croot L, Sworn K, Turner KM, Yardley L, Hoddinott P. Guidance for reporting intervention development studies in health research (GUIDED): an evidence-based consensus study. BMJ open. 2020 Apr 1;10(4):e033516.

31. Anthierens S, Tonkin-Crine S, Douglas E, et al. General practitioners' views on the acceptability and applicability of a web-based intervention to reduce antibiotic prescribing for acute cough in multiple European countries: a qualitative study prior to a randomised trial. *BMC family practice* 2012;13(1):101.

32. Santillo M, Sivyer K, Krusche A, et al. Intervention planning for Antibiotic Review Kit (ARK): a digital and behavioural intervention to safely review and reduce antibiotic prescriptions in acute and general medicine. *J Antimicrob Chemother* 2019

33. Powell N, Wilcock M, Roberts N, et al. Focus group study exploring the issues and the solutions to incorrect penicillin allergy-labelled patients: an antibiotic stewardship patient safety initiative. *European Journal of Hospital Pharmacy* 2019:ejhpharm-2019-001863.

34. Savic L, Gurr L, Kaura V, et al. Penicillin allergy de-labelling ahead of elective surgery: feasibility and barriers. *Br J Anaesth* 2019;123(1):E110-E16. doi: 10.1016/j.bja.2018.09.009

For peer review only - http://bmjopen.bmj.com/site/about/guidelines.xhtml

Figure 1: AlABAMA logic model

Figure 2: Example of intervention development for clinician materials

Figure 3: Example of intervention development for patient materials. PAT = Penicillin Allergy Testing

For peer review only

For peer review only - http://bmjopen.bmj.com/site/about/guidelines.xhtml

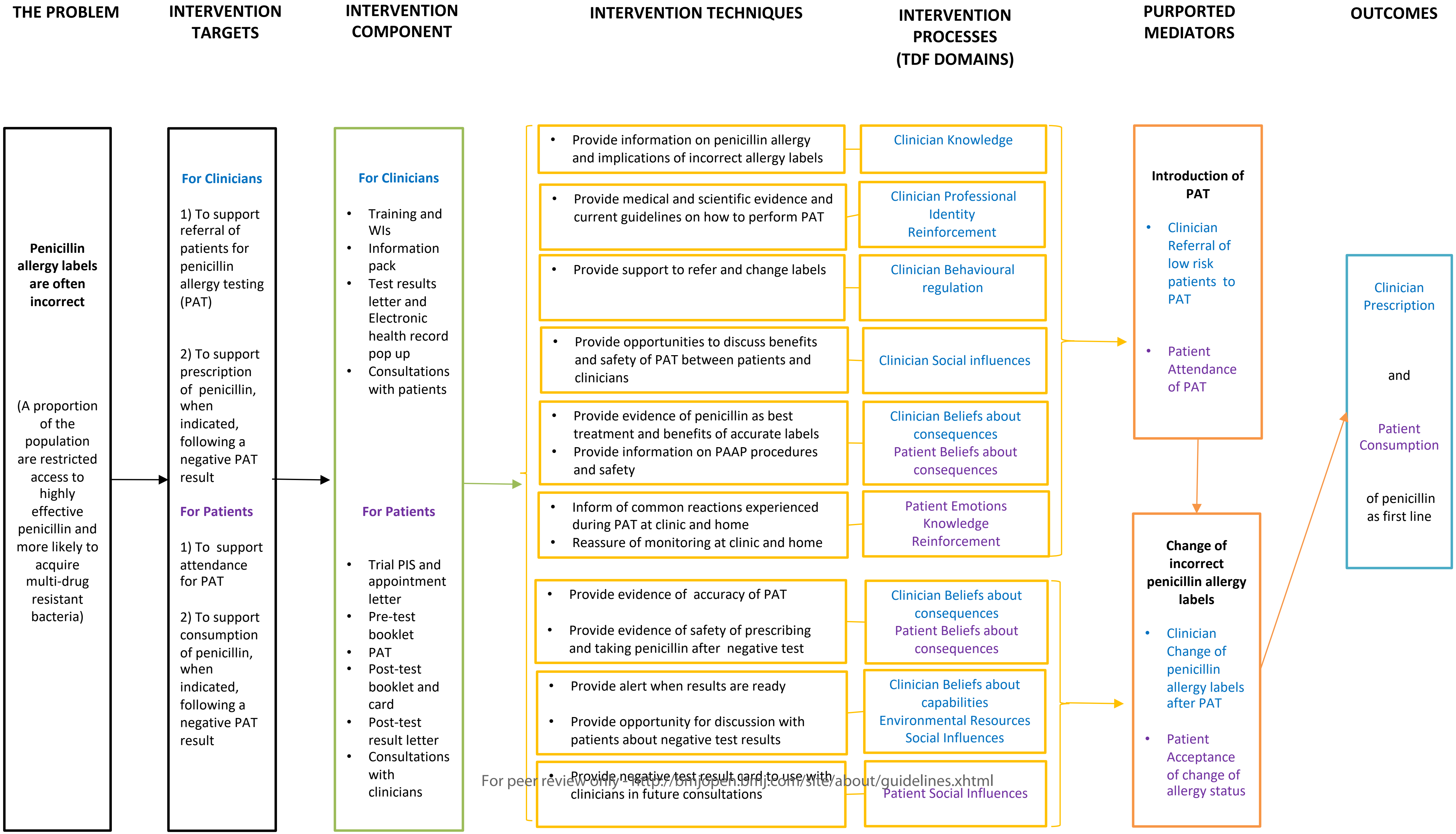

For peer review only - http://bmjopen.bmj.com/site/about/guidelines.xhtml

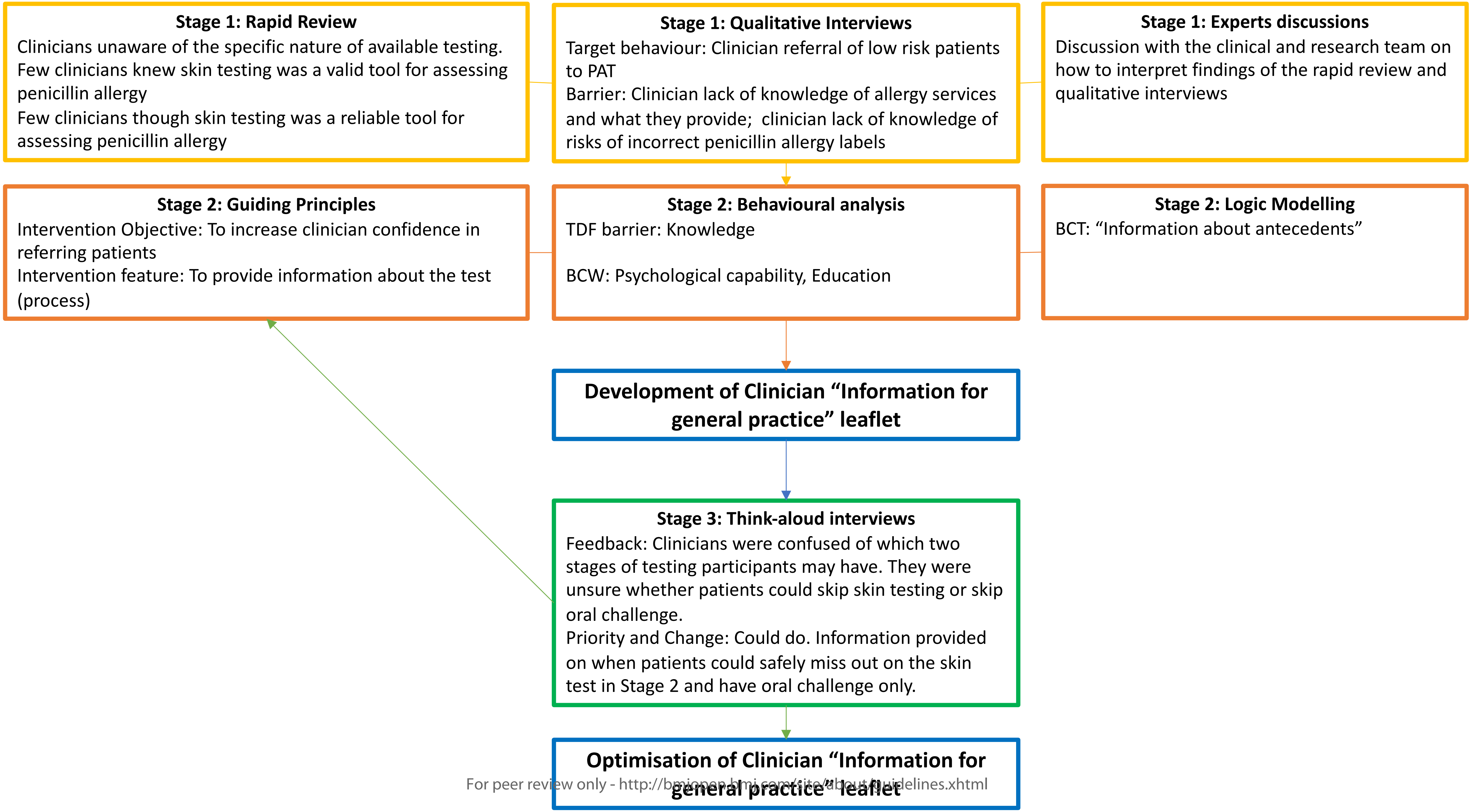
Stage 1: Rapid Review
Clinicians unaware of the specific nature of available testing.
Few clinicians knew skin testing was a valid tool for assessing penicillin allergy
Few clinicians though skin testing was a reliable tool for assessing penicillin allergy
Stage 1: Qualitative Interviews
Target behaviour: Clinician referral of low risk patients to PAT
Barrier: Clinician lack of knowledge of allergy services and what they provide; clinician lack of knowledge of risks of incorrect penicillin allergy labels
Stage 1: Experts discussions
Discussion with the clinical and research team on how to interpret findings of the rapid review and qualitative interviews
Stage 2: Guiding Principles
Intervention Objective: To increase clinician confidence in referring patients
Intervention feature: To provide information about the test (process)
Stage 2: Behavioural analysis
TDF barrier: Knowledge
BCW: Psychological capability, Education
Stage 2: Logic Modelling
BCT: “Information about antecedents”
Development of Clinician “Information for general practice” leaflet
Stage 3: Think-aloud interviews
Feedback: Clinicians were confused of which two stages of testing participants may have. They were unsure whether patients could skip skin testing or skip oral challenge.
Priority and Change: Could do. Information provided on when patients could safely miss out on the skin test in Stage 2 and have oral challenge only.
Optimisation of Clinician “Information for general practice” leaflet

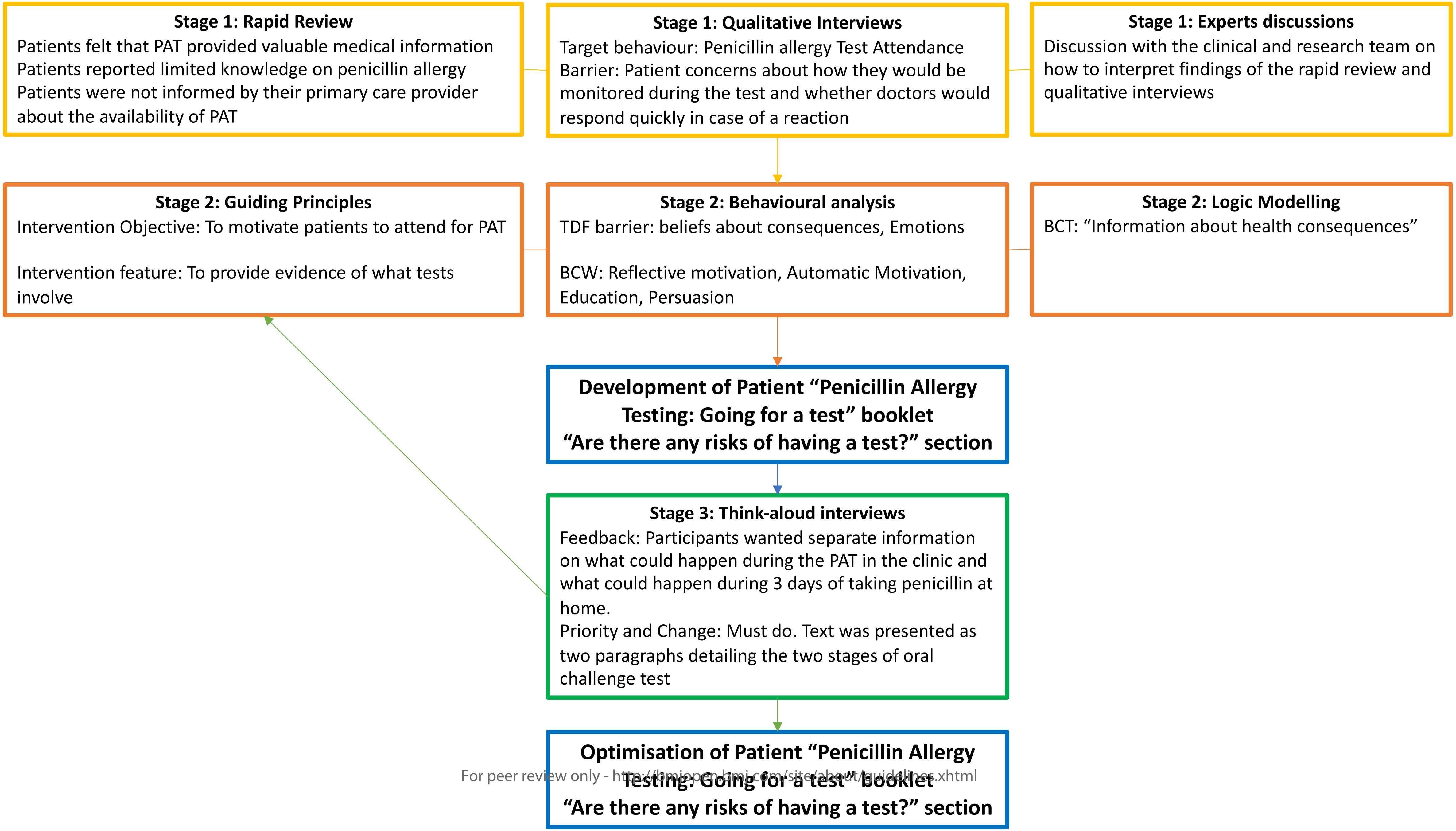

For peer review only - http://bmjopen.bmj.com/site/about/guidelines.xhtml

SUPPLEMETARY MATERIALS

S1. Behavioural Analysis Table AlABAMA

S2. Think-aloud topic guides for clinicians and patients

S3. Table of changes: "Penicillin Allergy Testing: going for a test" booklet

S4. Full description of the AlABAMA (ALlergy AntiBiotics And Microbial resistAnce) Intervention package in accordance with the Template for Intervention Description and Replication (TIDieR) framework

S5. GUIDED – a guideline for reporting for intervention development studies.

S1. Behavioural Analysis Table

Target Behaviours:

- Clinician referral of low risk patients to undertake the Penicillin Allergy Testing (PAT)
- Clinician prescription of penicillin after negative test result
- Patient attendance at Penicillin Allergy Testing (PAT)
- Patient consumption of penicillin when prescribed after negative test result

Notes: [QI]: Qualitative Interviews; [RR]: Rapid Review; [LR]: Literature Review; PIS; Participant Information Sheet

Table S1a Behavioural Analysis Table for the AlABAMA intervention, clinician behaviours

For peer review only

**Barriers / *facilitators* to target behaviours**	**Intervention Component/s**	**Intervention ingredient**	**Theoretical Domains Framework (TDF)**	**Target construct (BCW)**	**Intervention function (BCW)**	**Behaviour Change Technique (using 93 BCT taxonomy v1)**
**Target behaviour: Clinician referral of low risks patients to Penicillin Allergy Testing**						
**Lack of knowledge** about incidence of **incorrect penicillin allergy labels** [QI]	"Penicillin Allergy Testing: Information for general practice" leaflet<br><br>Training	• Provide information on penicillin allergy records and its implications	Knowledge	Psychological capability	Education	4.2 information about antecedents
**Lack of knowledge** of how **accurate** the tests are [QI]	"Penicillin Allergy Testing: Information for general practice" leaflet	• Provide information that the oral challenge test is the gold standard test for penicillin allergy	Knowledge	Psychological capability	Education	4.2 information about antecedents
**Lack of knowledge** of the existence of **allergy services** or what they involve [QI]	"Penicillin Allergy Testing: Information for general practice" leaflet<br><br>Training	• Provide information what the penicillin allergy test involves and where it will take place	Knowledge	Psychological capability	Education	4.2 information about antecedents
**Poor experience** of PAT [QI, RR]	"Penicillin Allergy Testing: Information for general practice" leaflet	• Provide information about why it is important to have correct penicillin allergy labels and what the test involve	Skills<br><br>Behavioural regulation	Physical capability<br><br>Psychological Capability	Training<br><br>Education<br><br>Persuasion	2.7 feedback on outcome of behaviour
**Lack of understanding** PAT procedures and results [QI, RR]	"Penicillin Allergy Testing: Information for general practice" leaflet<br><br>Training	• Provide information about the three stages of penicillin allergy testing and what negative and positive results mean	Knowledge	Psychological capability	Education	4.2 information about antecedents

For peer review only

**Lack of guidelines** in learning about **benefits and risks** of allergy services [QI, RR]	"Penicillin Allergy Testing: Information for general practice" leaflet	• Provide a series of references around penicillin allergy and PAT, including NICE guidelines	Knowledge	Psychological capability	Education	9.1 credible source
**Lack of guidance on referral** [QI, RR]	"Penicillin Allergy Testing: Information for general practice" leaflet<br><br>Training and WIs	• Provide information about the NICE guidelines<br>• Provide a system to refer low risks patients to do testing	Knowledge<br><br>Skills	Psychological capability<br><br>Physical skills	Education<br><br>Training	1.4 action planning<br><br>4.1 instructions on how to perform a behaviour
**Lack of unified approach** for referral of patients to PAT [RR]	"Penicillin Allergy Testing: Information for general practice" leaflet<br><br>Training and WIs	• Provide references on NICE guidelines on referral<br>• Provide a system to refer low risks patients to do testing	Skills<br><br>Social influences	Physical skills<br><br>Social opportunity	Training<br><br>Modelling	1.4 action planning<br><br>4.1 instructions on how to perform a behaviour
**Lack of knowledge** regarding the **oral challenge test** [QI]	"Penicillin Allergy Testing: Information for general practice" leaflet<br><br>Training	• Provide information about the oral challenge test (stage 3 or PAT), why it is done, what the procedure is and how accurate it is	Knowledge	Psychological capability	Education	4.2 information about antecedents
Perception of **overburdening the allergy service** [QI]	"Penicillin Allergy Testing: Information for general practice" leaflet	• Provide information around NICE guidelines in support of PAT	Social/Professional role and identity<br><br>Knowledge	Social opportunity<br><br>Psychological capability	Modelling<br><br>Enablement<br><br>Training	9.1 credible source<br><br>6.3 information about others' approval

For peer review only

**Lack of follow up** after referral [QI]	Pop-up alert<br><br>"Penicillin Allergy Testing: Information for general practice" leaflet<br><br>Training	• Provide information that they will receive confirmation about the patients' results	Environmental Context and resources	Physical opportunity	Environmental restructuring<br><br>Enablement	7.1 prompts/cues<br><br>2.7 feedback on outcome of behaviour
Expectations that **patients may not want to be tested** [RR]	Consultations with patients<br><br>"Penicillin allergy testing: going for a test" booklet	• Provide pre-test booklet to discuss with patients<br>• Provide the opportunity to discuss the benefits of the test during the consultation	Social influences	Social opportunities	Enablement	3.2 social support (practical)<br><br>21.2 restructuring of social environment
Concerns that the test might be **expensive** [QI]	PAT procedures at clinic	• Provide support to referral of patients from colleagues and practice managers<br>• Provide the PAT testing procedures	Social/Professional role and identity	Social opportunities	Enablement<br><br>Modelling	3.2 social support (practical)
Fear of **getting it wrong** and the safety patients [QI]	Training<br><br>PAT procedures in clinic	• Provide support to refer low risk patients to do PAT<br>• Provide evidence of safety of PAT	Social/Professional role and identity<br><br>Beliefs about consequences<br><br>Emotion	Social opportunities<br><br>Reflective motivation<br><br>Automatic motivation	Enablement<br><br>Modelling<br><br>Education<br><br>Persuasion	5.1 information about health consequences<br><br>5.6 information about emotional consequences
Perception that it is **safer to take patients' word for it** [QI]	"Penicillin Allergy Testing: Information for general practice" leaflet	• Provide evidence that only a small percentage of patients who think to be allergic actually are	Beliefs about consequences<br><br>Emotions	Reflective motivation<br><br>Automatic motivation	Education<br><br>Persuasion	5.1 information about health consequences

For peer review only

Fear of **getting it wrong** and **professional ramifications** [*QI]	Training<br><br>PAT procedures in clinic	• Provide support to refer low risk patients to do PAT	Social/Professional role and identity<br><br>Environmental context and resources<br><br>Emotion	Social opportunities<br><br>Physical opportunity<br><br>Automatic motivation	Enablement<br><br>Modelling	3.2 social support (practical)
**Lack of time** during consultation to discuss referral [RR]	Consultation with patients<br><br>Training	• Provide the opportunity to have a consultation with patients to specifically discuss PAT	Environmental context and resources<br><br>Beliefs about capabilities	Physical opportunities<br><br>Reflective motivation	Enablement	12.2 restructuring of social environment
Anticipation that **patients will not want** to have a reaction [RR]	Consultations with patients<br><br>"Penicillin allergy testing: going for a test" booklet	• Provide the opportunity to discuss with patients the risks of taking the test<br>• Provide pre-test booklet with information around the risks of PAT	Social influences	Social opportunity	Enablement	12.2 restructuring of social environment
**Forgetting to discuss** testing [RR]	Consultations with patients<br><br>Training	• Provide the opportunity to have consultations specifically around PAT	Behavioural regulation	Psychological capability	Enablement	3.2 social support (practical)
**Not knowing** that patients had an **allergy** [RR]	Consultations with patients	• Provide the opportunity to discuss their medical history with patients	Knowledge	Psychological capability	Training	2.7 feedback on outcomes of behaviour
**Lack of access** to testing [RR]	PAT testing procedures in clinic<br><br>"Penicillin Allergy Testing: Information for general practice" leaflet	• Provide the information that they can refer low risks patients to undertake PAT<br>• Provide information about where the test will be and what the stages are	Environmental Context and resources<br><br>Knowledge	Physical opportunity<br><br>Psychological capability	Enablement<br><br>Training	5.3 information of social and environmental consequences<br><br>15.1 verbal persuasion of capabilities

For peer review only

**Lack of ability in drawing a conclusion** on allergy status from medical history [RR]	"Penicillin Allergy Testing: Information for general practice" leaflet	• Provide information about the differences between drug reaction and allergy	Knowledge	Psychological capability	Training	4.2 information about antecedents
**Limited knowledge** of drug **allergies** [QI, RR]	"Penicillin Allergy Testing: Information for general practice" leaflet	• Provide information on how an allergy reaction is different than drug reaction	Knowledge	Psychological capability	Training	4.2 information about antecedents
**Poor knowledge** of allergy skin **testing** [RR]	"Penicillin Allergy Testing: Information for general practice" leaflet	• Provide information about skin testing (stage 2 of PAT), what the test will include, how patients will be monitored and how the results will be interpreted in relation to stage 3.	Knowledge	Psychological capability	Training	2.7 feedback on outcome of behaviour
**Lack of confidence** in dealing with allergies [RR]	"Penicillin Allergy Testing: Information for general practice" leaflet	• Provide information about penicillin allergy records and its implication	Beliefs about capabilities	Reflective motivation	Education<br><br>Persuasion	5.1 information about health consequences
Ready **availability of alternate antibiotics** [RR]	"Penicillin Allergy Testing: Information for general practice" leaflet	• Provide information on standards of care and use of broad-spectrum antibiotics	Beliefs about consequences	Reflective motivation	Education<br><br>Persuasion	5.1 information about health consequences
Awareness that **CCG are "cash strapped"** [QI]	PAT procedures at clinic	• Provide allergy testing at the hospital	Environmental context and resources	Environmental Context and resources	Physical opportunity<br><br>Enablement	12.2 restructuring of social environment
Worries about **costs of tests** [*QI]	PAT procedures at clinic	• Provide support in referring patients to PAT	Environmental context and resources	Environmental Context and resources	Physical opportunity<br><br>Enablement	12.2 restructuring of social environment

**Referral takes time** [QI]	PAT procedures at clinic<br>Training and WIs	• Provide guidance about referral and time of responses	Environmental context and resources	Environmental Context and resources	Physical opportunity<br><br>Enablement	4.1 instructions on how to perform a behaviour
**Patients not aware that the allergy label** may have a negative consequences [QI]	Consultations with patients	• Provide the opportunity to discuss the effects of inaccurate allergy labels	Social Influences	Social opportunity	Enablement	12.2 restructuring of social environment
Lack **of support from colleagues** in changing the label [QI]	Training and WIs	• Provide support from lead GPs and colleagues about changing the labels	Social influences	Social opportunity	Modelling	3.2 social support (practical)
**Experience of prescribing an alternative antibiotic** as routine [QI]	Training<br><br>"Penicillin Allergy Testing: Information for general practice" leaflet	• Provide information about how to refer low risks patients<br>• Provide information on the benefits of taking penicillin	Behavioural regulation	Psychological capability	Enablement	4.1 instruction on how to perform a behaviour
*Perception of how **easy finding an alternative antibiotic** is [QI]*	"Penicillin Allergy Testing: Information for general practice" leaflet	• Provide information about the benefits of taking penicillin<br>• Provide information about the effects of broad spectrum antibiotics	Knowledge	Psychological capability	Education<br><br>Enablement	5.1 information about health consequences
*Awareness that patients have been **historically over diagnosed** with penicillin allergy [QI]*	"Penicillin Allergy Testing: Information for general practice" leaflet<br><br>Training	• Provide information that incorrect penicillin allergy records are very common	Knowledge<br><br>Memory	Psychological capability	Education<br><br>Training	4.2 information about antecedents
*Awareness that **records might not be true** because patients do not remember any details of the allergy [QI]*	"Penicillin Allergy Testing: Information for general practice" leaflet	• Provide information about the reasons of incorrect penicillin allergy labels	Beliefs about capability	Reflective motivation	Education	4.2 information about antecedents

Perception of ***importance of referral*** *and penicillin allergy becoming more on the radar [RR]*	"Penicillin Allergy Testing: Information for general practice" leaflet<br><br>Training	• Provide information of the benefit of PAT and appropriate allergy labels	Reinforcement<br><br>Beliefs about consequences<br><br>Memory<br><br>Environmental context and resources	Automatic motivation<br><br>Reflective motivation<br><br>Physical opportunities	Incentivisation<br><br>Education<br><br>Persuasion<br><br>Training	5.1 information about health consequences
Previous medical *and* ***reaction history*** *of patients [QI]*	Consultation with patients	• Provide the opportunity to discuss with patients their medical history and why the test will be relevant for them	Beliefs about capabilities	Reflective motivation	Enablement	12.2 restructuring of social environment
Awareness that it is ***important to prescribe*** *the most suitable antibiotic [QI]*	"Penicillin Allergy Testing: Information for general practice" leaflet<br><br>Training	• Provide information about the benefit of taking penicillin and about antimicrobial resistance	Reinforcement<br><br>Memory	Automatic Motivation<br><br>Psychological capability	Incentivisation<br><br>Training	4.2 information about antecedents
Belief that ***testing would lead to removal*** *of the allergy label [RR]*	"Penicillin Allergy Testing: Information for general practice" leaflet	• Provide information about how to update the patients records after receiving a negative test results<br>• Provide information about prescribing penicillin after a negative test	Reinforcement	Automatic motivation	Incentivisation	1.1 goal setting<br><br>1.4 action planning.<br><br>4.1 instructions on how to perform a behaviour
Belief of ***benefits of removing*** *the allergy label [RR]*	"Penicillin Allergy Testing: Information for general practice" leaflet	• Provide information about how incorrect penicillin allergy labels can be harmful for patients	Reinforcement<br><br>Beliefs about consequences	Automatic motivation<br><br>Reflective motivation	Incentivisation<br><br>Education<br><br>Persuasion	5.1 information about health consequences

For peer review only

Previous experience of referring patients *to PAT [QI]*	Consultations with patients<br><br>PAT procedures in clinic	• Provide the opportunity to explain to patients about PAT procedures and results<br>• Provide the PAT service	Beliefs about capabilities	Reflective motivation	Enablement	15.1 verbal persuasion about capability
Warning patients *about side effects at the time of prescription [QI]*	Consultations with patients<br><br>"Penicillin allergy testing: going for a test" booklet	• Provide the opportunity to discuss any side effects with the patients	Social/Professional role and identity<br><br>Beliefs about capabilities	Social opportunities<br><br>Reflective motivation	Enablement	12.2 restructuring of social environment
NICE guidelines on importance of PAT *and criteria for selecting patients for referral [LR]*	"Penicillin Allergy Testing: Information for general practice" leaflet<br><br>Training	• Provide information on the NICE guidelines on referring to PAT	Knowledge<br><br>Social influences	Psychological Capability<br><br>Social opportunity	Training<br><br>Enablement	9.1 credible source
Belief that ***penicillin allergy is an important*** *issue [RR]*	"Penicillin Allergy Testing: Information for general practice" leaflet	• Provide information on incorrect allergy labels, adverse patient outcomes, effects on antimicrobial prescribing, and on antimicrobial resistance	Reinforcement	Automatic motivation	Education<br><br>Persuasion	5.1 information about health consequences
Perception of ***support of CCG*** *[QI]*	"Penicillin Allergy Testing: Information for general practice" leaflet	• Provide information about the support from colleagues and guidelines for referral	Professional Role	Reflective motivation	Persuasion	9.1 Credible source
Previous experience of removing *or challenging the records *QI]*	PAT procedures at clinic	• Provide the opportunity to refer patients to ultimately change inaccurate labels	Behavioural regulation	Psychological capability	Enablement	15.1 verbal persuasion of capability
**Target behaviour: Clinician prescription of penicillin after negative test result**						

For peer review only

Doubts on **accuracy of allergy labels** in medical records [QI]	"Penicillin Allergy Testing: Information for general practice" leaflet	• Provide information that the oral challenge is the gold standard test for penicillin allergy	Knowledge	Psychological capability	Education<br><br>Persuasion	4.2 information about antecedents
**Worries of harming patients** [QI, RR]	"Penicillin Allergy Testing: Information for general practice" leaflet	• Provide information about safety of patients having penicillin after the test	Emotions<br><br>Beliefs about consequences	Automatic motivation<br><br>Reflective motivation	Education<br><br>Persuasion	5.1 information about health consequences<br><br>5.6 information about emotional consequences
**Lack of knowledge on penicillin** [QI, RR]	"Penicillin Allergy Testing: Information for general practice" leaflet	• Provide information about penicillin allergy and effects on standard of care	Memory	Psychological capability	Training	2.7 feedback on outcomes of behaviour
**System did not distinguish between intolerances and allergies** and may still show alerts [QI]	Pop-up alert WIs and allergy test results letter	• Provide information on the change of allergy status	Environmental context and resources<br><br>Beliefs about capabilities	Physical opportunity<br><br>Reflective motivation	Environmental restructuring<br><br>Enablement	12.5 adding objects to the environment
**Responsibility of changing the system** given to allergy clinic and lack of clarity on how to change them[QI]	Pop-up alert<br><br>Training, WIs and allergy test results letter	• Provide information on who will be responsible in changing the record	Social/professional role and identity<br><br>Beliefs about consequences<br><br>Behavioural regulation	Social opportunity<br><br>Reflective motivation<br><br>Psychological capability	Environmental restructuring<br><br>Enablement	4.1 instructions on how to perform a behaviour
**Lack of confidence** that penicillin could be safely administered [RR]	"Penicillin Allergy Testing: Information for	• Provide information about safety of taking penicillin when the test result is negative	Beliefs about consequences	Reflective motivation	Education persuasion	5.1 beliefs about health consequences

For peer review only

	general practice" leaflet					
**Lack of guidance** on how to interpret the results [QI]	"Penicillin Allergy Testing: Information for general practice" leaflet<br><br>Training, WIs, allergy test results letter	• Provide information on how the results will be presented to the clinicians and patients	Knowledge<br><br>Skills	Psychological capability<br><br>Cognitive skills	Education<br><br>Persuasion<br><br>Training	4.1 instructions on how to perform a behaviour<br><br>2.7 feedback on outcome of behaviour
Perception that **anxious patients may still not want to take penicillin,** and they would believe other people's advice instead [QI]	Consultation with patients<br><br>"Penicillin allergy testing: going for a test" booklet	• Provide the opportunity to discuss the accuracy of the results with patients	Beliefs about capability<br><br>Reinforcement<br><br>Social influences<br><br>Behavioural regulation	Reflective motivation<br><br>Automatic motivation<br><br>Social opportunity<br><br>Psychological capability	Enablement<br><br>Incentivisation	12.2 restructuring of social environment
**Lack of follow** up of the results [QI]	Pop-up alert Allergy test results letter	• Provide an alert when the results are available and the indication of a change of allergy label	Beliefs about consequences<br><br>Behavioural regulation	Reflective motivation<br><br>Psychological capability	Enablement<br><br>Environmental restructuring	12.4 adding object to the environment
Awareness **of different approaches in changing records** in different practices [QI]	Training , WIs, allergy test results letter	• Provide information of the new system in changing labels after PAT and how practices have been involved	Environmental context and resources<br><br>Behavioural regulation	Physical opportunity<br><br>Psychological capability	Environmental restructuring<br><br>Modelling	12.2 restructuring of the social environment<br><br>3.2 social support (practical)

For peer review only

Patients may worry less *about allergies [QI]*	Consultation with patients	• Provide the opportunity to reassure patients	Social influences	Social opportunity	Environmental restructuring	12.2 restructuring of social environment
Recognition that ***test provided evidence*** *that patient is not allergic [QI]*	"Penicillin Allergy Testing: Information for general practice" leaflet<br><br>PAT procedures at clinic	• Provide information about the accuracy of the test and who will perform it	Belief about consequences	Reflective motivation	Education<br><br>Persuasion	5.1 information about health consequences
Awareness that the ***test will allow prescribing first line*** *antibiotics and fight antibiotics resistance [QI]*	"Penicillin Allergy Testing: Information for general practice" leaflet	• Provide information about the effects of accurate penicillin allergy labels	Reinforcement	Automatic motivation	Incentivisation	2.7 feedback on outcomes of behaviour
Perception of ***penicillin allergy testing to be specialist advice*** *and to be objective evidence[QI]*	"Penicillin Allergy Testing: Information for general practice" leaflet	• Provide information of who will perform the test and interpret the results	Belief about consequences<br><br>Reinforcement	Reflective motivation<br><br>Automatic motivation	Education<br><br>Persuasion	5.1 information about consequences
Discussion of the negative test results *to address patients concerns about taking penicillin [QI]*	Consultations with patients	• Provide opportunity to discuss results with patients	Beliefs about capabilities<br><br>Social influences	Reflective motivation<br><br>Social opportunity	Enablement	5.1 beliefs about emotional consequences
Previous experience *of removing or challenging the records [QI]*	WIs, allergy test results letter	• Provide information about how to remove labels	Skills	Cognitive skills	Training	4.1 instructions on how to perform a behaviour
Perception that ***the system is easy to change*** *[QI]*	Pop-up alert, WIs and allergy test results letter	• Provide information about the change of label in the system	Beliefs about capabilities	Reflective motivation	Enablement<br><br>Environmental restructuring	12.5 adding objects to the environment

For peer review only

			Environmental context and resources	Physical opportunity		
Intention to change the label *[QI]*	Training, allergy test results letter	• Provide support and instructions on changing the label	Reinforcement	Automatic motivation	Incentive	2.7 feedback on outcomes of behaviour

Table S1b: Behavioural Analysis Table for the AlABAMA intervention, patient behaviours

For peer review only

**Barriers / *facilitators* to target behaviours**	**Intervention Component/s**	**Intervention ingredient**	**Theoretical Domains Framework (TDF)**	**Target construct (BCW)**	**Intervention function (BCW)**	**Behaviour Change Technique (using 93 BCT taxonomy v1)**
**Patient attendance at Penicillin Allergy Testing (PAT)**						
**Lack of information around the benefits of having access to penicillin**, when patients didn't experience any negative consequences of the allergy label [QI]	"Penicillin allergy testing: going for a test" booklet<br><br>Trial PIS	• Provide evidence on why penicillin is the preferred and best treatment for infections and kills fewer good bacteria;<br>• Provide information that other antibiotics could be less effective, and could increase the risk of "superbug" infections	Beliefs about consequences	Reflective Motivation	Education<br><br>Persuasion	5.1 information about health consequences
**Difficulty for patients to have access to the right antibiotics** if there is a penicillin allergy [QI]	PAT procedures at allergy clinic	• Provide opportunity to undertake allergy testing	Reinforcement	Automatic motivation	Environmental restructuring	12.2 restructuring of physical environment
Concerns related **to attending** the appointment [QI]	Consultations with clinicians;<br><br>Trial PIS	• Provide information about how the appointment will be arranged and that some travel expenses will be provided	Environmental Context and resources	Physical opportunity	Enablement	1.4 action planning

**Barriers / *facilitators* to target behaviours**	**Intervention Component/s**	**Intervention ingredient**	**Theoretical Domains Framework (TDF)**	**Target construct (BCW)**	**Intervention function (BCW)**	**Behaviour Change Technique (using 93 BCT taxonomy v1)**
Concerns whether the **GP will encourage/support** patient to have the test [QI]	Consultations with clinicians<br><br>Trial PIS	• Provide the opportunity to discuss the benefits of the testing with their clinicians	Social influences	Social opportunity	Enablement	3.1 social support (unspecified)
**Unsure about what the test is going to involve** and uncertainty around doses of penicillin given during the test [QI]	"Penicillin allergy testing: going for a test" booklet<br><br>Trial PIS	• Provide information about what a penicillin allergy test would involve, explaining the three stages of the testing	Knowledge;<br><br>Beliefs about consequences	Psychological capability<br><br>Reflective Motivation	Education	2.7 feedback on outcomes of behaviour<br><br>5.1 information about health consequences
**Lack of information about** the **credibility** of PAT [*QI]	Consultations with clinicians	• Provide the opportunity to address any concerns about what PAT is and what other options they may have	Knowledge;<br><br>Beliefs about consequences	Psychological capability<br><br>Reflective Motivation	Education	2.7 feedback on outcomes of behaviour<br><br>5.1 information about health consequences
**Concerns that the procedure is not safe** [QI]	"Penicillin allergy testing: going for a test" booklet<br><br>Trial PIS	• Provide information on the common reactions to PAT during the clinic visit and how patients will be treated by medical staff if they experience any reaction	Beliefs about consequences	Reflective Motivation	Persuasion	5.1 information about health consequences
**Concerns about a possible reaction** during test if they have been told by their clinicians for many years to avoid penicillin [QI]	"Penicillin allergy testing: going for a test" booklet	• Provide reassurance that the allergy test is very safe and that serious reactions are very rare.	Emotions	Automatic motivation	Persuasion	5.1 information about emotional consequences

For peer review only

**Barriers / *facilitators* to target behaviours**	**Intervention Component/s**	**Intervention ingredient**	**Theoretical Domains Framework (TDF)**	**Target construct (BCW)**	**Intervention function (BCW)**	**Behaviour Change Technique (using 93 BCT taxonomy v1)**
**Apprehension about having a test** for patients with a previous severe allergic reaction [QI]	"Penicillin allergy testing: going for a test" booklet<br><br>Trial PIS	• Provide reassurance that the test will be offered only if the nurses and doctors in the allergy clinic think that the patient is low risk	Emotions	Automatic motivation	Persuasion	5.1 information about emotional consequences
Concerns about a reaction that could **worsen their overall state of health** when patients have a chronic co-morbidity [QI]	Consultations with clinicians	• Provide reassurance about the safety of test	Beliefs about consequences;<br><br>Emotions	Reflective Motivation<br><br>Automatic motivation	Education<br><br>Persuasion	5.1 information about health consequences<br><br>5.6 information about emotional consequences
Perception of **skin testing as less frightening** than the oral challenge test [QI]	"Penicillin allergy testing: going for a test" booklet	• Provide information about the oral challenge test; Provide reassurance that the length of time spent at the clinic is long enough to check for any delayed reactions	Beliefs about consequences;<br><br>Emotions	Reflective Motivation<br><br>Automatic motivation	Education<br><br>Persuasion	2.7 feedback on outcomes of behaviour<br><br>5.1 information about health consequences
Patients **concerned about how they would be monitored** during the allergy test and whether doctors would respond quickly in case of reaction [QI]	"Penicillin allergy testing: going for a test" booklet<br><br>Trial PIS	• Provide information on how doctors and nurses will monitor patients during the skin testing and the oral challenge	Beliefs about consequences;<br><br>Emotions	Reflective Motivation<br><br>Automatic motivation	Education<br><br>Persuasion	5.1 information about health consequences

For peer review only

Barriers / *facilitators* to target behaviours	Intervention Component/s	Intervention ingredient	Theoretical Domains Framework (TDF)	Target construct (BCW)	Intervention function (BCW)	Behaviour Change Technique (using 93 BCT taxonomy v1)
**Worries about taking penicillin at home** following the allergy test in clinic and whether there is someone to call in case of reaction [QI]	"Penicillin allergy testing: going for a test" booklet	• Provide information on how the nurse will contact the patients after they have finished taking the penicillin at home to check whether they had any symptoms;<br>• Provide information of possible side effects when taking penicillin at home and that they are mild and do not require treatment<br>• Provide contact details of who to call at the clinic once they experience side effects at home	Beliefs about consequences<br><br>Emotions	Reflective Motivation<br><br>Automatic motivation	Education<br><br>Persuasion	5.1 information about health consequences
Perception that the procedure is **over medicalised** [QI]	"Penicillin allergy testing: going for a test" booklet<br><br>PAT procedures at allergy clinic	• Provide information on the test procedures and on how only the necessary tests will be taken: some of the stages will be skipped based on previous history of reactions and on reaction to the skin test (only necessary tests are carried out)	Beliefs about consequences<br><br>Knowledge	Reflective Motivation	Education<br><br>Persuasion	5.1 information about health consequences
**Lack of knowledge** that allergy status could change over time [RR]	"Penicillin allergy testing: going for a test" booklet	• Provide information about consequences of incorrect allergy label and the benefit of taking the test	Knowledge	Psychological capability	Education	4.2 information about antecedents
Primary care providers **did not inform them about the availability** of PAT [RR]	Consultations with clinicians<br><br>Invitation letter/Trial PIS	• Provide information about penicillin allergy labels and PAT	Knowledge	Psychological capability	Incentivisation<br><br>Education	2.7 feedback on outcome of behaviour

For peer review only

**Barriers / *facilitators* to target behaviours**	**Intervention Component/s**	**Intervention ingredient**	**Theoretical Domains Framework (TDF)**	**Target construct (BCW)**	**Intervention function (BCW)**	**Behaviour Change Technique (using 93 BCT taxonomy v1)**
Always having had a label of penicillin allergy [QI]	"Penicillin allergy testing: going for a test" booklet	• Provide information that once the allergy label is on the patient's medical record is rarely questioned and difficult to remove<br>• Provide information about the percentages of patients who really have a penicillin allergy and the reasons of incorrect allergy label.	Behavioural regulation<br><br>Beliefs about capabilities	Psychological capability<br><br>Reflective Motivation	Education<br><br>Persuasion	5.1 beliefs about health consequences
*Previous **experience of negative consequences** of having a penicillin allergy label and intentions to take penicillin [QI]*	Consultations with clinicians	• Provide an opportunity for patients to discuss with clinicians their previous medical history of penicillin allergy reactions and highlight their personal motivations to undertake the test	Emotion	Automatic motivation	Incentivisation	2.7 feedback on outcomes of behaviour
*Patient **awareness that penicillin is a first-line treatment** for many infections [QI]*	"Penicillin allergy testing: going for a test" booklet<br><br>Trial PIS	• Provide evidence on benefits of taking penicillin compared to broad spectrum antibiotics	Knowledge<br><br>Beliefs about consequences	Psychological capability<br><br>Reflective Motivation	Education	2.7 feedback on outcome of behaviour
*Feeling **relieved because allergy status has been confirmed** [QI]*	"Penicillin allergy testing: going for a test" booklet	• Provide information about the accuracy of allergy labels	Reinforcement	Automatic motivation	Incentivisation	2.7 feedback on outcome of behaviour
*Diagnosis is **an emotionally difficult event** and relief in getting an answer [QI]*	Consultations with clinicians<br><br>"Penicillin allergy testing: a negative test result" booklet	• Provide information on how they will get the results of the testing and provide opportunity to discuss it with GPs<br>• Provide information on the meaning on a negative test, who to inform about the test result and how to do so	Emotions	Automatic motivation	Persuasion	5.3 information about social and environmental consequences

**Barriers / *facilitators* to target behaviours**	**Intervention Component/s**	**Intervention ingredient**	**Theoretical Domains Framework (TDF)**	**Target construct (BCW)**	**Intervention function (BCW)**	**Behaviour Change Technique (using 93 BCT taxonomy v1)**
*Assurance of **access to trained medical staff** at the time of the test [QI]*	"Penicillin allergy testing: going for a test" booklet	• Provide reassurance that all medical staff are fully trained and will act immediately in case of allergy reaction	Beliefs about consequences;<br><br>Emotions	Reflective Motivation<br><br>Automatic motivation	Education<br><br>Persuasion	5.1 information about health consequences
*Perception of being **properly monitored** and of feeling safe during the test [QI]*	"Penicillin allergy testing: going for a test" booklet<br><br>PAT procedures at allergy clinic<br><br>Trial PIS	• Provide reassurance that the medical staff will monitor the patients during the test and will follow up after the patients will have finished the course at home	Beliefs about consequences<br><br>Emotions	Reflective Motivation<br><br>Automatic motivation	Education<br><br>Persuasion	5.3 information about social and environmental consequences
*Feeling **safe if the test takes place on hospital premises** [QI]*	"Penicillin allergy testing: going for a test" booklet<br><br>PAT procedures at allergy clinic<br><br>Trial PIS	• Provide information on where the allergy clinic is.<br>• Attending the PAT at the allergy clinic at the hospital	Knowledge	Psychological capability	Education	2.7 feedback on outcomes of behaviour
*Clear **expectations of what the procedure involves?** [QI]*	Consultations with clinicians;<br><br>"Penicillin allergy testing: going for a test" booklet<br><br>Trial PIS	• Provide information about the procedures of skin testing and oral challenge test	Knowledge	Psychological capability	Education	2.7 feedback on outcomes of behaviour<br><br>4.2 information about antecedents

For peer review only

**Barriers / *facilitators* to target behaviours**	**Intervention Component/s**	**Intervention ingredient**	**Theoretical Domains Framework (TDF)**	**Target construct (BCW)**	**Intervention function (BCW)**	**Behaviour Change Technique (using 93 BCT taxonomy v1)**
*Opportunity for **addressing any concerns** about the procedure [QI]*	Consultations with clinicians	• Provide the opportunity to discuss any concerns about taking the test	Environmental Context and resources;<br><br>Emotions	Physical opportunity<br><br>Automatic motivation	Enablement	12.2 restructuring of social environment
*Belief that PAT provides **valuable information** [RR]*	PAT procedures at allergy clinic;<br><br>"Penicillin allergy testing: a negative test result" booklet	• Provide information about penicillin allergy label<br>• Provide information about the meaning of negative test result and the safety of taking penicillin	Beliefs about consequences	Reflective motivation	Incentivisation	2.7 feedback on outcomes of behaviour
***Interest** in being tested [RR]*	PAT procedures at allergy clinic<br><br>"Penicillin allergy testing: going for a test" booklet	• Provide information about the benefit of taking the test	Reinforcement	Automatic motivation	Incentivisation	2.7 feedback on outcomes of behaviour
***Benefits** of the negative test results [QI]*	"Penicillin allergy testing: going for a test" booklet	• Provide information about the benefits of having a negative test results (being able to take penicillin, being less worries about penicillin, not having to go back to the GP with a reaction)	Reinforcement	Automatic motivation	Incentivisation	2.7 feedback on outcome of behaviour
***Importance of knowing** that the penicillin allergy is correct and that the penicillin allergy test is positive [*QI]*	Consultations with clinicians;<br><br>Invitation letter/Trial PIS	• Provide information about the benefits of accurate allergy labels	Reinforcement	Automatic motivation	Incentivisation	2.7 feedback on outcome of behaviour

For peer review only

Barriers / *facilitators* to target behaviours	Intervention Component/s	Intervention ingredient	Theoretical Domains Framework (TDF)	Target construct (BCW)	Intervention function (BCW)	Behaviour Change Technique (using 93 BCT taxonomy v1)
**Target behaviour: Patient consumption of penicillin when prescribed first line**						
**Perceived risk of clinicians' not prescribing** penicillin even after a negative result [QI]	"Penicillin allergy testing: a negative test result" booklet<br><br>Post-test intervention card	• Provide a card which will inform the clinicians of the change in allergy label<br>• Provide information on how the GP will be informed of the allergy test result	Social Influences	Social opportunities	Enablement	3.2 social support (practical)<br><br>12.2 restructuring of social environment
**Perceived risk of Re-labelling** of patients allergy even after negative test result [QI]	"Penicillin allergy testing: a negative test result" booklet<br><br>Post-test intervention card,<br><br>allergy test result letter	Provide information on how the GP will update the medical record of the patient and other medical systems with negative test results	Social influences	Social opportunities	Enablement	3.2 social support (practical)
Perception of test as **not accurate** [QI, RR]	"Penicillin allergy testing: a negative test result" booklet	Provide information that the test was very accurate in identifying whether the patients have a true penicillin allergy	Beliefs about consequences	Reflective motivation	Education<br><br>Persuasion	5.1 information about health consequences
**Severity** of past allergy reactions [QI]	PAT procedures at allergy clinic	Provide information that nurse or doctors at allergy clinic will only test patient who are perceived as low risk	Beliefs about consequences<br><br>Knowledge	Reflective motivation<br><br>Psychological capability	Education<br><br>Persuasion	4.3 information about antecedents

**Barriers / *facilitators* to target behaviours**	**Intervention Component/s**	**Intervention ingredient**	**Theoretical Domains Framework (TDF)**	**Target construct (BCW)**	**Intervention function (BCW)**	**Behaviour Change Technique (using 93 BCT taxonomy v1)**
**Patient Belief that they are still allergic** to penicillin after a negative text result [RR]	"Penicillin allergy testing: a negative test result" booklet	Provide information about the reliability of the test	Beliefs about consequences	Reflective motivation	Education<br><br>Persuasion	5.1 beliefs about consequences
**Fear of having an allergic reaction** again [RR]	"Penicillin allergy testing: a negative test result" booklet	Provide information about low risks of having an allergic reaction in the future	Emotion	Automatic Motivation	Education<br><br>Persuasion	5.6 beliefs about emotional consequences
**Lack of confidence** that penicillin could be safely administered [RR]	"Penicillin allergy testing: a negative test result" booklet	Provide information about the meaning of test results and which antibiotics are now safe to use	Beliefs about consequences	Reflective motivation	Education<br><br>Persuasion	5.1 beliefs about consequences
**Lack of knowledge of which antibiotics they could take** safely [RR]	"Penicillin allergy testing: a negative test result" booklet	Provide a list of antibiotics that patients with a negative test result can take	Knowledge	Psychological capability	Education	4.2 information about antecedents
**Distrust in health care providers'** advice if they don't prescribe penicillin even after the negative test result [RR]	Post-test intervention card	Provide card to show to health care professionals which gives details about the allergy test completed and explains that the patient had a negative test result	Reinforcement	Automatic motivation	Environmental restructuring	12.2 restructuring of social environment<br><br>12.5 adding objects to the environment
**Lack of knowledge about how to interpret skin test results** [RR]	Post-test intervention card	Provide explanation of the meaning of negative test results and what to do the next time they will need antibiotics	Knowledge	Psychological capability	Education	4.2 information about antecedents
**No differences in concerns** between patients who were investigated through skin test or oral test [RR]	"Penicillin allergy testing: a negative test result" booklet	Provide information about the accuracy of the test completed	Beliefs about consequences	Reflective motivation	Education<br><br>Persuasion	5.1 beliefs about consequences

**Barriers / *facilitators* to target behaviours**	**Intervention Component/s**	**Intervention ingredient**	**Theoretical Domains Framework (TDF)**	**Target construct (BCW)**	**Intervention function (BCW)**	**Behaviour Change Technique (using 93 BCT taxonomy v1)**
*Perception of the test as **definitive answer** and proof of allergy status [QI]*	PAT procedures at allergy clinic;<br><br>"Penicillin allergy testing: a negative test result" booklet<br><br>Post-test intervention card<br><br>allergy test result letter	• Provide the test results from the allergy clinic<br>• Provide information on the meaning of the negative test result and information about the accuracy of the test<br>Provide information about reliability of test	Reinforcement	Automatic motivation	Environmental restructuring	2.7 feedback on outcomes of behaviour
*Perception to have undergone a **thorough testing procedure** [QI]*	PAT procedures at allergy clinic	Monitoring of the patients by the nurse or doctor at allergy clinic who will provide medical assistance when needed	Reinforcement	Automatic motivation	Environmental restructuring	12.2 restructuring of the social environment
***Lack of reaction** following the test [QI]*	PAT procedures at allergy clinic<br><br>"Penicillin allergy testing: going for a test" booklet	Provide information about the type of reactions and their indication on the state of the allergy	Reinforcement	Automatic motivation	Environmental restructuring	12.1 restructuring of the physical environment

S2. Think-aloud topic guides for clinicians and patients

"Penicillin Allergy Testing: Information for general practice" leaflet

In this feedback session, I am interested in what you think about when you read the information provided in the booklets. I would like you to think aloud as you read through each section, if you have any comments. There is no right or wrong answers. So when you read a section of the booklet, if you could tell me what thoughts come to your mind when you read it.

1. Looking at the Penicillin allergy" and "Incorrect penicillin allergy records are very common what did you think after reading it? Did you think it was comprehensive/ new information/ useful information
2. Going through the section on "Incorrect penicillin allergy records can be harmful for patients what did you think while reading it? What did this section tell you? Did you find it convincing?
3. Looking at the section "Penicillin allergy testing -What does the test involve?", was it clear to you what the testing involves? Would you feel confident in explaining the process to patients?
4. What do you think about the section: "How accurate is the test"? How do you understand this section? Is there anything unclear?
5. What do you think about the section: "Are there any risks to patients having the test?" Do you feel reassured by this section? Would you feel confident to refer patients for testing based on this information?
6. What do you think about the section: "How should I discuss the test with patients? "? Do you think this is an important section? Is the description clear?
7. What do you think about the section: "Updating medical records"? Is it clear what you need to do?
8. What do you think about the section: "Prescribing penicillin after a negative test"? Would you feel confident to prescribe penicillin after reading this section?

- What are your overall thoughts on the leaflet?
- How can this leaflet be improved?
- What will stick in your mind from this leaflet?
- How easy or difficult was it to read/ understand this leaflet?
- Is there anything which you think is missing?
-

Intervention Card

- What do you think about this card?

For peer review only

- Can you tell me how do you understand the last sentences: that the test was negative which means that the patient is not allergic
- What does: with the same risk of allergy as the general population mean to you?

Can you tell me what you would do if a patient came with this card

"Penicillin allergy testing: going for a test" booklet

In this feedback session, I am interested in what you think about when you read the information provided in the booklets. I would like you to think aloud as you read through each section, if you have any comments. There is no right or wrong answers. So when you read a section of the booklet, if you could tell me what thoughts come to your mind when you read it.

9. Looking at the front cover the writing and the pictures, what did you think this leaflet was going to be about?
10. Looking at page 3, with a list of contents, what did you think after reading it? Did you at that point think that it was comprehensive?
11. Going through the first section: "How do I know if I am allergic to penicillin", what did you think while reading it? What did this section tell you? Why do you think this section is here?
    Specifically, there is some numerical information provided here about how many people are allergic etc, can you tell me in your own words what you understand by this paragraph?
12. Going through the next paragraph on why is it important for people to have a penicillin allergy test, again what did you think while reading it? Were you convinced by the argument? How do you understand this section?
13. Going through the next section: "what does a penicillin allergy test involve", what do you think of this section? Could you talk me through what you think you would have to do if you had this test? Is there anything unclear? Is there anything missing? What do you think about the test and what is required of you.
14. Going through the next section: "Are there any risks", what was going through your head when you were reading that? What were you feeling when you have read that? How would you feel about going for a test after reading this section? Specifically, this section tell you what symptoms people may get- what did you think while reading this section? What is this section telling you? How would you put this section in your own words?

- What are your overall thoughts on the leaflet?
- How can this leaflet be improved?
- What will stick in your mind from this leaflet?

For peer review only

- How easy or difficult was it to read/ understand this leaflet?

"Penicillin allergy testing: a negative test result" booklet

1. Looking at the front cover the writing and the pictures, what did you think this leaflet was going to be about? How do you understand the heading: Negative test result? What did you think about the picture?
2. Looking at the table of contents, what did you think after reading it? Did you at that point think that it was comprehensive?
3. Going through the next section: "what does a negative test result mean", what was going through your head when you were reading that? How do you understand this section? What do you think about the picture used here?
4. Going through the next section: "Is it safe for me to now take penicillin", what were you thinking while going through this section?
5. Going through the next section: "What should I do now?" How do you understand this section? What were you thinking reading this section? Can you tell me in your words what you are asked to do?
6. Going through the next section: "What should I do next time I have an infection?", how do you understand this section?

- What are your overall thoughts on the leaflet?
- How can this leaflet be improved?
- What will stick in your mind from this leaflet?
- How easy or difficult was it to read/ understand this leaflet?

Intervention Card

- What do you think about this card?
- Can you tell me how do you understand the last sentences: that the test was negative which means that the patient is not allergic
- What does: with the same risk of allergy as the general population mean to you?
- Can you tell me in what situations you think you may use it?

For peer review only

S3. Table of changes: "Penicillin Allergy Testing: going for a test" booklet

Suggested changes	Changes made	Reasons for change	Priority for change
**Front page**			
The image on the cover of the booklet must be changed as it does not relate to what happens during penicillin allergy testing; also the blood samples on the GP desk look scary.	A new image was selected showing a more friendly looking consultation between patient and GP without any medical equipment	EO	Must do
The logos could be put at the bottom of the cover pager (rather than at the top) as they distract from the image)	The logos are on the bottom of the page	EO	Could do
The participants suggested making the booklets even easier to follow by adding text boxes throughout to separate big chunks of text.	The booklet was made more visually appealing	EO	Could do
**How can this booklet help me?**			
The contents page looks a bit dense on the page. The participants did not like the "chemical symbols" image in the background design.	• Better use of the space on the contents page was made and the text more was spread across the page.<br>• The introduction to the booklet was put in a separate box to make it look more manageable. Chemical symbols image were removed.	EO	Should do

For peer review only

Suggested changes	Changes made	Reasons for change	Priority for change
**How do I know if I am allergic to penicillin?**			
The participants suggested putting the last sentence of this section: "A penicillin allergy test is the safest way to find out whether or not you are allergic to penicillin" in bold.	The last sentence was put in bold	EO, QI	Must do
Statistics about the prevalence of allergy were not always understood by the participants, as the participants often thought that 1 in 10 people are allergic. They wanted a more visual presentation of this key information	An infographic was created showing how many people think they are allergic and how many people actually are allergic. The image of a GP taking blood pressure was replaced by the infographic.	EO	Should do
This section looks also a bit dense on the page	The text was spread across to the following page	EO	Should do
**Why is it important for people to have a penicillin allergy test?**			
Participants were unsure what narrow and broad spectrum antibiotics were and did not recognise MRSA abbreviation.	• The reference to narrow and broad spectrum antibiotics was removed<br>• The full name for MRSA was presented	RT, CT	Must do
Participants did not always know that penicillin is more than one antibiotic. This message should be made clearer.	An additional sentence was added to provide information that penicillin includes a number of different antibiotics	EO, QI	Must do

For peer review only

Suggested changes	Changes made	Reasons for change	Priority for change
Booklet 2 mentioned that penicillin is good at treating throat infections- this information was missing from this booklet.	A sentence was added to explain that “antibiotics are very useful to treat many infections, including ear, skin, throat and chest infections”	EO, QI	Must do
The information was slightly repetitive and perhaps the section should start with the strongest message (which participants thought was that penicillin are effective).	• The section was made more concise.<br>• The first paragraph includes information on what penicillins are, what they are used for, and that they are considered the best treatment for infections	EO	Should do
Other sentences in this section, for example: “other antibiotics may not be as effective” could also be put in bold.	The sentence was put in bold	EO, QI	Should do
The section looks a bit dense.	The text was spread across the previous page	EO	Should do
**What does a penicillin allergy test involve?**			
Participants wanted to know if they can call the allergy clinic and whether they will be provided a number and what details the doctor or nurse will be checking (Why they would not have to be checked in person?).	It reads: “you will be provided with details of who to call if you have any symptoms or concerns”	RT, CT, QI	Must do

For peer review only

Suggested changes	Changes made	Reasons for change	Priority for change
Participants wanted to have reassurance that 3 days would be enough to detect delayed reactions.	It reads: "this is long enough to check for any delayed reactions"	RT, CT, QI	Must do
Participants suggested making it clearer that the clinic is in the hospital.	This sections starts with "Penicillin allergy testing takes place in a hospital, in a specialist allergy clinic". The sentence was put in bold	RT, CT, QI	Must do
Participants had queries about symptoms they could experience after three days of taking penicillin as the booklet said: "mild and mostly side effects"; they wanted clarification whether these could be allergic reaction symptoms	It reads: "these symptoms are generally mild and do not require treatment"	RT, CT, QI	Must do
Participants did not always like the phrase "like a mosquito bite" (skin testing stage) as some people may have an "unpleasant reaction to mosquitos".	No change made – comment from one participant, felt by clinical team to be best way to describe and usual term used in current NHS leaflets.	RT, CT	Could do
There is a lot of text on one page	The descriptions of the test was spread over two pages	EO	Should do
A diagram alongside the stages of the test could be presented to make it even clearer	A diagram was provided for the three stages of testing and the description of the test was spread over two pages	EO	Could do
**Are there any risks to having the test?**			

For peer review only

Suggested changes	Changes made	Reasons for change	Priority for change
The participants wanted to have a separate paragraph on what can happen during the test and what can happen during 3 days of taking penicillin at home.	There are two paragraphs, one on what could happen during the clinic visit, and one about what could happen at home	RT, CT	Must do
They wanted clarification whether the listed symptoms for taking penicillin at home: "sickness, a mild, small rash, nausea, upset stomach, or other mild symptoms" were symptoms of an allergic reaction; this was especially confusing as feeling sick was also listed as part of the allergic reaction when taking penicillin in the clinic (you might feel sick, itchy, develop hives ('nettle rash'), swelling, a fast heartbeat, dizziness or difficulty breathing.	It reads: "Once you go home, it is possible you may develop mild symptoms such as sickness, a mild, small rash, nausea, upset stomach, or other mild symptoms. It is extremely unlikely that you will have any serious symptoms, as these would have happened when you took penicillin during the clinic visit"	RT, CT, QI	Must do
The participants suggested putting the phrase: "extremely unlikely" in bold ("It is extremely unlikely that you will have any serious symptoms, as these would have happened when you took penicillin during the clinic visit".	The phrase was put in bold	EO	Could do

Note: EO: patient; RT: research team; CT: clinical team; QI: qualitative interviews

For peer review only

S4. Full description of the AlABAMA (ALlergy AntiBiotics And Microbial resistAnce) Intervention package in accordance with the Template for Intervention Description and Replication (TIDieR) framework.

- **1. BRIEF NAME:** AlABAMA (ALlergy AntiBiotics And Microbial resistAnce) Intervention package
- **2. WHY:** Penicillins are the most prescribed antibiotics and they are used as first-line therapy for many infections. Side effects and symptoms related to the infections are commonly mislabelled as allergy. Around 6% of people think that they are allergic to penicillin but fewer than 10% of these patients are truly allergic. As a consequence a significant proportion of the population have restricted access to highly effective penicillins, and are prescribed broad-spectrum antibiotics instead, which may be less effective and could increase the risks of acquiring multi-drug resistant bacteria. The goal of AlABAMA is to introduce a “pre-emptive” penicillin allergy assessment pathway (PAAP) for patients who are more likely to receive antibiotics that could impact antibiotic prescribing, have patients benefits, limit antimicrobial resistance (AMR) infections and deliver NHS cost savings. The AlABAMA approach will develop a “one stop shop” single Penicillin Allergy Test (PAT) at the hospital clinic for specialist immunology assessment (skin testing and/or oral challenge test).
- **3&4: WHAT:** AlABAMA is a complex behaviour change intervention which targets prescribers in general practice and low risk patients with a penicillin allergy record. See Table 2 for an overview of the key intervention components. For more details about the specific behaviour change techniques used, refer to the behavioural analysis.
- **5. WHO PROVIDED**: The AlABAMA research team will provide intervention materials to general practices taking part in the feasibility and main trial. Practices will be responsible for local implementation by the practice manager(s). GPs will have consultations with patients to check eligibility for entering the trial.
  Research nurses at the hospital will be responsible for sending the two intervention booklets to patients in the intervention arm by post. Research nurses at the immunology hospital clinic will deliver Stage 1, Stage 2 and/or stage 3 of the PAT.

For peer review only

- **6. HOW**: The intervention will be delivered mainly by written information for prescribers and for patients in the intervention arm. The site training will be delivered to all the GP leads and the practice manager(s) in one session per practice. Prescribers will receive information by email. Patients randomised to the PAAP intervention arm will receive the "Penicillin Allergy Testing: going for a test" by post. Patients will complete Stage 1 and Stage 2 (skin test) and /or Stage 3 (oral challenge test) in a single hospital visit which lasts around 3 hours. Patients with a negative PAT result will receive the "Penicillin Allergy Testing: a Negative Test Result" booklet by post. Prescribers will receive the update about the penicillin allergy test result by post and electronically.
- **7. WHERE**: AlABAMA is intended for implementation in general practices, where the site training and patient consultation also take place. The PAT takes place at the immunology clinic of a local hospital. Written information for clinicians is provided electronically subsequently to the site training. Written information for patients is sent by post by research nurses at the hospital.
- **8. WHEN and HOW MUCH**: Site training is provided by the AlABAMA research team prior to the screening and eligibility assessment of patients. Patients randomised to the PAAP trial arm will be sent the "Penicillin Allergy Testing: going for a test" booklet with their hospital appointment letter. Patients randomised to the control arm will receive usual clinical care. All patients completing the PAT will receive a letter with the test result after their appointment. Patients who have tested negative will receive the "Penicillin Allergy Testing: a Negative Test Result" booklet and Intervention card by post. Practices will be informed of the test result and instructed to update the patients' electronic health record.
- **9. TAILORING**: The implementation of behavioural and clinical elements of the AlABAMA intervention package will be implemented in all GP practices and local immunology clinics participating in the trial.
- **10. MODIFICATIONS**: Modifications were made to the intervention materials following qualitative feedback from the feasibility study.
- **11-12. HOW WELL**: The intervention is being tested in a feasibility trial in 8 general practices. It will then be tested in a randomised control trial across 70 GP practices.

For peer review only

S5 GUIDED – a guideline for reporting for intervention development studies.

**Item description**	**Page in manuscript where item is located**
1. Report the context for which the intervention was developed.	4
2. Report the purpose of the intervention development process.	5
3. Report the target population for the Intervention development process.	4
4. Report how any published intervention development approach contributed to the development process	5
5. Report how evidence from different sources informed the intervention development process.	6
6. Report how/if published theory informed the intervention development process.	11
7. Report any use of components from an existing intervention in the current intervention development process.	5
8. Report any guiding principles, people or factors that were prioritised when making decisions during the intervention development process.	9
9. Report how stakeholders contributed to the Intervention development process.	8
10. Report how the intervention changed in content and format from the start of the intervention development process.	14-15
11. Report any changes to interventions required or likely to be required for subgroups.	15-17
12. Report important uncertainties at the end of the intervention development process.	21-22

For peer review only

13. Follow TIDieR guidance when describing the developed intervention.	18
14. Report the intervention development process in an open access format.	1

For peer review only

BMJ Open

Developing a behavioural intervention package to identify and amend incorrect penicillin allergy records in UK general practice and subsequently change antibiotic use

Journal:	*BMJ Open*
Manuscript ID	bmjopen-2019-035793.R2
Article Type:	Original research
Date Submitted by the Author:	29-Jul-2020

Complete List of Authors:	Santillo, Marta; Oxford University, Nuffield Department of Primary Care Health Sciences<br>Wanat, Marta; Oxford University, Nuffield Department of Primary Care Health Sciences<br>Davoudianfar, Mina; University of Oxford, Clinical Trials Unit, Nuffield Department of Primary Care Health Sciences<br>Bongard, Emily; University of Oxford, Nuffield Department of Primary Care Health Sciences<br>Savic, Sinisa; University of Leeds Faculty of Medicine and Health<br>Savic, Louise; University of Leeds Faculty of Medicine and Health<br>Porter, Catherine; University of Leeds, Healthcare Associated Infection Group<br>Fielding, Joanne; University of Leeds, Healthcare Associated Infection Group<br>Butler, Christopher C.; University of Oxford,<br>Pavitt, Sue; University of Leeds<br>Sandoe, Jonathan; University of Leeds Faculty of Medicine and Health<br>Tonkin-Crine, Sarah ; University of Oxford, Nuffield Department of Primary Care Health Science

**Primary Subject Heading**:	General practice / Family practice
Secondary Subject Heading:	Infectious diseases
Keywords:	GENERAL MEDICINE (see Internal Medicine), INFECTIOUS DISEASES, Allergy < THORACIC MEDICINE

SCHOLARONE™
Manuscripts

I, the Submitting Author has the right to grant and does grant on behalf of all authors of the Work (as defined in the below author licence), an exclusive licence and/or a non-exclusive licence for contributions from authors who are: i) UK Crown employees; ii) where BMJ has agreed a CC-BY licence shall apply, and/or iii) in accordance with the terms applicable for US Federal Government officers or employees acting as part of their official duties; on a worldwide, perpetual, irrevocable, royalty-free basis to BMJ Publishing Group Ltd ("BMJ") its licensees and where the relevant Journal is co-owned by BMJ to the co-owners of the Journal, to publish the Work in this journal and any other BMJ products and to exploit all rights, as set out in our licence.

The Submitting Author accepts and understands that any supply made under these terms is made by BMJ to the Submitting Author unless you are acting as an employee on behalf of your employer or a postgraduate student of an affiliated institution which is paying any applicable article publishing charge ("APC") for Open Access articles. Where the Submitting Author wishes to make the Work available on an Open Access basis (and intends to pay the relevant APC), the terms of reuse of such Open Access shall be governed by a Creative Commons licence – details of these licences and which Creative Commons licence will apply to this Work are set out in our licence referred to above.

Other than as permitted in any relevant BMJ Author's Self Archiving Policies, I confirm this Work has not been accepted for publication elsewhere, is not being considered for publication elsewhere and does not duplicate material already published. I confirm all authors consent to publication of this Work and authorise the granting of this licence.

For peer review only

Developing a behavioural intervention package to identify and amend incorrect penicillin allergy records in UK general practice and subsequently change antibiotic use.

Corresponding author: Marta Santillo, Nuffield Department of Primary Care Health Sciences, University of Oxford, Radcliffe Observatory Quarter, Woodstock Road, Oxford, OX2 6GG, marta.santillo@phc.ox.ac.uk, 01865617935

Santillo, M. [1], Wanat, M. [1], Davoudianfar, M. [1] , Bongard, E. [1], Savic, S. [2] Savic, L. [2], Porter, C. [3], Fielding, J. [3], Butler C. C. [1], Pavitt, S. [4], Sandoe, J. [3] , Tonkin-Crine, S. [1,5] On behalf of the AlABAMA team.

[1] Nuffield Department of Primary Care Health Sciences, University of Oxford, Radcliffe Observatory Quarter, Woodstock Road, Oxford, OX2 6GG , UK

[2] Faculty of Medicine and Health, University of Leeds and Leeds Teaching Hospitals NHS Trust, Leeds LS1 3EX, UK

[3] Healthcare Associated Infection Group, University of Leeds and Leeds Teaching Hospitals NHS Trust, Leeds LS1 3EX, UK

[4] Dental Translational and Clinical Research Unit, Faculty of Medicine and Health, Worsley Building, Clarendon Way, University of Leeds, LS2 9LU, UK

[5] NIHR Health Protection Research Unit in Healthcare Associated Infections and Antimicrobial Resistance, University of Oxford, UK

SHORT RUNNING TITLE: Intervening to amend incorrect penicillin allergy records in general practice

KEYWORDS: intervention development, penicillin allergy testing, general practice

Words: 4548/4000

For peer review only

For peer review only - http://bmjopen.bmj.com/site/about/guidelines.xhtml

ABSTRACT

Words: 297/300

Objectives To develop a behavioural intervention package to support clinicians and patients to amend incorrect penicillin allergy records in general practice. The intervention aimed to: 1) support clinicians to refer patients for penicillin allergy testing (PAT), 2) support patients to attend for PAT and 3) support clinicians and patients to prescribe or consume penicillin, when indicated, following a negative PAT result.

Methods Theory-based, evidence-based and person-based approaches were utilised in the intervention development. We used evidence from a rapid review, two qualitative studies, and expert consultations with the clinical research team to identify the intervention 'guiding principles' and develop an intervention plan. Barriers and facilitators to the target behaviours were mapped to behaviour change theory in order to describe the proposed mechanisms of change. In the final stage, think-aloud interviews were conducted to optimise intervention materials.

Results The collated evidence showed that the key barriers to referral of patients by clinicians were limited experience of referral and limited knowledge of referral criteria and PAT. Barriers for patients attending PAT were lack of knowledge of the benefits of testing and lack of motivation to get tested. The key barriers to the prescription and consumption of first-line penicillin following a negative test result were patient and clinician beliefs about the accuracy of PAT and whether taking penicillin was safe. Intervention materials were designed and developed to address these barriers.

Conclusions We present a novel behavioural intervention package designed to address the multiple barriers to uptake of PAT in general practice by clinicians and patients. The intervention development details how behaviour change techniques have been incorporated to hypothesise how

For peer review only

the intervention is likely to work to help amend incorrect penicillin allergy records. The intervention will go on to be tested in a feasibility trial and randomised controlled trial in England.

ARTICLE SUMMARY

Strengths and limitations of this study

- Intervention development was conducted using a systematic and iterative process.
- Evidence-, theory and person-based approaches were used which consisted of three iterative stages: collating the evidence on views and experiences of PAT, intervention planning informed by theory and optimisation of intervention materials.
- These approaches were used to increase the feasibility and acceptability of the intervention to its target users (GPs and patients).
- This approach clearly maps how the intervention components are hypothesized to change clinician and patient target behaviours.
- The views of other general practice prescribers (such as nurse prescribers and pharmacists) should be considered in future research.

For peer review only

BACKGROUND

Incorrect penicillin allergy records are common, as side effects and symptoms of illness can be mistaken for allergic reaction symptoms [1]. About 6% of the UK general practice population has a record of a penicillin allergy but fewer than 10% of these patients are likely to be truly allergic [2]. Penicillin allergy refers to an allergy to a whole group of antibiotics (penicillins) which are first line antibiotics for many common infections. As a result of incorrect penicillin allergy labels, a significant portion of the population is denied effective antibiotics. Moreover, penicillin allergy records are also linked to antimicrobial resistance (AMR): evidence suggests that patients with a penicillin allergy label are more likely to be prescribed broad spectrum antibiotics and to acquire antibiotic resistance infections [3,4].

Penicillin allergy testing is already provided in the NHS in specialist clinics [5] and offers the opportunity to confirm or discount a penicillin allergy label. This service is only available to a subset of patients following NICE guidance [6]. Current assessment is performed over two clinic visits.

The AlABAMA (Allergy Antibiotics and Microbial resistance) study aims to develop a behavioural intervention package for UK general practice to effectively amend incorrect penicillin allergy records. The intervention package is designed to target both general practice clinicians and patients with a suspected incorrect penicillin allergy record. It introduces a pre-emptive 'penicillin allergy assessment pathway' (PAAP) which targets patients assessed as "low risk" of true allergy, who are not at risk of anaphylaxis or other severe adverse reactions, and it aims to streamline the test process by undertaking patient history screening in general practice (Stage 1) and introducing an efficient one-stop procedure at a hospital clinic for the penicillin allergy test (PAT). The test includes either a skin test (ST), testing penicillin solution on the forearm (Stage 2), and oral challenge test (OCT), taking doses of penicillin solution over time (Stage 3), or just OCT depending on the individual patient. Following the PAT, patients and practices would receive confirmation of a patient's allergy

For peer review only - http://bmjopen.bmj.com/site/about/guidelines.xhtml

status. The AlABAMA intervention will be trialled in a national randomised controlled trial in winter 2020-2021. It will test how corrections of incorrect penicillin allergy records and subsequent penicillin prescribing influence recovery and patients taking antibiotics for infections.

This paper describes the planning, development and optimisation of the AlABAMA PAAP intervention package.

METHODS AND RESULTS

Intervention planning methodology

We followed an integrated approach to intervention development that combines theory-, evidence- and person-based approaches [7-9]. This approach has been successfully used in the development of a variety of behavioural interventions including for reduction of antibiotic prescriptions in European general practice [10].

Patient and Public Involvement (PPI)

The AlABAMA programme grant involved PPI members, as co-applicants, and study advisors from the start so research questions were informed by their input. Results of all work from the AlABAMA programme will be disseminated with the help from our PPI co-apps and our existing PPI networks towards the end of the programme grant.

The AlABAMA intervention package was developed in three stages described below. The methods and key results for each stage are presented below.

For peer review only - http://bmjopen.bmj.com/site/about/guidelines.xhtml

Stage 1: Collating and analysing evidence

Rapid review methods

The full rapid review is available elsewhere [11]. Rapid review is a form of synthesis which supports the review of existing evidence in a timely manner [12]. It aimed to explore clinicians' and patients' views and experiences of penicillin allergy testing services.

Results

The review identified only two studies which reported patients' views of PAT. The first study sought patients' views on undertaking a three-step testing procedure consisting of prick testing, intradermal testing and oral challenge[13] . It found that patients thought that PAT provided a valuable medical information; however, this was measured using a single-item questionnaire ("Do you think undergoing testing for penicillin allergy provides important information for your penicillin history"). The second study recruited patients who attended general allergy clinic and were given a brief 5-question survey examining their views of PAT[14] . This study found that patients were not aware of penicillin allergy testing and had limited understanding of penicillin allergy, but also expressed their interest in being tested.

The review also identified six studies on clinicians' views of PAT[13, 15-19]three of which specifically focused on barriers and facilitators to using PAT [13, 17, 19]. One of the studies included both patient and clinician views [19]. Clinicians reported several barriers to referring patients for PAT. These included difficulties establishing the allergy history, lack of knowledge on referral processes and organisational pressures making allergy testing a low priority [13,17,19]. A number of clinicians and patients reported being reluctant to prescribe or consume penicillin after a negative PAT result.

Qualitative methods

Full details of the qualitative work are available elsewhere[20]. Two qualitative studies were undertaken by MW; one interviewing 19 general practitioners and the second interviewing 31 patients with a penicillin allergy record (16 with previous experience of PAT). The aim was to identify

For peer review only - http://bmjopen.bmj.com/site/about/guidelines.xhtml

clinicians' and patients' views on the barriers and facilitators for PAT and antibiotic use after a negative test. Semi-structured interviews were conducted over the phone by an experienced qualitative researcher (PhD qualified) with substantial previous experience of conducting qualitative research. Clinicians were identified in general practices and by the local microbiology services. Patients were identified from a general adult hospital allergy clinic and from general practices in the same geographical area. Interview topic guides were informed by existing literature which indicated potential barriers and facilitators to target behaviours.[20] Written informed consent was obtained from all participants. An inductive thematic analysis approach was used to analyse data[21].

Results

Clinicians reported that they often felt that penicillin allergy records were incorrect however reported uncertainty about how to identify patients with true penicillin allergy and were reluctant to amend medical records without objective evidence. Penicillin allergy status was not seen to be a major problem in general practice due to the availability of alternative antibiotics and clinicians struggled to identify the risks of incorrect allergy records. Clinicians were seen to differ in their consultation styles when speaking to patients about their antibiotic prescribing decisions and allergy status. They reported lack of experience of PAT services and the need for more information on referral criteria. Regarding the process of changing a patients' record after a negative test result, clinicians reported being happy to update medical records on directions from secondary care but were unsure who was responsible for making sure that patients understood allergy test results.

The majority of patients who were motivated to get tested had experienced a negative consequence of having a penicillin allergy label (such as limited availability of antibiotics they could use). Patients reported concerns about the possibility of having an allergic reaction during PAT; the degree of the severity of their previous reactions affected how apprehensive they were about the test. Moreover, when the test was perceived as more invasive, e.g. the oral challenge test compared to the skin test, patients reported being more concerned about PAT. Patients were also concerned about how much

For peer review only - http://bmjopen.bmj.com/site/about/guidelines.xhtml

they would be monitored during the test and highlighted the importance of being informed, kept safe and observed by qualified professionals. Some patients reported being unsure about a negative test result and feeling anxious about taking penicillin if prescribed, as they doubted whether the test result was accurate.

Expert Discussions

In addition to our core team (which included three psychologists), the wider clinical research team included a consultant immunologist, a consultant microbiologist, a consultant anaesthetist, a general practitioner and professors with expertise in applied health research. As part of this first stage we consulted our wider clinical research team using monthly tele-conferences and emails to gain their feedback on several aspects of the intervention development.

Results

The expert discussions informed the interpretation of the evidence collated in the rapid review and the qualitative studies, the development of early iterations of the intervention materials, and the development of the initial intervention plan and components.

Stage 2: Intervention planning and development

Creating guiding principles

In line with the person-based approach[7] brief guiding principles were created to be consulted through the whole intervention development process. This ensured that the intervention met the original objectives. As a first step to creating guiding principles, characteristics and behavioural needs of the target users were identified based on the findings of the rapid review, qualitative

For peer review only - http://bmjopen.bmj.com/site/about/guidelines.xhtml

interviews and expert discussions. The second step involved identification of intervention objectives and key design features which would address these needs.

Results

Table 1 presents the AlABAMA guiding principles. These focused on increasing confidence to refer and attend for PAT and increasing motivation to prescribe/consume penicillin following a negative PAT result. Guiding principles also included increasing clinician confidence in discussing penicillin allergy with patients and improving communication between primary and secondary care about penicillin allergy status. Lastly the intervention needed to present the PAAP as reliable and trusted and provide accessible and easy to use materials for clinicians and patients.

Table 1: Guiding Principles for the AlABAMA intervention package.

PAAP =Penicillin Allergy Assessment Pathway; PAT = Penicillin Allergy Testing

**Intervention design objectives**	**Key features**
To present the PAAP as a reliable and trusted approach to confirm allergy status	• Present the PAAP as a trusted/scientific/official way to get a confirmed PAT result, for both clinicians and patients
To increase clinician's confidence in referring patients, and patients' motivation to attend, for PAT	• Provide evidence on the potential harms of an incorrect penicillin allergy records and the process of testing in the Information Pack<br>• Provide opportunity to address patients' concerns about potential benefits and risks of testing by both clinicians and allergists during consultations before the allergy test and during the appointment at the immunology clinic

For peer review only

For peer review only - http://bmjopen.bmj.com/site/about/guidelines.xhtml

Intervention design objectives	Key features
To motivate clinicians/patients to prescribe/take penicillin following a negative PAT test result	• Provide information to clinicians about the clinical meaning of a test result and its implication as part of the Information Pack<br>• Provide information to patients about the PAT (process) and the potential benefits of being able to take penicillin in the Pre-test Intervention Booklet
To increase clinicians' ability to discuss PAT with patients	• Provide training for general practice clinicians about penicillin allergy and PAT, including its accuracy, implications, benefits
To improve communication between primary and secondary care so that allergy status records are correct	• Provide a clear and consistent approach to de-labelling with support from colleagues in secondary care and preventing re-labelling in the future<br>• Provide information about who is responsible for ensuring patients understand the results and for updating the medical records during the site training and in the Information Pack<br>• Provide clear and precise documentation of side effects during future courses of penicillin in the Information Pack
To provide easily accessible tools that are suitable for use by general practice clinicians	• Make interventions materials for clinicians accessible, short, easy to follow, easy to implement and not increase workload
To provide easily accessible resources for patients	• Make patients materials brief, easy to read, accessible<br>• Make materials easy to carry with them at all time and that provide evidence of the results of their test

For peer review only

Behavioural analysis

The aim of the behavioural analysis was to use behaviour change theory to describe the content of the AlABAMA intervention package and map the evidence from the rapid review, qualitative studies

For peer review only - http://bmjopen.bmj.com/site/about/guidelines.xhtml

and expert consultations. We used a model of behaviour known as COM-B (Capability, Opportunity, Motivation, Behaviour)[22]. According to this model, behaviour is a result of these three constructs. Changing behaviour requires overcoming any presenting barriers in each of these areas. The Behaviour Change Wheel (BCW)[23] offers a systematic way to develop interventions to change each of these constructs. A more specific description of elements which can be delivered within intervention components is provided in a taxonomy of 93 behaviour change techniques (Behaviour Change Technique Taxonomy, v1; BCTT v1)[24]."

Methods

The first step of the behavioural analysis process was to define the problem in behavioural terms which resulted in the identification of the fours specific target behaviours.

As a second step, we identified what needed to change using the Theoretical Domain Framework (TDF)[25] which includes 14 domains such as knowledge, skills, beliefs about capabilities. Each domain of the TDF relates to a COM-B component. This step allowed us to identify target behaviours, their barriers and facilitators, and how intervention components would support desired behaviour change based on evidence collated in stage 1.

Finally, the intervention components were mapped to the TDF framework and the BCW referring to the BCTv1[24]. This produced a list of TDF barriers to the target behaviours, target constructs (what needs to change for the behaviour to occur), intervention functions (ways an intervention can change behaviour) and behaviour change techniques used for each of the barriers/facilitators.

Results

The full behavioural analysis is presented in supplementary materials (S1). Firstly, we identified barriers and facilitators to referral of low risk patients to PAT and patient attendance to PAT. The analysis highlighted that both clinicians' and patients' knowledge and perceptions of penicillin

For peer review only - http://bmjopen.bmj.com/site/about/guidelines.xhtml

allergy and test procedures could be modified; information needed to be supported by scientific evidence for clinicians and patients to be reassured that the test was safe. We designed a resource for clinicians entitled “Penicillin Allergy Testing: Information for general practice” which contained information on penicillin allergy and PAAP procedures. As part of the AlABAMA trial, this will be supported by site training and working instructions which provide practical guidance on screening patients and referral to PAT (relevant BCTs for clinicians were ‘information about antecedents’ and ‘information about health consequences’).

For patients we developed two patient booklets, one to be provided prior to PAT and one following a negative test result. All patients, on entering the trial, will have a consultation with a GP to answer questions and address concerns about PAT. We developed a patient booklet entitled “Penicillin Allergy Testing: going for a test” which included information on PAAP procedures and PAT safety (relevant BCTs for patients were ‘information about health consequences’ and ‘feedback on outcomes of the behaviour’).

The barriers to the prescription and consumption of first-line penicillin following a negative test result were patient and clinician beliefs about the accuracy of PAT and whether taking penicillin was safe. Clinicians also needed reassurance that colleagues saw de-labelling as beneficial and resources to support them in changing incorrect penicillin allergy records. We developed a second patient booklet entitled “Penicillin Allergy Testing: a negative test result”, which contained information about which antibiotics patients could safely take in the future following a negative test result, a negative result intervention card and a result letter which confirmed the patient allergy status to penicillin (relevant BCTs were ‘social support’ and ‘restructuring of the social and physical environment’). As part of the trial, clinicians will receive working instructions, which contain guidance on how to change the patient allergy label in medical records, result letter which confirms the patient allergy status to penicillin, and an electronic-pop up, which includes a reminder of the

For peer review only - http://bmjopen.bmj.com/site/about/guidelines.xhtml

patient's new allergy status (relevant BCTs were 'feedback on outcomes of behaviour' and 'adding objects to the environment').

Logic modelling

The next step included the development of a logic model, which summarised the behavioural analysis, providing a diagrammatic representation of the hypothesised processes and causal pathways from the intervention components to the desired outcomes.[26-27]

Methods

The research team opted for a process oriented iterative logic model which was refined during the whole intervention development stage.

Results

The logic model (Figure 1) included four components:

Intervention components and techniques: Intervention components were organised based on the two target groups (clinicians and patients). Intervention techniques summarised the BCTs used as part of the intervention which were identified in the behavioural analysis.

Intervention processes: These were the psychological factors which explained the relationships between the intervention components and the outcome of the intervention. These processes were described in terms of TDF domains. Each intervention technique was hypothesised to mainly affect one of these processes. As part of the intervention, clinicians would receive information on penicillin allergy and implications of incorrect allergy records in order to increase their knowledge of PAT and allergies. In addition to this, providing medical and scientific evidence and current guidelines on how to perform PAT would change their professional identity related to their role in the referral process and act as reinforcement to increase their motivation to refer patients in the future. Regarding the processes affecting patient's attendance of PAT, providing evidence of penicillin as best treatment

For peer review only - http://bmjopen.bmj.com/site/about/guidelines.xhtml

and the safety of PAT would increase their beliefs of positive consequences of accurate records; informing them of common reactions during PAT and reassuring them of monitoring at the clinic and at home would increase their knowledge on PAT, decrease their negative emotions, such as anxiety, and act as positive reinforcement to attend the clinic.

Both patients and clinicians would receive evidence of the accuracy of PAT, and of safety of prescribing penicillin after a negative test result, in order to increase their belief about positive consequences of prescribing penicillin after a negative test result. Finally, providing a negative test result card to use with clinicians would affect patients' expectations of social influences regarding clinician's acceptance of the test result.

Purported mediators: These are the target behaviours of the intervention which directly affect the outcomes. In the logic model the assessment of potential incorrect penicillin records was operationalised as the referral of low risk patients to PAT and patient attendance at PAT. The introduction of the PAAP was hypothesized to affect the change of incorrect penicillin allergy records (clinician changing medical records and patient acceptance of change of penicillin allergy status) which would ultimately affect the consumption of penicillin.

Outcomes: The behavioural outcomes of the model were the prescription and consumption of first-line penicillin when indicated.

Stage 3: Optimising the intervention materials

The development of the intervention materials was part of an iterative process which was initially informed by the findings of the rapid review, qualitative interviews and expert consultations. Once the first drafts of the intervention materials were developed, a think aloud approach was used with clinicians and members of the PPI group for the aim of feedback on intervention materials. As this did not constitute a formal qualitative evaluation ethical approval was not required. This series of

For peer review only - http://bmjopen.bmj.com/site/about/guidelines.xhtml

For peer review only

think-aloud consultations with clinicians and PPI representatives were conducted in order to gain their insights on the content, usability and format of the clinician "Penicillin Allergy Testing: Information for general practice" leaflet and two patient booklets ("Penicillin Allergy Testing: going for a test", "Penicillin Allergy testing: a negative test result"). Subsequently to the feedback received by the participants in the think-aloud consultations and discussions in further expert consultations, the intervention materials were optimised. Verbal consent was obtained from all participants, who agreed to take part in the consultations and for notes to be taken. Consultations were not recorded and interviews were only used to refine intervention materials.

Think-aloud consultations with GPs

Methods

Think aloud telephone consultations were conducted with 6 participants by MW; 2 additional participants provided feedback via email. Four of the participants had previously taken part in the qualitative study on GP views on the barriers and facilitators for PAT and antibiotic use after a negative test (mentioned in the section Qualitative methods above Ref); four additional participants were recruited using convenience sampling. Consultations were not recorded, but the interviewer took detailed notes. Consultations focused on GP views of each section of the "Penicillin Allergy Testing: Information for general practice" (supplementary materials S2).

Results

The leaflet was well received. Participants reported that it was informative, useful and generally easy to read. The participants perceived it not only as information for themselves, but also a tool to use in a consultation with patients. Some participants felt that they knew about the consequences of incorrect penicillin allergy record; and therefore the leaflet could be shortened. Most participants understood the testing stages; however, a couple of participants were confused about which stages

For peer review only - http://bmjopen.bmj.com/site/about/guidelines.xhtml

of the test patients could skip. One participant wanted exact doses of penicillin specific (rather than just amounts). Regarding the section on patient discussions, some clinicians felt that there was no need to discuss the test with patients. Participants queried whether being tested with amoxicillin meant that the patient could now take all penicillin based antibiotics and wanted more information.

Clinicians' feedback was collated and organised in a 'table of changes' (supplementary materials S3) where suggested changes were listed and given a level of priority for that change, following the MoScoW framework [28], and the source of the suggested change (expert opinion, research team, clinical research team, literature review). Changes to the "Penicillin Allergy Testing: Information for general practice" leaflet included changes to the title, to the exact doses of penicillin given to the patients during the test, information about side effects and information about which antibiotics patients with a negative test result can take safely.

Think-aloud consultations with patients

Methods

Think aloud consultations were conducted with 7 patients (3 with experience of PAT and 4 with no experience) by MW. Five of these patients had previously taken part in the qualitative study on views on the barriers and facilitators for PAT and antibiotic use after a negative test (mentioned in the section Qualitative methods). These participants were asked at the end of the initial interview whether they would like to comment on the intervention materials at a later date; the additional two participants were identified using convenience sampling. Again, consultations were not recorded, but the interviewer took detailed notes. Consultations asked their views about the two patient booklets ("Penicillin Allergy Testing: going for a test", "Penicillin Allergy testing: a negative test result") and the intervention card (supplementary materials S2).

For peer review only - http://bmjopen.bmj.com/site/about/guidelines.xhtml

Results

The booklets and intervention card were very well received by the participants. Participants considered the booklets to have the right amount of information and felt they were generally easy to read. Patients reported that the booklets convinced them that going for a PAT could be beneficial. They felt that they could relate to the description of how people were given penicillin allergy labels. Patients thought the description of the test was clear and they knew what to expect. Statistics about the prevalence of allergy were not always understood by the participants, as the participants often thought that 1 in 10 people are allergic and they wanted a more visual presentation of this key information. Participants were unsure what narrow and broad spectrum antibiotics were and did not recognise MRSA abbreviation. Participants did not always know that penicillin is more than one antibiotic. The participants wanted to have a separate paragraph on what could happen during the test and what could happen during three days of taking penicillin at home. They also wanted reassurance that three days would be enough to detect delayed reactions. The participants wanted more reassurance that after being tested with one type of penicillin (e.g. amoxicillin), it would mean that they could safely take all penicillin antibiotics. The participants were slightly concerned about the risk of allergic reaction in the future (despite negative test results).

Patient feedback was collated in a table of changes. Changes made to the booklets were the selection of new images of patients for the front cover, inclusion of definitions of narrow and broad spectrum antibiotics, and reassurance that 3 days of oral challenge would be enough to detect delayed reactions to penicillin.

Intervention components

The development and optimisation of clinician and patient intervention materials was part of an iterative process . Figures 2 and 3 present the example of this process for the development of one

For peer review only - http://bmjopen.bmj.com/site/about/guidelines.xhtml

section of the “Penicillin allergy testing: going for a test” patient booklet and the “Information for General Practice” leaflet for clinicians.

All Working Instructions developed to support clinicians and research nurses activities as part of the AlABAMA intervention package were shown to a group of clinicians to gain their feedback on content and layout. Among the clinicians who provided feedback there were two practice managers, and one nurse. Their overall feedback was positive and the main changes to the intervention materials included the identification of the best way of updating the patient’s medical records after PAT, and the introduction of screenshots of the medical record in the working instructions.

All participants letter (patient appointment letter, patient result letter, clinician result letter) were developed among the wider clinical and research team in order to make them effective in motivating patient to attend the penicillin allergy testing and in order to persuade clinician to change patient records and prescribe penicillin after a negative test result, and patient to consume penicillin after a negative test result.

At the end of the intervention development stage, a description of the intervention was completed following the TIDieR [29] guidance (supplementary materials S4) together with a description of the intervention components for clinicians and patients (Table 2). We have followed the GUIDED[30] checklist (supplementary materials S5), when reporting intervention development in this paper.

Table 2: Overview of the key intervention components of the AlABAMA intervention package.

PAT = Penicillin Allergy Testing

Intervention components	Description
**For clinicians**	

For peer review only

"Penicillin Allergy Testing: Information for general practice" leaflet	Information leaflet which includes evidence-based information to increase knowledge about penicillin allergy testing and motivation to refer patients to PAT and prescribe penicillin after a negative PAT result
Electronic health record pop-up	Electronic pop-up on medical records which tells clinicians that patient has had a negative test result and that their allergy status needs to be amended
Allergy result letter	A letter is sent to the GP to inform them of the patient's penicillin allergy test result, including details on which test(s) they undertook, whether it is safe or not to prescribe penicillin in the future and instructions on how to change the allergy label
Consultation with patients	Discussions with patients to check eligibility for the trial and to answer any queries about the trial and PAAP testing
Site Training and working instructions	Training in trial procedures delivered to GP leads including provision of information on penicillin allergy de-labelling, the referral process, the three stages of the PAAP and the interpretation of test results
**For patients in intervention arm**	
"Penicillin allergy testing: going for a test" booklet	A booklet to inform patients, in the PAAP intervention arm, about incorrect allergy records, how they may benefit from having a penicillin allergy test and what the test involves
Penicillin Allergy Test appointment letter	The letter includes information on the PAAP procedures in hospital, including pre-test assessment and monitoring during the test and at home
Penicillin Allergy Assessment Pathway (PAAP)	Appointment at the immunology clinic for patients in the PAAP intervention arm. At the appointment patients will complete Stage 1, Stage 2 and/or Stage 3 of the PAAP pathway
Allergy test result letter	Patient will receive a letter with information on the result of their allergy test and whether it is safe or not to take penicillin in the future
"Penicillin allergy testing: a negative	A booklet on the reliability of the test results and consequences of a

For peer review only - http://bmjopen.bmj.com/site/about/guidelines.xhtml

test result" booklet	negative test result (sent with allergy test result letter).
Post-test intervention card	Laminated credit card-sized card which says which test the patient has had and confirms the negative allergy result
For all patients in control and intervention arm	
An invitation letter and Participant information Sheet	An invitation letter and Participant information Sheet on the purpose of the trial and what the research study would involve for patient participants
Discussion with clinicians	Discussion with clinicians about attending the test to ask any queries around the benefits of taking the test and why removing the incorrect record might be good

DISCUSSION

This paper describes the development of the AlABAMA intervention package, which aimed to change behaviours that facilitate penicillin allergy testing and subsequently amend incorrect allergy records in English general practice. Such changes in clinician and patient behaviours have potential to significantly impact on antibiotic prescribing and consumption. The approach used here has previously been used for the development of behaviour change interventions targeted to reduce antibiotic prescribing by clinicians in primary[31] and secondary care[32] but it is the first time that this approach has been used to develop an intervention to amend incorrect penicillin allergy records. The transparency of the intervention development process will inform intervention developers on how this methodology could be used in different contexts, and will facilitate the comparison with other interventions which have used similar processes.

The evidence collated from the rapid review and qualitative interviews allowed in-depth understanding of participant needs (lack of knowledge of PAT, lack of knowledge of negative consequences of allergy labels) and which behavioural influences needed to be modified as part of the intervention. The mapping of these behavioural influences and the AlABAMA intervention

For peer review only - http://bmjopen.bmj.com/site/about/guidelines.xhtml

package components to behaviour change theory and the logic model allowed a transparent reporting of the psychological processes that are hypothesized to explain the effect of the intervention components on the outcomes. More specifically it highlighted the BCTs used in each component of the AlABAMA intervention package and how they addressed the barriers identified in the rapid review and qualitative work. It also explained which psychological mechanisms were hypothesised to change by the intervention components (clinicians' knowledge on penicillin allergy and PAT procedures, patients' beliefs about positive consequences of taking the PAT) and which target behaviours (referral and attendance to PAT, prescription and consumption of penicillin first line) would likely affect the intervention outcomes. Think-aloud interviews with patients and clinicians, and in-depth feedback from the clinical research team on the intervention materials, highlighted changes that needed to be made in order to increase their acceptability and potential effectiveness.

The AlABAMA intervention package targeted clinician referral of patients for PAT and updating incorrect penicillin allergy records; factors previously identified in previous qualitative research as barriers to effective penicillin allergy de-labelling.[11-20] Recent exploration of clinician reported barriers and enablers to identifying and de-labelling hospital inpatients with incorrect penicillin allergy records has highlighted the need to introduce patient education concerning the risks of avoiding penicillin.[24] Inconsistencies in the management of penicillin allergic patients were reported, together with a lack of time to discuss allergy testing, and the need to improve communication between primary and secondary care about patient allergy status, as well as updating of patient medical records.[33] A previous exploration of views about implementing de-labelling of patients ahead of elective surgery identified barriers to implementing it on a large scale, such as human factors linked to anxiety and financial implications. These human factors were: lack of interest from patients in undertaking an allergy test; lack of acceptance of the test result among clinicians; high proportion of patient re-labelled themselves after a negative testing for penicillin allergy or re-

For peer review only - http://bmjopen.bmj.com/site/about/guidelines.xhtml

labelling by health care professionals. The financial barrier was significant despite long-term cost benefit, as there is an upfront cost to perform the test.[34]

A limitation of the AlABAMA intervention package is to how widely applicable it might be. Allergy services across the UK vary significantly, and access to specialist testing ranges widely. The AlABAMA programme will focus on one geographical area (the North of England), which will be covered by the specialist allergy unit participating in the AlABAMA trial. The intervention will be centred around functionality to be incorporated into SystemOne (an electronic medical record system widely used in general practice) but the intervention package is not necessarily suitable for use in other areas of England, the UK or wider. The contextual factors to delivery should be considered. Moreover, it is only a small group of patients (around 25-30%) who are suitable to undergo the abbreviated test (patient history, skin test and oral challenge test). Many will still require full assessment by an immunologist or allergist as per current guidelines. Cost-effectiveness analysis of the PAT and intervention package as a whole will be carried out the in upcoming AlABAMA trial.

Conclusions

Current clinical practice involves referral to penicillin allergy testing with little attention to other elements of the pathway that help to ensure that testing impacts positively on patient care. This study presents the development of a behavioural intervention package to support the process of amending incorrect penicillin allergy records. Numerous barriers to the uptake of PAT have been identified as well as penicillin prescribing and consumption following a negative test. We have identified relevant behaviour change techniques to inform the development of the AlABAMA intervention package to overcome these barriers. The intervention is currently being tested in a feasibility trial in primary care to lead on to a randomised-controlled trial.

Acknowledgements: The AlABAMA team consists of: Jenny Boards, Emily Bongard, Christopher C Butler, Mina Davoudianfar, Mandy East, Joanne Fielding, Philip Howard, Sue H Pavitt, Catherine E

For peer review only - http://bmjopen.bmj.com/site/about/guidelines.xhtml

Porter, Jonathan AT Sandoe, Marta Santillo, Louise Savic, Sinisa Savic, Bethany Shinkins, Sarah Tonkin-Crine, Marta Wanat, Robert West, Ly-Mee Yu.

Author contributions: JS, SP, CB, SS, LS, EB and STC designed and developed the AlABAMA programme and the plan for the intervention. MW and STC led the intervention development, with input from other co-authors. MW conducted the rapid review and qualitative work. MS led the behavioural analysis. CB, EB, MD, CP and JF led the design of the trial and trial materials. MS drafted the manuscript. All authors critically reviewed the manuscript and approved the final version.

Funding: This study summarises independent research funded by the National Institute for Health Research (NIHR) under its Programme Grants for Applied Research Programme (Grant Reference Number RP-PG-1214-20007). STC received additional funding from the National Institute for Health Research Health Protection Research Unit (NIHR HPRU) in Healthcare Associated Infections and Antimicrobial Resistance at the University of Oxford in partnership with Public Health England (PHE) [HPRU-2012-10041]. The research is supported by the National Institute for Health Research (NIHR) infrastructure at Leeds. The funder had no role in the design of the study; in the collection, analyses, or interpretation of data; in the writing of the manuscript, or in the decision to publish.

Disclaimer: The views expressed are those from the authors and not necessary those of the NHS, NIHR or the Department of Health and Social Care.

Competing Interests: The authors have received funding from the National Institute for Health Research.

Patient Consent: Not required

For peer review only

For peer review only - http://bmjopen.bmj.com/site/about/guidelines.xhtml

Ethics approval: This study received ethical approval from the London Bridge Research Ethics Committee (Ref: 19/LO/0176).

Data sharing statements: No additional data available

REFERENCES

1. McLean-Tooke A, Aldridge C, Stroud C, et al. Practical management of antibiotic allergy in adults. *Journal of Clinical Pathology* 2011;64(3):192-99. doi: 10.1136/jcp.2010.077289

2. Borch JE, Andersen KE, Bindslev-Jensen C. The prevalence of suspected and challenge-verified penicillin allergy in a university hospital population. *Basic Clin Pharmacol Toxicol* 2006;98(4):357-62. doi: 10.1111/j.1742-7843.2006.pto_230.x

3. Reddy V, Baman NS, Whitener C, et al. Drug Resistant Infections with Methicillin-Resistant Staphylococcus Aureus, Clostridium Difficile, and Vancomycin Resistant Enterococcus Are Associated with a Higher Prevalence of Penicillin Allergy. *J Allergy Clin Immunol* 2013;131(2):AB170-AB70. doi: 10.1016/j.jaci.2012.12.1269

4. West RM, Smith CJ, Pavitt SH, et al. "Warning: allergic to penicillin': association between penicillin allergy status in 2.3 million NHS general practice electronic health records, antibiotic prescribing and health outcomes. *J Antimicrob Chemother* 2019;74(7):2075-82. doi: 10.1093/jac/dkz127

5. Krishna MT, Huissoon AP, Li M, et al. Enhancing antibiotic stewardship by tackling "spurious" penicillin allergy. *Clin Exp Allergy* 2017;47(11):1362-73. doi: 10.1111/cea.13044

For peer review only - http://bmjopen.bmj.com/site/about/guidelines.xhtml

6. NICE. Drug allergy: giagnosis and management of drug allergy in adults, children and young poeple. NICE clinical guideline 183. 2014

7. Yardley L, Morrison L, Bradbury K, et al. The Person-Based Approach to Intervention Development: Application to Digital Health-Related Behavior Change Interventions. *Journal of Medical Internet Research* 2015;17(1) doi: 10.2196/jmir.4055

8. Greenwell K, Sivyer K, Vedhara K, et al. Intervention planning for the REDUCE maintenance intervention: a digital intervention to reduce reulceration risk among patients with a history of diabetic foot ulcers. *BMJ Open* 2018;8(5):12. doi: 10.1136/bmjopen-2017-019865

9. Band R, Bradbury K, Morton K, et al. Intervention planning for a digital intervention for self-management of hypertension: a theory-, evidence- and person- based approach. *Implementation Science* 2017;12 doi: 10.1186/s13012-017-0553-4

10. Little P, Stuart B, Francis N, et al. Effects of internet-based training on antibiotic prescribing rates for acute respiratory-tract infections: a multinational, cluster, randomised, factorial, controlled trial. *The Lancet* 2013;382(9899):1175-82.

11. Wanat M, Anthierens S, Butler CC, et al. Patient and Prescriber Views of Penicillin Allergy Testing and Subsequent Antibiotic Use: A Rapid Review. *Antibiotics-Basel* 2018;7(3) doi: 10.3390/antibiotics7030071

12. Khangura S, Polisena J, Clifford TJ, et al. RAPID REVIEW: AN EMERGING APPROACH TO EVIDENCE SYNTHESIS IN HEALTH TECHNOLOGY ASSESSMENT. *International Journal of Technology Assessment in Health Care* 2014;30(1):20-27. doi: 10.1017/s0266462313000664

For peer review only - http://bmjopen.bmj.com/site/about/guidelines.xhtml

13. Sundquist, B.K.; Bowen, B.J.; Otabor, U.; Celestin, J.; Sorum, P.C. Proactive penicillin allergy testing in primary care patients labeled as allergic: Outcomes and barriers. *Postgrad. Med.* **2017**

14. Jose J, Ishmael FTJJoA, Immunology C. A drug allergy education handout is an easy and effective method to improve patient awareness of penicillin allergy and increase penicillin testing. 2017;139(2):AB29.

15. Elkhalifa, S, Calisti, G, Owens, L, Garcez, T, Dodgson, K, Alexander, K. Management of patients with a history of penicillin allergy: Barriers to best practice and strategies to overcome them. *Allergy* 2017, *72*, 224.

16. Hayoun, MB, Bourrier, T, Pognonec, C, Sanfiorenzo, C, Marquette, C, Leroy, S. The impact of allergy to beta-lactam antibiotocs on general practitioners and patients in a cohort of154 French patients. *Rev. Fr. Allergol.* 2015, *55*, 333–340.

17. Picard, M, Bégin, P, Bouchard, H, Cloutier, J, Lacombe-Barrios, J, Paradis, J, Des Roches, A, Laufer, B, Paradis, L. Treatment of patients with a history of penicillin allergy in a large tertiary-care academic hospital. *J. Allergy Clin. Immunol. Pract.* 2013, *1*, 252–257.

18. Soni, D, Ramsey, A, Staicu, M. A clinical perspective: The prescriber's true understanding of the 'penicillin allergic'patient. *Ann. Allergy Asthma Immunol.* 2016, 117, S18

19. Trubiano, JA, Beekmann, SE, Worth, LJ, Polgreen, PM, Thursky, KA, Slavin, MA, Grayson, M.L, Phillips, E.J. Improving Antimicrobial Stewardship by Antibiotic Allergy Delabeling: Evaluation of Knowledge, Attitude, and Practices Throughout the Emerging Infections Network. *Open Forum Infect. Dis.* 2016, *3*

For peer review only - http://bmjopen.bmj.com/site/about/guidelines.xhtml

20. Wanat M, Anthierens S, Butler CC, et al. Patient and Primary Care Physician Perceptions of Penicillin Allergy Testing and Subsequent Use of Penicillin-Containing Antibiotics: A Qualitative Study. *Journal of Allergy and Clinical Immunology-in Practice* 2019;7(6):1888-+. doi: 10.1016/j.jaip.2019.02.036

21. Braun V, Clarke V. What can “thematic analysis” offer health and wellbeing researchers?. *International journal of qualitative studies on health and well-being.* 2014;9.

22. Michie S, Atkins L, West R. The behaviour change wheel: a guide to designing interventions. Needed: physician leaders. 2014;26:146.

23. Michie S, van Stralen MM, West R. The behaviour change wheel: A new method for characterising and designing behaviour change interventions. *Implementation Science* 2011;6 doi: 10.1186/1748-5908-6-42

24. Michie S, Richardson M, Johnston M, et al. The Behavior Change Technique Taxonomy (v1) of 93 Hierarchically Clustered Techniques: Building an International Consensus for the Reporting of Behavior Change Interventions. *Annals of Behavioral Medicine* 2013;46(1):81-95. doi: 10.1007/s12160-013-9486-6

25. Atkins L, Francis J, Islam R, et al. A guide to using the Theoretical Domains Framework of behaviour change to investigate implementation problems. *Implementation Science* 2017;12 doi: 10.1186/s13012-017-0605-9

26. Baxter S, Killoran A, Kelly MP, et al. Synthesizing diverse evidence: the use of primary qualitative data analysis methods and logic models in public health reviews. *Public Health* 2010;124(2):99-106. doi: 10.1016/j.puhe.2010.01.002

27. Baxter SK, Blank L, Woods HB, et al. Using logic model methods in systematic review synthesis: describing complex pathways in referral management interventions. *Bmc Medical Research Methodology* 2014;14 doi: 10.1186/1471-2288-14-62

For peer review only

For peer review only - http://bmjopen.bmj.com/site/about/guidelines.xhtml

28. Bradbury K, Morton K, Band R, et al. Using the Person-Based Approach to optimise a digital intervention for the management of hypertension. *PloS one* 2018;13(5):e0196868.

29. Hoffmann TC, Glasziou PP, Boutron I, et al. Better reporting of interventions: template for intervention description and replication (TIDieR) checklist and guide. *Bmj-British Medical Journal* 2014;348 doi: 10.1136/bmj.g1687

30. Duncan E, O'Cathain A, Rousseau N, Croot L, Sworn K, Turner KM, Yardley L, Hoddinott P. Guidance for reporting intervention development studies in health research (GUIDED): an evidence-based consensus study. BMJ open. 2020 Apr 1;10(4):e033516.

31. Anthierens S, Tonkin-Crine S, Douglas E, et al. General practitioners' views on the acceptability and applicability of a web-based intervention to reduce antibiotic prescribing for acute cough in multiple European countries: a qualitative study prior to a randomised trial. *BMC family practice* 2012;13(1):101.

32. Santillo M, Sivyer K, Krusche A, et al. Intervention planning for Antibiotic Review Kit (ARK): a digital and behavioural intervention to safely review and reduce antibiotic prescriptions in acute and general medicine. *J Antimicrob Chemother* 2019

33. Powell N, Wilcock M, Roberts N, et al. Focus group study exploring the issues and the solutions to incorrect penicillin allergy-labelled patients: an antibiotic stewardship patient safety initiative. *European Journal of Hospital Pharmacy* 2019:ejhpharm-2019-001863.

34. Savic L, Gurr L, Kaura V, et al. Penicillin allergy de-labelling ahead of elective surgery: feasibility and barriers. *Br J Anaesth* 2019;123(1):E110-E16. doi: 10.1016/j.bja.2018.09.009

For peer review only - http://bmjopen.bmj.com/site/about/guidelines.xhtml

For peer review only

For peer review only - http://bmjopen.bmj.com/site/about/guidelines.xhtml

Figure 1: AlABAMA logic model

Figure 2: Example of intervention development for clinician materials

Figure 3: Example of intervention development for patient materials. PAT = Penicillin Allergy Testing

For peer review only

For peer review only - http://bmjopen.bmj.com/site/about/guidelines.xhtml

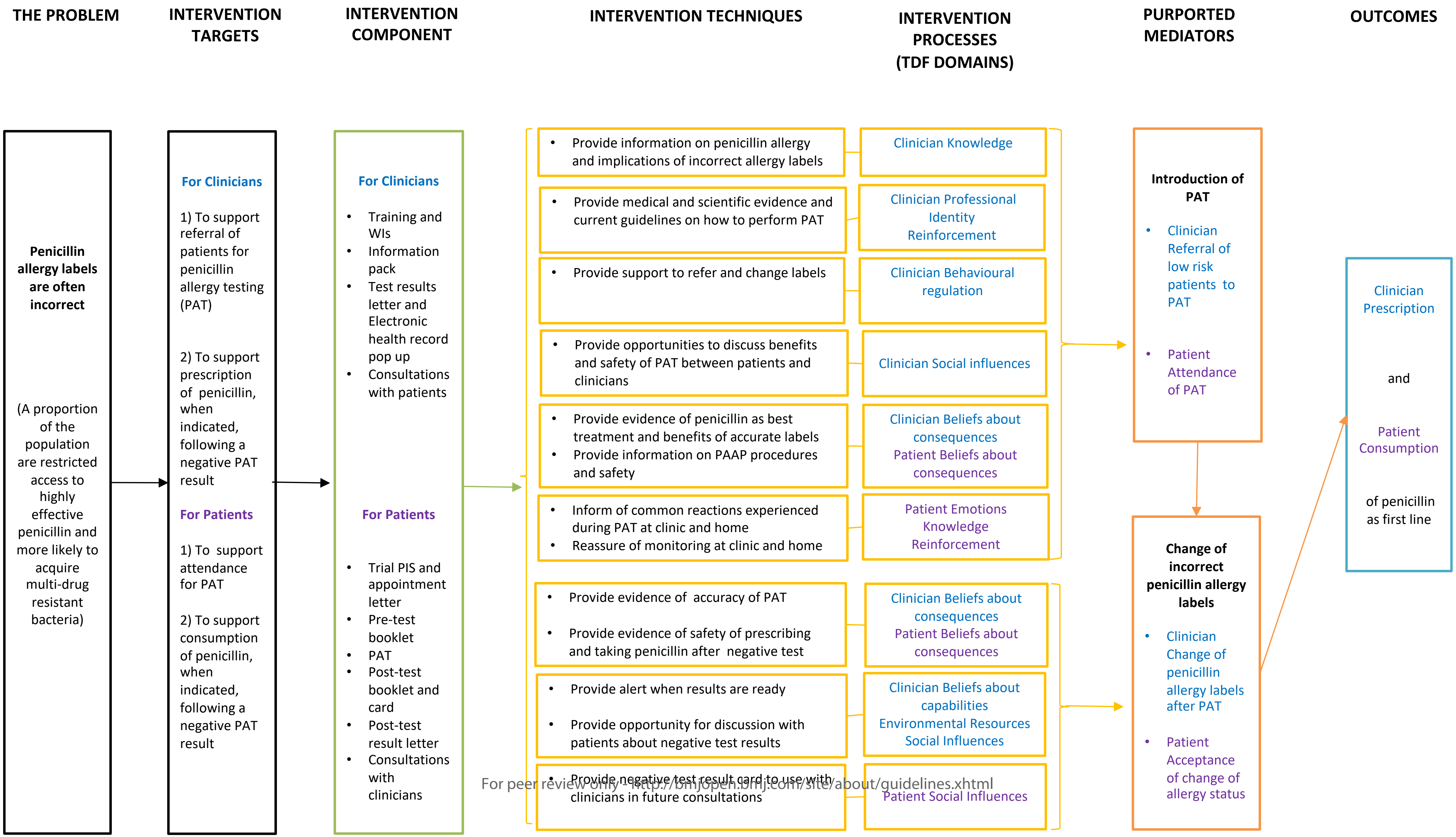
THE PROBLEM
INTERVENTION TARGETS
INTERVENTION COMPONENT
INTERVENTION TECHNIQUES
INTERVENTION PROCESSES (TDF DOMAINS)
PURPORTED MEDIATORS
OUTCOMES
Penicillin allergy labels are often incorrect
(A proportion of the population are restricted access to highly effective penicillin and more likely to acquire multi-drug resistant bacteria)
For Clinicians
1) To support referral of patients for penicillin allergy testing (PAT)
2) To support prescription of penicillin, when indicated, following a negative PAT result
For Patients
1) To support attendance for PAT
2) To support consumption of penicillin, when indicated, following a negative PAT result
For Clinicians
• Training and WIs
• Information pack
• Test results letter and Electronic health record pop up
• Consultations with patients
For Patients
• Trial PIS and appointment letter
• Pre-test booklet
• PAT
• Post-test booklet and card
• Post-test result letter
• Consultations with clinicians
• Provide information on penicillin allergy and implications of incorrect allergy labels
• Provide medical and scientific evidence and current guidelines on how to perform PAT
• Provide support to refer and change labels
• Provide opportunities to discuss benefits and safety of PAT between patients and clinicians
• Provide evidence of penicillin as best treatment and benefits of accurate labels
• Provide information on PAAP procedures and safety
• Inform of common reactions experienced during PAT at clinic and home
• Reassure of monitoring at clinic and home
• Provide evidence of accuracy of PAT
• Provide evidence of safety of prescribing and taking penicillin after negative test
• Provide alert when results are ready
• Provide opportunity for discussion with patients about negative test results
• Provide negative test result card to use with clinicians in future consultations
Clinician Knowledge
Clinician Professional Identity Reinforcement
Clinician Behavioural regulation
Clinician Social influences
Clinician Beliefs about consequences
Patient Beliefs about consequences
Patient Emotions
Knowledge
Reinforcement
Clinician Beliefs about consequences
Patient Beliefs about consequences
Clinician Beliefs about capabilities
Environmental Resources
Social Influences
Patient Social Influences
Introduction of PAT
• Clinician Referral of low risk patients to PAT
• Patient Attendance of PAT
Change of incorrect penicillin allergy labels
• Clinician Change of penicillin allergy labels after PAT
• Patient Acceptance of change of allergy status
Clinician Prescription
and
Patient Consumption
of penicillin as first line

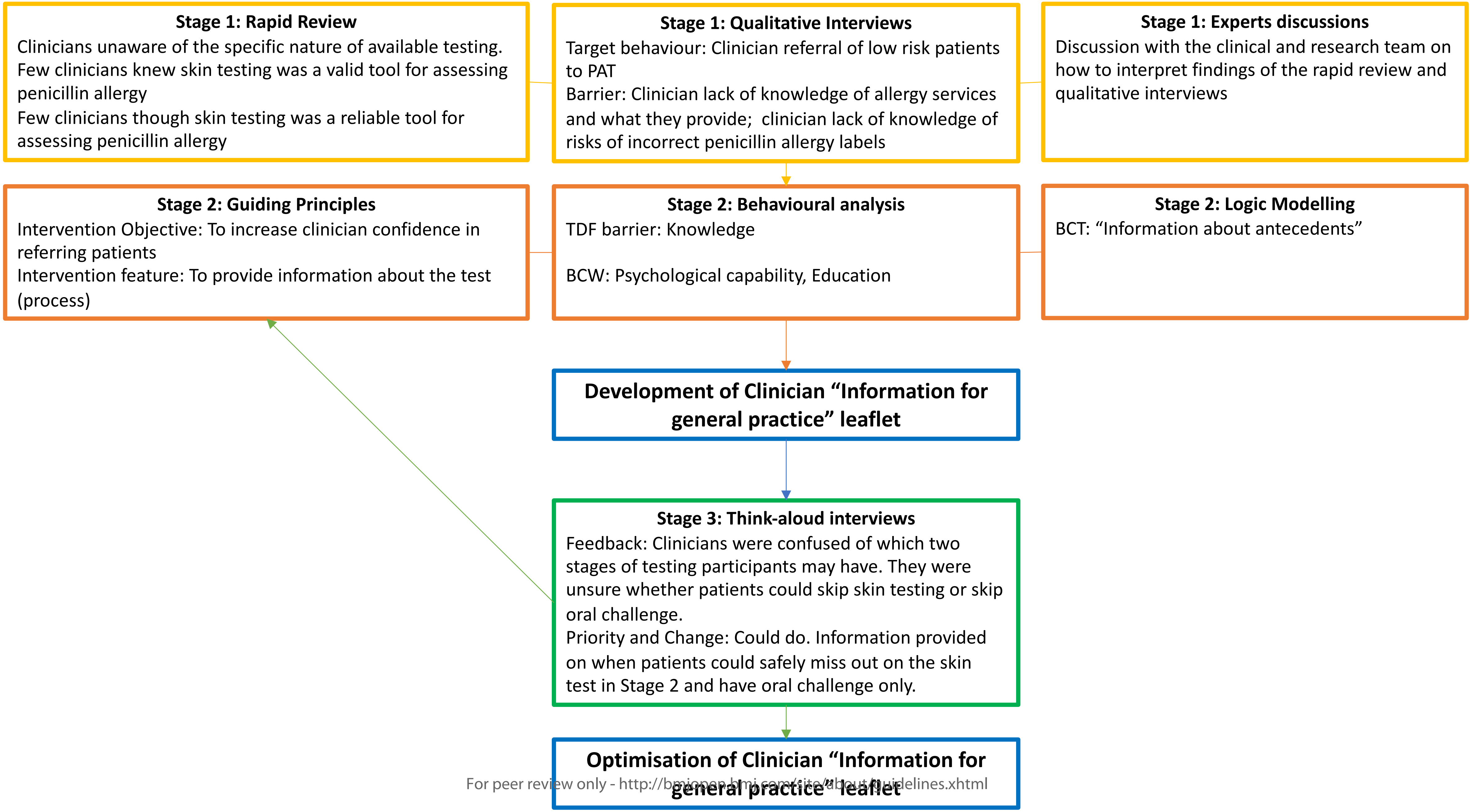

For peer review only - http://bmjopen.bmj.com/site/about/guidelines.xhtml

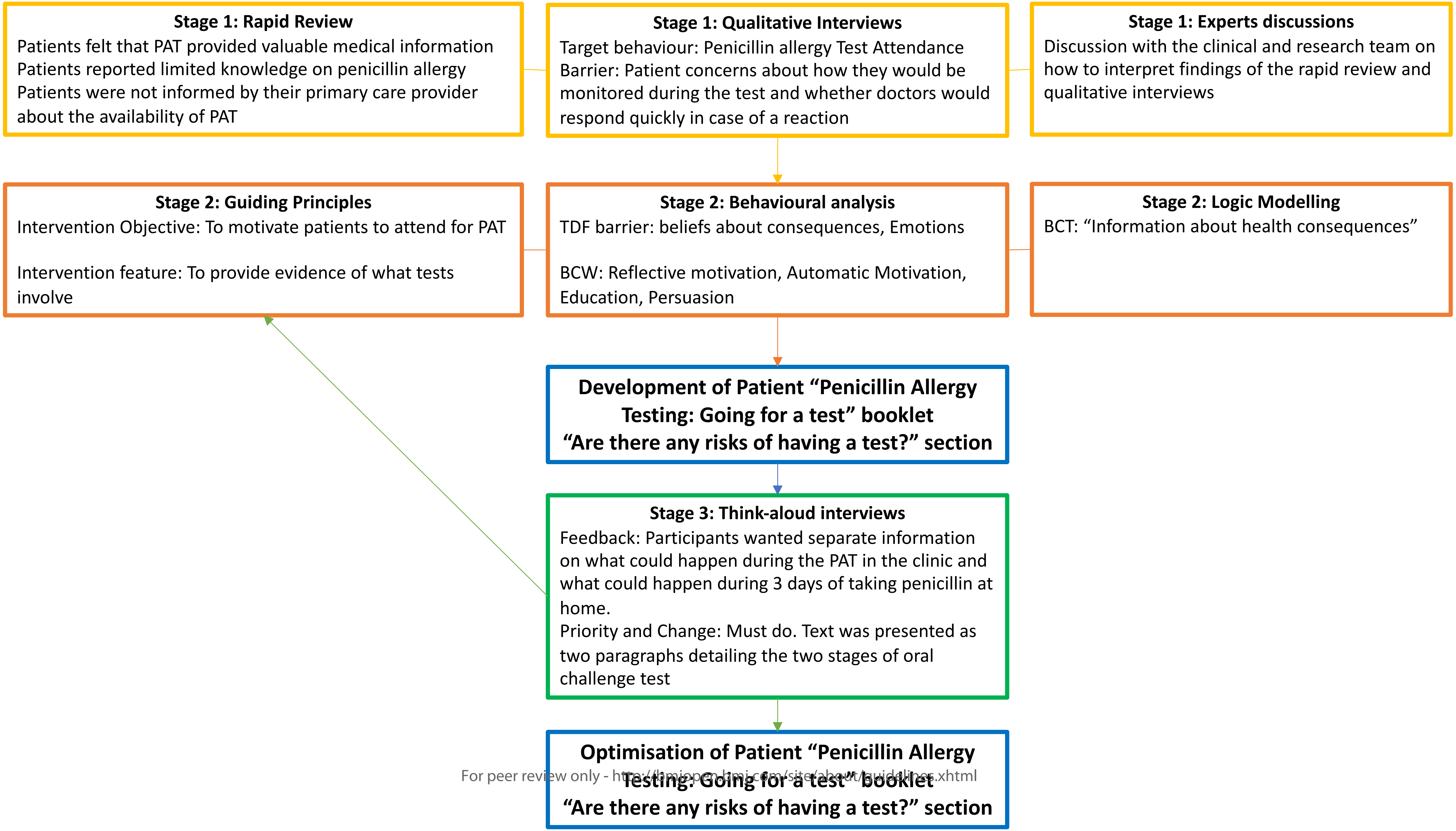
Stage 1: Rapid Review
Patients felt that PAT provided valuable medical information
Patients reported limited knowledge on penicillin allergy
Patients were not informed by their primary care provider about the availability of PAT
Stage 1: Qualitative Interviews
Target behaviour: Penicillin allergy Test Attendance
Barrier: Patient concerns about how they would be monitored during the test and whether doctors would respond quickly in case of a reaction
Stage 1: Experts discussions
Discussion with the clinical and research team on how to interpret findings of the rapid review and qualitative interviews
Stage 2: Guiding Principles
Intervention Objective: To motivate patients to attend for PAT
Intervention feature: To provide evidence of what tests involve
Stage 2: Behavioural analysis
TDF barrier: beliefs about consequences, Emotions
BCW: Reflective motivation, Automatic Motivation, Education, Persuasion
Stage 2: Logic Modelling
BCT: “Information about health consequences”
Development of Patient “Penicillin Allergy Testing: Going for a test” booklet
“Are there any risks of having a test?” section
Stage 3: Think-aloud interviews
Feedback: Participants wanted separate information on what could happen during the PAT in the clinic and what could happen during 3 days of taking penicillin at home.
Priority and Change: Must do. Text was presented as two paragraphs detailing the two stages of oral challenge test
Optimisation of Patient “Penicillin Allergy Testing: Going for a test” booklet
“Are there any risks of having a test?” section

SUPPLEMETARY MATERIALS

S1. Behavioural Analysis Table AlABAMA

S2. Think-aloud topic guides for clinicians and patients

S3. Table of changes: "Penicillin Allergy Testing: going for a test" booklet

S4. Full description of the AlABAMA (ALlergy AntiBiotics And Microbial resistAnce) Intervention package in accordance with the Template for Intervention Description and Replication (TIDieR) framework

S5. GUIDED – a guideline for reporting for intervention development studies.

S1. Behavioural Analysis Table

Target Behaviours:

- Clinician referral of low risk patients to undertake the Penicillin Allergy Testing (PAT)
- Clinician prescription of penicillin after negative test result
- Patient attendance at Penicillin Allergy Testing (PAT)
- Patient consumption of penicillin when prescribed after negative test result

Notes: [QI]: Qualitative Interviews; [RR]: Rapid Review; [LR]: Literature Review; PIS; Participant Information Sheet

Table S1a Behavioural Analysis Table for the AlABAMA intervention, clinician behaviours

For peer review only

**Barriers / *facilitators* to target behaviours**	**Intervention Component/s**	**Intervention ingredient**	**Theoretical Domains Framework (TDF)**	**Target construct (BCW)**	**Intervention function (BCW)**	**Behaviour Change Technique (using 93 BCT taxonomy v1)**
**Target behaviour: Clinician referral of low risks patients to Penicillin Allergy Testing**						
**Lack of knowledge** about incidence of **incorrect penicillin allergy labels** [QI]	"Penicillin Allergy Testing: Information for general practice" leaflet<br><br>Training	• Provide information on penicillin allergy records and its implications	Knowledge	Psychological capability	Education	4.2 information about antecedents
**Lack of knowledge** of how **accurate** the tests are [QI]	"Penicillin Allergy Testing: Information for general practice" leaflet	• Provide information that the oral challenge test is the gold standard test for penicillin allergy	Knowledge	Psychological capability	Education	4.2 information about antecedents
**Lack of knowledge** of the existence of **allergy services** or what they involve [QI]	"Penicillin Allergy Testing: Information for general practice" leaflet<br><br>Training	• Provide information what the penicillin allergy test involves and where it will take place	Knowledge	Psychological capability	Education	4.2 information about antecedents
**Poor experience** of PAT [QI, RR]	"Penicillin Allergy Testing: Information for general practice" leaflet	• Provide information about why it is important to have correct penicillin allergy labels and what the test involve	Skills<br><br>Behavioural regulation	Physical capability<br><br>Psychological Capability	Training<br><br>Education<br><br>Persuasion	2.7 feedback on outcome of behaviour
**Lack of understanding** PAT procedures and results [QI, RR]	"Penicillin Allergy Testing: Information for general practice" leaflet<br><br>Training	• Provide information about the three stages of penicillin allergy testing and what negative and positive results mean	Knowledge	Psychological capability	Education	4.2 information about antecedents

For peer review only

**Lack of guidelines** in learning about **benefits and risks** of allergy services [QI, RR]	"Penicillin Allergy Testing: Information for general practice" leaflet	• Provide a series of references around penicillin allergy and PAT, including NICE guidelines	Knowledge	Psychological capability	Education	9.1 credible source
**Lack of guidance on referral** [QI, RR]	"Penicillin Allergy Testing: Information for general practice" leaflet<br><br>Training and WIs	• Provide information about the NICE guidelines<br>• Provide a system to refer low risks patients to do testing	Knowledge<br><br>Skills	Psychological capability<br><br>Physical skills	Education<br><br>Training	1.4 action planning<br><br>4.1 instructions on how to perform a behaviour
**Lack of unified approach** for referral of patients to PAT [RR]	"Penicillin Allergy Testing: Information for general practice" leaflet<br><br>Training and WIs	• Provide references on NICE guidelines on referral<br>• Provide a system to refer low risks patients to do testing	Skills<br><br>Social influences	Physical skills<br><br>Social opportunity	Training<br><br>Modelling	1.4 action planning<br><br>4.1 instructions on how to perform a behaviour
**Lack of knowledge** regarding the **oral challenge test** [QI]	"Penicillin Allergy Testing: Information for general practice" leaflet<br><br>Training	• Provide information about the oral challenge test (stage 3 or PAT), why it is done, what the procedure is and how accurate it is	Knowledge	Psychological capability	Education	4.2 information about antecedents
Perception of **overburdening the allergy service** [QI]	"Penicillin Allergy Testing: Information for general practice" leaflet	• Provide information around NICE guidelines in support of PAT	Social/Professional role and identity<br><br>Knowledge	Social opportunity<br><br>Psychological capability	Modelling<br><br>Enablement<br><br>Training	9.1 credible source<br><br>6.3 information about others' approval

For peer review only

**Lack of follow up** after referral [QI]	Pop-up alert<br><br>"Penicillin Allergy Testing: Information for general practice" leaflet<br><br>Training	• Provide information that they will receive confirmation about the patients' results	Environmental Context and resources	Physical opportunity	Environmental restructuring<br><br>Enablement	7.1 prompts/cues<br><br>2.7 feedback on outcome of behaviour
Expectations that **patients may not want to be tested** [RR]	Consultations with patients<br><br>"Penicillin allergy testing: going for a test" booklet	• Provide pre-test booklet to discuss with patients<br>• Provide the opportunity to discuss the benefits of the test during the consultation	Social influences	Social opportunities	Enablement	3.2 social support (practical)<br><br>21.2 restructuring of social environment
Concerns that the test might be **expensive** [QI]	PAT procedures at clinic	• Provide support to referral of patients from colleagues and practice managers<br>• Provide the PAT testing procedures	Social/Professional role and identity	Social opportunities	Enablement<br><br>Modelling	3.2 social support (practical)
Fear of **getting it wrong** and the safety patients [QI]	Training<br><br>PAT procedures in clinic	• Provide support to refer low risk patients to do PAT<br>• Provide evidence of safety of PAT	Social/Professional role and identity<br><br>Beliefs about consequences<br><br>Emotion	Social opportunities<br><br>Reflective motivation<br><br>Automatic motivation	Enablement<br><br>Modelling<br><br>Education<br><br>Persuasion	5.1 information about health consequences<br><br>5.6 information about emotional consequences
Perception that it is **safer to take patients' word for it** [QI]	"Penicillin Allergy Testing: Information for general practice" leaflet	• Provide evidence that only a small percentage of patients who think to be allergic actually are	Beliefs about consequences<br><br>Emotions	Reflective motivation<br><br>Automatic motivation	Education<br><br>Persuasion	5.1 information about health consequences

For peer review only

Fear of **getting it wrong** and **professional ramifications** [*QI]	Training<br><br>PAT procedures in clinic	• Provide support to refer low risk patients to do PAT	Social/Professional role and identity<br><br>Environmental context and resources<br><br>Emotion	Social opportunities<br><br>Physical opportunity<br><br>Automatic motivation	Enablement<br><br>Modelling	3.2 social support (practical)
**Lack of time** during consultation to discuss referral [RR]	Consultation with patients<br><br>Training	• Provide the opportunity to have a consultation with patients to specifically discuss PAT	Environmental context and resources<br><br>Beliefs about capabilities	Physical opportunities<br><br>Reflective motivation	Enablement	12.2 restructuring of social environment
Anticipation that **patients will not want** to have a reaction [RR]	Consultations with patients<br><br>"Penicillin allergy testing: going for a test" booklet	• Provide the opportunity to discuss with patients the risks of taking the test<br>• Provide pre-test booklet with information around the risks of PAT	Social influences	Social opportunity	Enablement	12.2 restructuring of social environment
**Forgetting to discuss** testing [RR]	Consultations with patients<br><br>Training	• Provide the opportunity to have consultations specifically around PAT	Behavioural regulation	Psychological capability	Enablement	3.2 social support (practical)
**Not knowing** that patients had an **allergy** [RR]	Consultations with patients	• Provide the opportunity to discuss their medical history with patients	Knowledge	Psychological capability	Training	2.7 feedback on outcomes of behaviour
**Lack of access** to testing [RR]	PAT testing procedures in clinic<br><br>"Penicillin Allergy Testing: Information for general practice" leaflet	• Provide the information that they can refer low risks patients to undertake PAT<br>• Provide information about where the test will be and what the stages are	Environmental Context and resources<br><br>Knowledge	Physical opportunity<br><br>Psychological capability	Enablement<br><br>Training	5.3 information of social and environmental consequences<br><br>15.1 verbal persuasion of capabilities

For peer review only

**Lack of ability in drawing a conclusion** on allergy status from medical history [RR]	"Penicillin Allergy Testing: Information for general practice" leaflet	• Provide information about the differences between drug reaction and allergy	Knowledge	Psychological capability	Training	4.2 information about antecedents
**Limited knowledge** of drug **allergies** [QI, RR]	"Penicillin Allergy Testing: Information for general practice" leaflet	• Provide information on how an allergy reaction is different than drug reaction	Knowledge	Psychological capability	Training	4.2 information about antecedents
**Poor knowledge** of allergy skin **testing** [RR]	"Penicillin Allergy Testing: Information for general practice" leaflet	• Provide information about skin testing (stage 2 of PAT), what the test will include, how patients will be monitored and how the results will be interpreted in relation to stage 3.	Knowledge	Psychological capability	Training	2.7 feedback on outcome of behaviour
**Lack of confidence** in dealing with allergies [RR]	"Penicillin Allergy Testing: Information for general practice" leaflet	• Provide information about penicillin allergy records and its implication	Beliefs about capabilities	Reflective motivation	Education<br><br>Persuasion	5.1 information about health consequences
Ready **availability of alternate antibiotics** [RR]	"Penicillin Allergy Testing: Information for general practice" leaflet	• Provide information on standards of care and use of broad-spectrum antibiotics	Beliefs about consequences	Reflective motivation	Education<br><br>Persuasion	5.1 information about health consequences
Awareness that **CCG are "cash strapped"** [QI]	PAT procedures at clinic	• Provide allergy testing at the hospital	Environmental context and resources	Environmental Context and resources	Physical opportunity<br><br>Enablement	12.2 restructuring of social environment
Worries about **costs of tests** [*QI]	PAT procedures at clinic	• Provide support in referring patients to PAT	Environmental context and resources	Environmental Context and resources	Physical opportunity<br><br>Enablement	12.2 restructuring of social environment

For peer review only

**Referral takes time** [QI]	PAT procedures at clinic<br>Training and WIs	• Provide guidance about referral and time of responses	Environmental context and resources	Environmental Context and resources	Physical opportunity<br><br>Enablement	4.1 instructions on how to perform a behaviour
**Patients not aware that the allergy label** may have a negative consequences [QI]	Consultations with patients	• Provide the opportunity to discuss the effects of inaccurate allergy labels	Social Influences	Social opportunity	Enablement	12.2 restructuring of social environment
Lack **of support from colleagues** in changing the label [QI]	Training and WIs	• Provide support from lead GPs and colleagues about changing the labels	Social influences	Social opportunity	Modelling	3.2 social support (practical)
**Experience of prescribing an alternative antibiotic** as routine [QI]	Training<br><br>"Penicillin Allergy Testing: Information for general practice" leaflet	• Provide information about how to refer low risks patients<br>• Provide information on the benefits of taking penicillin	Behavioural regulation	Psychological capability	Enablement	4.1 instruction on how to perform a behaviour
*Perception of how **easy finding an alternative antibiotic** is [QI]*	"Penicillin Allergy Testing: Information for general practice" leaflet	• Provide information about the benefits of taking penicillin<br>• Provide information about the effects of broad spectrum antibiotics	Knowledge	Psychological capability	Education<br><br>Enablement	5.1 information about health consequences
*Awareness that patients have been **historically over diagnosed** with penicillin allergy [QI]*	"Penicillin Allergy Testing: Information for general practice" leaflet<br><br>Training	• Provide information that incorrect penicillin allergy records are very common	Knowledge<br><br>Memory	Psychological capability	Education<br><br>Training	4.2 information about antecedents
*Awareness that **records might not be true** because patients do not remember any details of the allergy [QI]*	"Penicillin Allergy Testing: Information for general practice" leaflet	• Provide information about the reasons of incorrect penicillin allergy labels	Beliefs about capability	Reflective motivation	Education	4.2 information about antecedents

For peer review only

For peer review only - http://bmjopen.bmj.com/site/about/guidelines.xhtml

*Perception of **importance of referral** and penicillin allergy becoming more on the radar [RR]*	"Penicillin Allergy Testing: Information for general practice" leaflet<br><br>Training	• Provide information of the benefit of PAT and appropriate allergy labels	Reinforcement<br><br>Beliefs about consequences<br><br>Memory<br><br>Environmental context and resources	Automatic motivation<br><br>Reflective motivation<br><br>Physical opportunities	Incentivisation<br><br>Education<br><br>Persuasion<br><br>Training	5.1 information about health consequences
***Previous medical** and reaction **history** of patients [QI]*	Consultation with patients	• Provide the opportunity to discuss with patients their medical history and why the test will be relevant for them	Beliefs about capabilities	Reflective motivation	Enablement	12.2 restructuring of social environment
*Awareness that it is **important to prescribe** the most suitable antibiotic [QI]*	"Penicillin Allergy Testing: Information for general practice" leaflet<br><br>Training	• Provide information about the benefit of taking penicillin and about antimicrobial resistance	Reinforcement<br><br>Memory	Automatic Motivation<br><br>Psychological capability	Incentivisation<br><br>Training	4.2 information about antecedents
*Belief that **testing would lead to removal** of the allergy label [RR]*	"Penicillin Allergy Testing: Information for general practice" leaflet	• Provide information about how to update the patients records after receiving a negative test results<br>• Provide information about prescribing penicillin after a negative test	Reinforcement	Automatic motivation	Incentivisation	1.1 goal setting<br><br>1.4 action planning.<br><br>4.1 instructions on how to perform a behaviour
*Belief of **benefits of removing** the allergy label [RR]*	"Penicillin Allergy Testing: Information for general practice" leaflet	• Provide information about how incorrect penicillin allergy labels can be harmful for patients	Reinforcement<br><br>Beliefs about consequences	Automatic motivation<br><br>Reflective motivation	Incentivisation<br><br>Education<br><br>Persuasion	5.1 information about health consequences

For peer review only

Previous experience of referring patients *to PAT [QI]*	Consultations with patients<br><br>PAT procedures in clinic	• Provide the opportunity to explain to patients about PAT procedures and results<br>• Provide the PAT service	Beliefs about capabilities	Reflective motivation	Enablement	15.1 verbal persuasion about capability
Warning patients *about side effects at the time of prescription [QI]*	Consultations with patients<br><br>"Penicillin allergy testing: going for a test" booklet	• Provide the opportunity to discuss any side effects with the patients	Social/Professional role and identity<br><br>Beliefs about capabilities	Social opportunities<br><br>Reflective motivation	Enablement	12.2 restructuring of social environment
NICE guidelines on importance of PAT *and criteria for selecting patients for referral [LR]*	"Penicillin Allergy Testing: Information for general practice" leaflet<br><br>Training	• Provide information on the NICE guidelines on referring to PAT	Knowledge<br><br>Social influences	Psychological Capability<br><br>Social opportunity	Training<br><br>Enablement	9.1 credible source
Belief that ***penicillin allergy is an important*** *issue [RR]*	"Penicillin Allergy Testing: Information for general practice" leaflet	• Provide information on incorrect allergy labels, adverse patient outcomes, effects on antimicrobial prescribing, and on antimicrobial resistance	Reinforcement	Automatic motivation	Education<br><br>Persuasion	5.1 information about health consequences
Perception of ***support of CCG*** *[QI]*	"Penicillin Allergy Testing: Information for general practice" leaflet	• Provide information about the support from colleagues and guidelines for referral	Professional Role	Reflective motivation	Persuasion	9.1 Credible source
Previous experience of removing *or challenging the records *QI]*	PAT procedures at clinic	• Provide the opportunity to refer patients to ultimately change inaccurate labels	Behavioural regulation	Psychological capability	Enablement	15.1 verbal persuasion of capability
**Target behaviour: Clinician prescription of penicillin after negative test result**						

For peer review only

Doubts on **accuracy of allergy labels** in medical records [QI]	"Penicillin Allergy Testing: Information for general practice" leaflet	• Provide information that the oral challenge is the gold standard test for penicillin allergy	Knowledge	Psychological capability	Education<br><br>Persuasion	4.2 information about antecedents
**Worries of harming patients** [QI, RR]	"Penicillin Allergy Testing: Information for general practice" leaflet	• Provide information about safety of patients having penicillin after the test	Emotions<br><br>Beliefs about consequences	Automatic motivation<br><br>Reflective motivation	Education<br><br>Persuasion	5.1 information about health consequences<br><br>5.6 information about emotional consequences
**Lack of knowledge on penicillin** [QI, RR]	"Penicillin Allergy Testing: Information for general practice" leaflet	• Provide information about penicillin allergy and effects on standard of care	Memory	Psychological capability	Training	2.7 feedback on outcomes of behaviour
**System did not distinguish between intolerances and allergies** and may still show alerts [QI]	Pop-up alert WIs and allergy test results letter	• Provide information on the change of allergy status	Environmental context and resources<br><br>Beliefs about capabilities	Physical opportunity<br><br>Reflective motivation	Environmental restructuring<br><br>Enablement	12.5 adding objects to the environment
**Responsibility of changing the system** given to allergy clinic and lack of clarity on how to change them[QI]	Pop-up alert<br><br>Training, WIs and allergy test results letter	• Provide information on who will be responsible in changing the record	Social/professional role and identity<br><br>Beliefs about consequences<br><br>Behavioural regulation	Social opportunity<br><br>Reflective motivation<br><br>Psychological capability	Environmental restructuring<br><br>Enablement	4.1 instructions on how to perform a behaviour
**Lack of confidence** that penicillin could be safely administered [RR]	"Penicillin Allergy Testing: Information for	• Provide information about safety of taking penicillin when the test result is negative	Beliefs about consequences	Reflective motivation	Education persuasion	5.1 beliefs about health consequences

For peer review only

	general practice" leaflet					
**Lack of guidance** on how to interpret the results [QI]	"Penicillin Allergy Testing: Information for general practice" leaflet<br><br>Training, WIs, allergy test results letter	• Provide information on how the results will be presented to the clinicians and patients	Knowledge<br><br>Skills	Psychological capability<br><br>Cognitive skills	Education<br><br>Persuasion<br><br>Training	4.1 instructions on how to perform a behaviour<br><br>2.7 feedback on outcome of behaviour
Perception that **anxious patients may still not want to take penicillin,** and they would believe other people's advice instead [QI]	Consultation with patients<br><br>"Penicillin allergy testing: going for a test" booklet	• Provide the opportunity to discuss the accuracy of the results with patients	Beliefs about capability<br><br>Reinforcement<br><br>Social influences<br><br>Behavioural regulation	Reflective motivation<br><br>Automatic motivation<br><br>Social opportunity<br><br>Psychological capability	Enablement<br><br>Incentivisation	12.2 restructuring of social environment
**Lack of follow** up of the results [QI]	Pop-up alert Allergy test results letter	• Provide an alert when the results are available and the indication of a change of allergy label	Beliefs about consequences<br><br>Behavioural regulation	Reflective motivation<br><br>Psychological capability	Enablement<br><br>Environmental restructuring	12.4 adding object to the environment
Awareness **of different approaches in changing records** in different practices [QI]	Training , WIs, allergy test results letter	• Provide information of the new system in changing labels after PAT and how practices have been involved	Environmental context and resources<br><br>Behavioural regulation	Physical opportunity<br><br>Psychological capability	Environmental restructuring<br><br>Modelling	12.2 restructuring of the social environment<br><br>3.2 social support (practical)

For peer review only

Patients may worry less *about allergies [QI]*	Consultation with patients	• Provide the opportunity to reassure patients	Social influences	Social opportunity	Environmental restructuring	12.2 restructuring of social environment
Recognition that ***test provided evidence*** *that patient is not allergic [QI]*	"Penicillin Allergy Testing: Information for general practice" leaflet<br><br>PAT procedures at clinic	• Provide information about the accuracy of the test and who will perform it	Belief about consequences	Reflective motivation	Education<br><br>Persuasion	5.1 information about health consequences
Awareness that the ***test will allow prescribing first line*** *antibiotics and fight antibiotics resistance [QI]*	"Penicillin Allergy Testing: Information for general practice" leaflet	• Provide information about the effects of accurate penicillin allergy labels	Reinforcement	Automatic motivation	Incentivisation	2.7 feedback on outcomes of behaviour
Perception of ***penicillin allergy testing to be specialist advice*** *and to be objective evidence[QI]*	"Penicillin Allergy Testing: Information for general practice" leaflet	• Provide information of who will perform the test and interpret the results	Belief about consequences<br><br>Reinforcement	Reflective motivation<br><br>Automatic motivation	Education<br><br>Persuasion	5.1 information about consequences
Discussion of the negative test results *to address patients concerns about taking penicillin [QI]*	Consultations with patients	• Provide opportunity to discuss results with patients	Beliefs about capabilities<br><br>Social influences	Reflective motivation<br><br>Social opportunity	Enablement	5.1 beliefs about emotional consequences
Previous experience *of removing or challenging the records [QI]*	WIs, allergy test results letter	• Provide information about how to remove labels	Skills	Cognitive skills	Training	4.1 instructions on how to perform a behaviour
Perception that ***the system is easy to change*** *[QI]*	Pop-up alert, WIs and allergy test results letter	• Provide information about the change of label in the system	Beliefs about capabilities	Reflective motivation	Enablement<br><br>Environmental restructuring	12.5 adding objects to the environment

For peer review only - http://bmjopen.bmj.com/site/about/guidelines.xhtml

			Environmental context and resources	Physical opportunity		
Intention to change the label *[QI]*	Training, allergy test results letter	• Provide support and instructions on changing the label	Reinforcement	Automatic motivation	Incentive	2.7 feedback on outcomes of behaviour

Table S1b: Behavioural Analysis Table for the AlABAMA intervention, patient behaviours

**Barriers / *facilitators* to target behaviours**	**Intervention Component/s**	**Intervention ingredient**	**Theoretical Domains Framework (TDF)**	**Target construct (BCW)**	**Intervention function (BCW)**	**Behaviour Change Technique (using 93 BCT taxonomy v1)**
**Patient attendance at Penicillin Allergy Testing (PAT)**						
**Lack of information around the benefits of having access to penicillin**, when patients didn't experience any negative consequences of the allergy label [QI]	"Penicillin allergy testing: going for a test" booklet<br><br>Trial PIS	• Provide evidence on why penicillin is the preferred and best treatment for infections and kills fewer good bacteria;<br>• Provide information that other antibiotics could be less effective, and could increase the risk of "superbug" infections	Beliefs about consequences	Reflective Motivation	Education<br><br>Persuasion	5.1 information about health consequences
**Difficulty for patients to have access to the right antibiotics** if there is a penicillin allergy [QI]	PAT procedures at allergy clinic	• Provide opportunity to undertake allergy testing	Reinforcement	Automatic motivation	Environmental restructuring	12.2 restructuring of physical environment
Concerns related **to attending** the appointment [QI]	Consultations with clinicians;<br><br>Trial PIS	• Provide information about how the appointment will be arranged and that some travel expenses will be provided	Environmental Context and resources	Physical opportunity	Enablement	1.4 action planning

For peer review only

**Barriers / *facilitators* to target behaviours**	**Intervention Component/s**	**Intervention ingredient**	**Theoretical Domains Framework (TDF)**	**Target construct (BCW)**	**Intervention function (BCW)**	**Behaviour Change Technique (using 93 BCT taxonomy v1)**
Concerns whether the **GP will encourage/support** patient to have the test [QI]	Consultations with clinicians<br><br>Trial PIS	• Provide the opportunity to discuss the benefits of the testing with their clinicians	Social influences	Social opportunity	Enablement	3.1 social support (unspecified)
**Unsure about what the test is going to involve** and uncertainty around doses of penicillin given during the test [QI]	"Penicillin allergy testing: going for a test" booklet<br><br>Trial PIS	• Provide information about what a penicillin allergy test would involve, explaining the three stages of the testing	Knowledge;<br><br>Beliefs about consequences	Psychological capability<br><br>Reflective Motivation	Education	2.7 feedback on outcomes of behaviour<br><br>5.1 information about health consequences
**Lack of information about** the **credibility** of PAT [*QI]	Consultations with clinicians	• Provide the opportunity to address any concerns about what PAT is and what other options they may have	Knowledge;<br><br>Beliefs about consequences	Psychological capability<br><br>Reflective Motivation	Education	2.7 feedback on outcomes of behaviour<br><br>5.1 information about health consequences
**Concerns that the procedure is not safe** [QI]	"Penicillin allergy testing: going for a test" booklet<br><br>Trial PIS	• Provide information on the common reactions to PAT during the clinic visit and how patients will be treated by medical staff if they experience any reaction	Beliefs about consequences	Reflective Motivation	Persuasion	5.1 information about health consequences
**Concerns about a possible reaction** during test if they have been told by their clinicians for many years to avoid penicillin [QI]	"Penicillin allergy testing: going for a test" booklet	• Provide reassurance that the allergy test is very safe and that serious reactions are very rare.	Emotions	Automatic motivation	Persuasion	5.1 information about emotional consequences

For peer review only

**Barriers / *facilitators* to target behaviours**	**Intervention Component/s**	**Intervention ingredient**	**Theoretical Domains Framework (TDF)**	**Target construct (BCW)**	**Intervention function (BCW)**	**Behaviour Change Technique (using 93 BCT taxonomy v1)**
**Apprehension about having a test** for patients with a previous severe allergic reaction [QI]	"Penicillin allergy testing: going for a test" booklet<br><br>Trial PIS	• Provide reassurance that the test will be offered only if the nurses and doctors in the allergy clinic think that the patient is low risk	Emotions	Automatic motivation	Persuasion	5.1 information about emotional consequences
Concerns about a reaction that could **worsen their overall state of health** when patients have a chronic co-morbidity [QI]	Consultations with clinicians	• Provide reassurance about the safety of test	Beliefs about consequences;<br><br>Emotions	Reflective Motivation<br><br>Automatic motivation	Education<br><br>Persuasion	5.1 information about health consequences<br><br>5.6 information about emotional consequences
Perception of **skin testing as less frightening** than the oral challenge test [QI]	"Penicillin allergy testing: going for a test" booklet	• Provide information about the oral challenge test; Provide reassurance that the length of time spent at the clinic is long enough to check for any delayed reactions	Beliefs about consequences;<br><br>Emotions	Reflective Motivation<br><br>Automatic motivation	Education<br><br>Persuasion	2.7 feedback on outcomes of behaviour<br><br>5.1 information about health consequences
Patients **concerned about how they would be monitored** during the allergy test and whether doctors would respond quickly in case of reaction [QI]	"Penicillin allergy testing: going for a test" booklet<br><br>Trial PIS	• Provide information on how doctors and nurses will monitor patients during the skin testing and the oral challenge	Beliefs about consequences;<br><br>Emotions	Reflective Motivation<br><br>Automatic motivation	Education<br><br>Persuasion	5.1 information about health consequences

For peer review only

**Barriers / *facilitators* to target behaviours**	**Intervention Component/s**	**Intervention ingredient**	**Theoretical Domains Framework (TDF)**	**Target construct (BCW)**	**Intervention function (BCW)**	**Behaviour Change Technique (using 93 BCT taxonomy v1)**
**Worries about taking penicillin at home** following the allergy test in clinic and whether there is someone to call in case of reaction [QI]	"Penicillin allergy testing: going for a test" booklet	• Provide information on how the nurse will contact the patients after they have finished taking the penicillin at home to check whether they had any symptoms;<br>• Provide information of possible side effects when taking penicillin at home and that they are mild and do not require treatment<br>• Provide contact details of who to call at the clinic once they experience side effects at home	Beliefs about consequences<br><br>Emotions	Reflective Motivation<br><br>Automatic motivation	Education<br><br>Persuasion	5.1 information about health consequences
Perception that the procedure is **over medicalised** [QI]	"Penicillin allergy testing: going for a test" booklet<br><br>PAT procedures at allergy clinic	• Provide information on the test procedures and on how only the necessary tests will be taken: some of the stages will be skipped based on previous history of reactions and on reaction to the skin test (only necessary tests are carried out)	Beliefs about consequences<br><br>Knowledge	Reflective Motivation	Education<br><br>Persuasion	5.1 information about health consequences
**Lack of knowledge** that allergy status could change over time [RR]	"Penicillin allergy testing: going for a test" booklet	• Provide information about consequences of incorrect allergy label and the benefit of taking the test	Knowledge	Psychological capability	Education	4.2 information about antecedents
Primary care providers **did not inform them about the availability** of PAT [RR]	Consultations with clinicians<br><br>Invitation letter/Trial PIS	• Provide information about penicillin allergy labels and PAT	Knowledge	Psychological capability	Incentivisation<br><br>Education	2.7 feedback on outcome of behaviour

For peer review only

**Barriers / *facilitators* to target behaviours**	**Intervention Component/s**	**Intervention ingredient**	**Theoretical Domains Framework (TDF)**	**Target construct (BCW)**	**Intervention function (BCW)**	**Behaviour Change Technique (using 93 BCT taxonomy v1)**
Always having had a label of penicillin allergy [QI]	"Penicillin allergy testing: going for a test" booklet	• Provide information that once the allergy label is on the patient's medical record is rarely questioned and difficult to remove<br>• Provide information about the percentages of patients who really have a penicillin allergy and the reasons of incorrect allergy label.	Behavioural regulation<br><br>Beliefs about capabilities	Psychological capability<br><br>Reflective Motivation	Education<br><br>Persuasion	5.1 beliefs about health consequences
*Previous **experience of negative consequences** of having a penicillin allergy label and intentions to take penicillin [QI]*	Consultations with clinicians	• Provide an opportunity for patients to discuss with clinicians their previous medical history of penicillin allergy reactions and highlight their personal motivations to undertake the test	Emotion	Automatic motivation	Incentivisation	2.7 feedback on outcomes of behaviour
*Patient **awareness that penicillin is a first-line treatment** for many infections [QI]*	"Penicillin allergy testing: going for a test" booklet<br><br>Trial PIS	• Provide evidence on benefits of taking penicillin compared to broad spectrum antibiotics	Knowledge<br><br>Beliefs about consequences	Psychological capability<br><br>Reflective Motivation	Education	2.7 feedback on outcome of behaviour
*Feeling **relieved because allergy status has been confirmed** [QI]*	"Penicillin allergy testing: going for a test" booklet	• Provide information about the accuracy of allergy labels	Reinforcement	Automatic motivation	Incentivisation	2.7 feedback on outcome of behaviour
*Diagnosis is **an emotionally difficult event** and relief in getting an answer [QI]*	Consultations with clinicians<br><br>"Penicillin allergy testing: a negative test result" booklet	• Provide information on how they will get the results of the testing and provide opportunity to discuss it with GPs<br>• Provide information on the meaning on a negative test, who to inform about the test result and how to do so	Emotions	Automatic motivation	Persuasion	5.3 information about social and environmental consequences

**Barriers / *facilitators* to target behaviours**	**Intervention Component/s**	**Intervention ingredient**	**Theoretical Domains Framework (TDF)**	**Target construct (BCW)**	**Intervention function (BCW)**	**Behaviour Change Technique (using 93 BCT taxonomy v1)**
*Assurance of **access to trained medical staff** at the time of the test [QI]*	"Penicillin allergy testing: going for a test" booklet	• Provide reassurance that all medical staff are fully trained and will act immediately in case of allergy reaction	Beliefs about consequences;<br><br>Emotions	Reflective Motivation<br><br>Automatic motivation	Education<br><br>Persuasion	5.1 information about health consequences
*Perception of being **properly monitored** and of feeling safe during the test [QI]*	"Penicillin allergy testing: going for a test" booklet<br><br>PAT procedures at allergy clinic<br><br>Trial PIS	• Provide reassurance that the medical staff will monitor the patients during the test and will follow up after the patients will have finished the course at home	Beliefs about consequences<br><br>Emotions	Reflective Motivation<br><br>Automatic motivation	Education<br><br>Persuasion	5.3 information about social and environmental consequences
*Feeling **safe if the test takes place on hospital premises** [QI]*	"Penicillin allergy testing: going for a test" booklet<br><br>PAT procedures at allergy clinic<br><br>Trial PIS	• Provide information on where the allergy clinic is.<br>• Attending the PAT at the allergy clinic at the hospital	Knowledge	Psychological capability	Education	2.7 feedback on outcomes of behaviour
*Clear **expectations of what the procedure involves?** [QI]*	Consultations with clinicians;<br><br>"Penicillin allergy testing: going for a test" booklet<br><br>Trial PIS	• Provide information about the procedures of skin testing and oral challenge test	Knowledge	Psychological capability	Education	2.7 feedback on outcomes of behaviour<br><br>4.2 information about antecedents

For peer review only

**Barriers / *facilitators* to target behaviours**	**Intervention Component/s**	**Intervention ingredient**	**Theoretical Domains Framework (TDF)**	**Target construct (BCW)**	**Intervention function (BCW)**	**Behaviour Change Technique (using 93 BCT taxonomy v1)**
*Opportunity for **addressing any concerns** about the procedure [QI]*	Consultations with clinicians	• Provide the opportunity to discuss any concerns about taking the test	Environmental Context and resources;<br><br>Emotions	Physical opportunity<br><br>Automatic motivation	Enablement	12.2 restructuring of social environment
*Belief that PAT provides **valuable information** [RR]*	PAT procedures at allergy clinic;<br><br>"Penicillin allergy testing: a negative test result" booklet	• Provide information about penicillin allergy label<br>• Provide information about the meaning of negative test result and the safety of taking penicillin	Beliefs about consequences	Reflective motivation	Incentivisation	2.7 feedback on outcomes of behaviour
***Interest** in being tested [RR]*	PAT procedures at allergy clinic<br><br>"Penicillin allergy testing: going for a test" booklet	• Provide information about the benefit of taking the test	Reinforcement	Automatic motivation	Incentivisation	2.7 feedback on outcomes of behaviour
***Benefits** of the negative test results [QI]*	"Penicillin allergy testing: going for a test" booklet	• Provide information about the benefits of having a negative test results (being able to take penicillin, being less worries about penicillin, not having to go back to the GP with a reaction)	Reinforcement	Automatic motivation	Incentivisation	2.7 feedback on outcome of behaviour
***Importance of knowing** that the penicillin allergy is correct and that the penicillin allergy test is positive [*QI]*	Consultations with clinicians;<br><br>Invitation letter/Trial PIS	• Provide information about the benefits of accurate allergy labels	Reinforcement	Automatic motivation	Incentivisation	2.7 feedback on outcome of behaviour

For peer review only

**Barriers / *facilitators* to target behaviours**	**Intervention Component/s**	**Intervention ingredient**	**Theoretical Domains Framework (TDF)**	**Target construct (BCW)**	**Intervention function (BCW)**	**Behaviour Change Technique (using 93 BCT taxonomy v1)**
**Target behaviour: Patient consumption of penicillin when prescribed first line**						
**Perceived risk of clinicians' not prescribing** penicillin even after a negative result [QI]	"Penicillin allergy testing: a negative test result" booklet<br><br>Post-test intervention card	• Provide a card which will inform the clinicians of the change in allergy label<br>• Provide information on how the GP will be informed of the allergy test result	Social Influences	Social opportunities	Enablement	3.2 social support (practical)<br><br>12.2 restructuring of social environment
**Perceived risk of Re-labelling** of patients allergy even after negative test result [QI]	"Penicillin allergy testing: a negative test result" booklet<br><br>Post-test intervention card,<br><br>allergy test result letter	Provide information on how the GP will update the medical record of the patient and other medical systems with negative test results	Social influences	Social opportunities	Enablement	3.2 social support (practical)
Perception of test as **not accurate** [QI, RR]	"Penicillin allergy testing: a negative test result" booklet	Provide information that the test was very accurate in identifying whether the patients have a true penicillin allergy	Beliefs about consequences	Reflective motivation	Education<br><br>Persuasion	5.1 information about health consequences
**Severity** of past allergy reactions [QI]	PAT procedures at allergy clinic	Provide information that nurse or doctors at allergy clinic will only test patient who are perceived as low risk	Beliefs about consequences<br><br>Knowledge	Reflective motivation<br><br>Psychological capability	Education<br><br>Persuasion	4.3 information about antecedents

**Barriers / *facilitators* to target behaviours**	**Intervention Component/s**	**Intervention ingredient**	**Theoretical Domains Framework (TDF)**	**Target construct (BCW)**	**Intervention function (BCW)**	**Behaviour Change Technique (using 93 BCT taxonomy v1)**
**Patient Belief that they are still allergic** to penicillin after a negative text result [RR]	"Penicillin allergy testing: a negative test result" booklet	Provide information about the reliability of the test	Beliefs about consequences	Reflective motivation	Education<br><br>Persuasion	5.1 beliefs about consequences
**Fear of having an allergic reaction** again [RR]	"Penicillin allergy testing: a negative test result" booklet	Provide information about low risks of having an allergic reaction in the future	Emotion	Automatic Motivation	Education<br><br>Persuasion	5.6 beliefs about emotional consequences
**Lack of confidence** that penicillin could be safely administered [RR]	"Penicillin allergy testing: a negative test result" booklet	Provide information about the meaning of test results and which antibiotics are now safe to use	Beliefs about consequences	Reflective motivation	Education<br><br>Persuasion	5.1 beliefs about consequences
**Lack of knowledge of which antibiotics they could take** safely [RR]	"Penicillin allergy testing: a negative test result" booklet	Provide a list of antibiotics that patients with a negative test result can take	Knowledge	Psychological capability	Education	4.2 information about antecedents
**Distrust in health care providers'** advice if they don't prescribe penicillin even after the negative test result [RR]	Post-test intervention card	Provide card to show to health care professionals which gives details about the allergy test completed and explains that the patient had a negative test result	Reinforcement	Automatic motivation	Environmental restructuring	12.2 restructuring of social environment<br><br>12.5 adding objects to the environment
**Lack of knowledge about how to interpret skin test results** [RR]	Post-test intervention card	Provide explanation of the meaning of negative test results and what to do the next time they will need antibiotics	Knowledge	Psychological capability	Education	4.2 information about antecedents
**No differences in concerns** between patients who were investigated through skin test or oral test [RR]	"Penicillin allergy testing: a negative test result" booklet	Provide information about the accuracy of the test completed	Beliefs about consequences	Reflective motivation	Education<br><br>Persuasion	5.1 beliefs about consequences

Barriers / *facilitators* to target behaviours	Intervention Component/s	Intervention ingredient	Theoretical Domains Framework (TDF)	Target construct (BCW)	Intervention function (BCW)	Behaviour Change Technique (using 93 BCT taxonomy v1)
Perception of the test as ***definitive answer*** *and proof of allergy status [QI]*	PAT procedures at allergy clinic;<br><br>“Penicillin allergy testing: a negative test result” booklet<br><br>Post-test intervention card<br><br>allergy test result letter	• Provide the test results from the allergy clinic<br>• Provide information on the meaning of the negative test result and information about the accuracy of the test<br>Provide information about reliability of test	Reinforcement	Automatic motivation	Environmental restructuring	2.7 feedback on outcomes of behaviour
Perception to have undergone a ***thorough testing procedure*** *[QI]*	PAT procedures at allergy clinic	Monitoring of the patients by the nurse or doctor at allergy clinic who will provide medical assistance when needed	Reinforcement	Automatic motivation	Environmental restructuring	12.2 restructuring of the social environment
Lack of reaction *following the test [QI]*	PAT procedures at allergy clinic<br><br>“Penicillin allergy testing: going for a test” booklet	Provide information about the type of reactions and their indication on the state of the allergy	Reinforcement	Automatic motivation	Environmental restructuring	12.1 restructuring of the physical environment

S2. Think-aloud topic guides for clinicians and patients

"Penicillin Allergy Testing: Information for general practice" leaflet

In this feedback session, I am interested in what you think about when you read the information provided in the booklets. I would like you to think aloud as you read through each section, if you have any comments. There is no right or wrong answers. So when you read a section of the booklet, if you could tell me what thoughts come to your mind when you read it.

1. Looking at the Penicillin allergy" and "Incorrect penicillin allergy records are very common what did you think after reading it? Did you think it was comprehensive/ new information/ useful information
2. Going through the section on "Incorrect penicillin allergy records can be harmful for patients what did you think while reading it? What did this section tell you? Did you find it convincing?
3. Looking at the section "Penicillin allergy testing -What does the test involve?", was it clear to you what the testing involves? Would you feel confident in explaining the process to patients?
4. What do you think about the section: "How accurate is the test"? How do you understand this section? Is there anything unclear?
5. What do you think about the section: "Are there any risks to patients having the test?" Do you feel reassured by this section? Would you feel confident to refer patients for testing based on this information?
6. What do you think about the section: "How should I discuss the test with patients? "? Do you think this is an important section? Is the description clear?
7. What do you think about the section: "Updating medical records"? Is it clear what you need to do?
8. What do you think about the section: "Prescribing penicillin after a negative test"? Would you feel confident to prescribe penicillin after reading this section?

- What are your overall thoughts on the leaflet?
- How can this leaflet be improved?
- What will stick in your mind from this leaflet?
- How easy or difficult was it to read/ understand this leaflet?
- Is there anything which you think is missing?
-

Intervention Card

- What do you think about this card?

For peer review only

- Can you tell me how do you understand the last sentences: that the test was negative which means that the patient is not allergic
- What does: with the same risk of allergy as the general population mean to you?

Can you tell me what you would do if a patient came with this card

"Penicillin allergy testing: going for a test" booklet

In this feedback session, I am interested in what you think about when you read the information provided in the booklets. I would like you to think aloud as you read through each section, if you have any comments. There is no right or wrong answers. So when you read a section of the booklet, if you could tell me what thoughts come to your mind when you read it.

9. Looking at the front cover the writing and the pictures, what did you think this leaflet was going to be about?
10. Looking at page 3, with a list of contents, what did you think after reading it? Did you at that point think that it was comprehensive?
11. Going through the first section: "How do I know if I am allergic to penicillin", what did you think while reading it? What did this section tell you? Why do you think this section is here?
Specifically, there is some numerical information provided here about how many people are allergic etc, can you tell me in your own words what you understand by this paragraph?
12. Going through the next paragraph on why is it important for people to have a penicillin allergy test, again what did you think while reading it? Were you convinced by the argument? How do you understand this section?
13. Going through the next section: "what does a penicillin allergy test involve", what do you think of this section? Could you talk me through what you think you would have to do if you had this test? Is there anything unclear? Is there anything missing? What do you think about the test and what is required of you.
14. Going through the next section: "Are there any risks", what was going through your head when you were reading that? What were you feeling when you have read that? How would you feel about going for a test after reading this section? Specifically, this section tell you what symptoms people may get- what did you think while reading this section? What is this section telling you? How would you put this section in your own words?

- What are your overall thoughts on the leaflet?
- How can this leaflet be improved?
- What will stick in your mind from this leaflet?

For peer review only

- How easy or difficult was it to read/ understand this leaflet?

"Penicillin allergy testing: a negative test result" booklet

1. Looking at the front cover the writing and the pictures, what did you think this leaflet was going to be about? How do you understand the heading: Negative test result? What did you think about the picture?
2. Looking at the table of contents, what did you think after reading it? Did you at that point think that it was comprehensive?
3. Going through the next section: "what does a negative test result mean", what was going through your head when you were reading that? How do you understand this section? What do you think about the picture used here?
4. Going through the next section: "Is it safe for me to now take penicillin", what were you thinking while going through this section?
5. Going through the next section: "What should I do now?" How do you understand this section? What were you thinking reading this section? Can you tell me in your words what you are asked to do?
6. Going through the next section: "What should I do next time I have an infection?", how do you understand this section?

- What are your overall thoughts on the leaflet?
- How can this leaflet be improved?
- What will stick in your mind from this leaflet?
- How easy or difficult was it to read/ understand this leaflet?

Intervention Card

- What do you think about this card?
- Can you tell me how do you understand the last sentences: that the test was negative which means that the patient is not allergic
- What does: with the same risk of allergy as the general population mean to you?
- Can you tell me in what situations you think you may use it?

For peer review only

S3. Table of changes: "Penicillin Allergy Testing: going for a test" booklet

Suggested changes	Changes made	Reasons for change	Priority for change
**Front page**			
The image on the cover of the booklet must be changed as it does not relate to what happens during penicillin allergy testing; also the blood samples on the GP desk look scary.	A new image was selected showing a more friendly looking consultation between patient and GP without any medical equipment	EO	Must do
The logos could be put at the bottom of the cover pager (rather than at the top) as they distract from the image)	The logos are on the bottom of the page	EO	Could do
The participants suggested making the booklets even easier to follow by adding text boxes throughout to separate big chunks of text.	The booklet was made more visually appealing	EO	Could do
**How can this booklet help me?**			
The contents page looks a bit dense on the page. The participants did not like the "chemical symbols" image in the background design.	• Better use of the space on the contents page was made and the text more was spread across the page.<br>• The introduction to the booklet was put in a separate box to make it look more manageable. Chemical symbols image were removed.	EO	Should do

For peer review only

Suggested changes	Changes made	Reasons for change	Priority for change
**How do I know if I am allergic to penicillin?**			
The participants suggested putting the last sentence of this section: "A penicillin allergy test is the safest way to find out whether or not you are allergic to penicillin" in bold.	The last sentence was put in bold	EO, QI	Must do
Statistics about the prevalence of allergy were not always understood by the participants, as the participants often thought that 1 in 10 people are allergic. They wanted a more visual presentation of this key information	An infographic was created showing how many people think they are allergic and how many people actually are allergic. The image of a GP taking blood pressure was replaced by the infographic.	EO	Should do
This section looks also a bit dense on the page	The text was spread across to the following page	EO	Should do
**Why is it important for people to have a penicillin allergy test?**			
Participants were unsure what narrow and broad spectrum antibiotics were and did not recognise MRSA abbreviation.	• The reference to narrow and broad spectrum antibiotics was removed<br>• The full name for MRSA was presented	RT, CT	Must do
Participants did not always know that penicillin is more than one antibiotic. This message should be made clearer.	An additional sentence was added to provide information that penicillin includes a number of different antibiotics	EO, QI	Must do

For peer review only

Suggested changes	Changes made	Reasons for change	Priority for change
Booklet 2 mentioned that penicillin is good at treating throat infections- this information was missing from this booklet.	A sentence was added to explain that “antibiotics are very useful to treat many infections, including ear, skin, throat and chest infections”	EO, QI	Must do
The information was slightly repetitive and perhaps the section should start with the strongest message (which participants thought was that penicillin are effective).	• The section was made more concise.<br>• The first paragraph includes information on what penicillins are, what they are used for, and that they are considered the best treatment for infections	EO	Should do
Other sentences in this section, for example: “other antibiotics may not be as effective” could also be put in bold.	The sentence was put in bold	EO, QI	Should do
The section looks a bit dense.	The text was spread across the previous page	EO	Should do
**What does a penicillin allergy test involve?**			
Participants wanted to know if they can call the allergy clinic and whether they will be provided a number and what details the doctor or nurse will be checking (Why they would not have to be checked in person?).	It reads: “you will be provided with details of who to call if you have any symptoms or concerns”	RT, CT, QI	Must do

For peer review only

Suggested changes	Changes made	Reasons for change	Priority for change
Participants wanted to have reassurance that 3 days would be enough to detect delayed reactions.	It reads: "this is long enough to check for any delayed reactions"	RT, CT, QI	Must do
Participants suggested making it clearer that the clinic is in the hospital.	This sections starts with "Penicillin allergy testing takes place in a hospital, in a specialist allergy clinic". The sentence was put in bold	RT, CT, QI	Must do
Participants had queries about symptoms they could experience after three days of taking penicillin as the booklet said: "mild and mostly side effects"; they wanted clarification whether these could be allergic reaction symptoms	It reads: "these symptoms are generally mild and do not require treatment"	RT, CT, QI	Must do
Participants did not always like the phrase "like a mosquito bite" (skin testing stage) as some people may have an "unpleasant reaction to mosquitos".	No change made – comment from one participant, felt by clinical team to be best way to describe and usual term used in current NHS leaflets.	RT, CT	Could do
There is a lot of text on one page	The descriptions of the test was spread over two pages	EO	Should do
A diagram alongside the stages of the test could be presented to make it even clearer	A diagram was provided for the three stages of testing and the description of the test was spread over two pages	EO	Could do
**Are there any risks to having the test?**			

For peer review only

Suggested changes	Changes made	Reasons for change	Priority for change
The participants wanted to have a separate paragraph on what can happen during the test and what can happen during 3 days of taking penicillin at home.	There are two paragraphs, one on what could happen during the clinic visit, and one about what could happen at home	RT, CT	Must do
They wanted clarification whether the listed symptoms for taking penicillin at home: "sickness, a mild, small rash, nausea, upset stomach, or other mild symptoms" were symptoms of an allergic reaction; this was especially confusing as feeling sick was also listed as part of the allergic reaction when taking penicillin in the clinic (you might feel sick, itchy, develop hives ('nettle rash'), swelling, a fast heartbeat, dizziness or difficulty breathing.	It reads: "Once you go home, it is possible you may develop mild symptoms such as sickness, a mild, small rash, nausea, upset stomach, or other mild symptoms. It is extremely unlikely that you will have any serious symptoms, as these would have happened when you took penicillin during the clinic visit"	RT, CT, QI	Must do
The participants suggested putting the phrase: "extremely unlikely" in bold ("It is extremely unlikely that you will have any serious symptoms, as these would have happened when you took penicillin during the clinic visit".	The phrase was put in bold	EO	Could do

Note: EO: patient; RT: research team; CT: clinical team; QI: qualitative interviews

For peer review only

S4. Full description of the AlABAMA (ALlergy AntiBiotics And Microbial resistAnce) Intervention package in accordance with the Template for Intervention Description and Replication (TIDieR) framework.

- **1. BRIEF NAME:** AlABAMA (ALlergy AntiBiotics And Microbial resistAnce) Intervention package
- **2. WHY:** Penicillins are the most prescribed antibiotics and they are used as first-line therapy for many infections. Side effects and symptoms related to the infections are commonly mislabelled as allergy. Around 6% of people think that they are allergic to penicillin but fewer than 10% of these patients are truly allergic. As a consequence a significant proportion of the population have restricted access to highly effective penicillins, and are prescribed broad-spectrum antibiotics instead, which may be less effective and could increase the risks of acquiring multi-drug resistant bacteria. The goal of AlABAMA is to introduce a "pre-emptive" penicillin allergy assessment pathway (PAAP) for patients who are more likely to receive antibiotics that could impact antibiotic prescribing, have patients benefits, limit antimicrobial resistance (AMR) infections and deliver NHS cost savings. The AlABAMA approach will develop a "one stop shop" single Penicillin Allergy Test (PAT) at the hospital clinic for specialist immunology assessment (skin testing and/or oral challenge test).
- **3&4: WHAT:** AlABAMA is a complex behaviour change intervention which targets prescribers in general practice and low risk patients with a penicillin allergy record. See Table 2 for an overview of the key intervention components. For more details about the specific behaviour change techniques used, refer to the behavioural analysis.
- **5. WHO PROVIDED**: The AlABAMA research team will provide intervention materials to general practices taking part in the feasibility and main trial. Practices will be responsible for local implementation by the practice manager(s). GPs will have consultations with patients to check eligibility for entering the trial.
  Research nurses at the hospital will be responsible for sending the two intervention booklets to patients in the intervention arm by post. Research nurses at the immunology hospital clinic will deliver Stage 1, Stage 2 and/or stage 3 of the PAT.

For peer review only

- **6. HOW**: The intervention will be delivered mainly by written information for prescribers and for patients in the intervention arm. The site training will be delivered to all the GP leads and the practice manager(s) in one session per practice. Prescribers will receive information by email. Patients randomised to the PAAP intervention arm will receive the "Penicillin Allergy Testing: going for a test" by post. Patients will complete Stage 1 and Stage 2 (skin test) and /or Stage 3 (oral challenge test) in a single hospital visit which lasts around 3 hours. Patients with a negative PAT result will receive the "Penicillin Allergy Testing: a Negative Test Result" booklet by post. Prescribers will receive the update about the penicillin allergy test result by post and electronically.
- **7. WHERE**: AlABAMA is intended for implementation in general practices, where the site training and patient consultation also take place. The PAT takes place at the immunology clinic of a local hospital. Written information for clinicians is provided electronically subsequently to the site training. Written information for patients is sent by post by research nurses at the hospital.
- **8. WHEN and HOW MUCH**: Site training is provided by the AlABAMA research team prior to the screening and eligibility assessment of patients. Patients randomised to the PAAP trial arm will be sent the "Penicillin Allergy Testing: going for a test" booklet with their hospital appointment letter. Patients randomised to the control arm will receive usual clinical care. All patients completing the PAT will receive a letter with the test result after their appointment. Patients who have tested negative will receive the "Penicillin Allergy Testing: a Negative Test Result" booklet and Intervention card by post. Practices will be informed of the test result and instructed to update the patients' electronic health record.
- **9. TAILORING**: The implementation of behavioural and clinical elements of the AlABAMA intervention package will be implemented in all GP practices and local immunology clinics participating in the trial.
- **10. MODIFICATIONS**: Modifications were made to the intervention materials following qualitative feedback from the feasibility study.
- **11-12. HOW WELL**: The intervention is being tested in a feasibility trial in 8 general practices. It will then be tested in a randomised control trial across 70 GP practices.

For peer review only

S5 GUIDED – a guideline for reporting for intervention development studies.

Item description	Page in manuscript where item is located
1. Report the context for which the intervention was developed.	4
2. Report the purpose of the intervention development process.	5
3. Report the target population for the Intervention development process.	4
4. Report how any published intervention development approach contributed to the development process	5
5. Report how evidence from different sources informed the intervention development process.	6
6. Report how/if published theory informed the intervention development process.	11
7. Report any use of components from an existing intervention in the current intervention development process.	5
8. Report any guiding principles, people or factors that were prioritised when making decisions during the intervention development process.	9
9. Report how stakeholders contributed to the Intervention development process.	8
10. Report how the intervention changed in content and format from the start of the intervention development process.	14-15
11. Report any changes to interventions required or likely to be required for subgroups.	15-17
12. Report important uncertainties at the end of the intervention development process.	21-22

For peer review only

13. Follow TIDieR guidance when describing the developed intervention.	18
14. Report the intervention development process in an open access format.	1

For peer review only
